# Precision Methylome and *In Vivo* Methylation Kinetics Characterization of *Klebsiella pneumoniae*

**DOI:** 10.1016/j.gpb.2021.04.002

**Published:** 2021-06-29

**Authors:** Jing Fu, Ju Zhang, Li Yang, Nan Ding, Liya Yue, Xiangli Zhang, Dandan Lu, Xinmiao Jia, Cuidan Li, Chongye Guo, Zhe Yin, Xiaoyuan Jiang, Yongliang Zhao, Fei Chen, Dongsheng Zhou

**Affiliations:** 1 CAS Key Laboratory of Genome Sciences and Information, Beijing Institute of Genomics, Chinese Academy of Sciences and China National Center for Bioinformation, Beijing 100101, China; 2 Department of Oncology, Henan Provincial People’s Hospital, People’s Hospital of Zhengzhou University, People’s Hospital of Henan University, Zhengzhou 450001, China; 3 University of Chinese Academy of Sciences, Beijing 100049, China; 4 Department of Medical Research Center, Peking Union Medical College Hospital, Peking Union Medical College & Chinese Academy of Medical Sciences, Beijing 100730, China; 5 State Key Laboratory of Pathogen and Biosecurity, Beijing Institute of Microbiology and Epidemiology, Beijing 100071, China; 6 CAS Key Laboratory of Genomic and Precision Medicine, Beijing Institute of Genomics, Chinese Academy of Sciences and China National Center for Bioinformation, Beijing 100101, China

**Keywords:** DNA methylation, Epigenomics, 6mA, 5mC, Epigenetic regulation

## Abstract

*Klebsiella pneumoniae* (*K. pneumoniae*) is an important pathogen that can cause severe hospital- and community-acquired infections. To systematically investigate its methylation features, we determined the whole-genome sequences of 14 *K. pneumoniae* strains covering varying serotypes, multilocus sequence types, clonal groups, viscosity/virulence, and drug resistance. Their methylomes were further characterized using Pacific Biosciences single-molecule real-time and bisulfite technologies. We identified 15 methylation motifs [13 *N*6-methyladenine (**6mA**) and two 5-methylcytosine (**5mC**) motifs], among which eight were novel. Their corresponding DNA methyltransferases were also validated. Additionally, we analyzed the genomic distribution of G**A**TC and C**C**WGG methylation motifs shared by all strains, and identified differential distribution patterns of some hemi-/un-methylated G**A**TC motifs, which tend to be located within intergenic regions (IGRs). Specifically, we characterized the *in vivo* methylation kinetics at single-base resolution on a genome-wide scale by simulating the dynamic processes of replication-mediated passive demethylation and MTase-catalyzed re-methylation. The slow methylation of the G**A**TC motifs in the replication origin (*oriC*) regions and IGRs implicates the epigenetic regulation of replication initiation and transcription. Our findings illustrate the first comprehensive dynamic methylome map of *K. pneumoniae* at single-base resolution, and provide a useful reference to better understand **epigenetic regulation** in this and other bacterial species.

## Introduction

*Klebsiella pneumoniae* (*K. pneumoniae*), an important member of the Enterobacteriaceae, can cause severe hospital- and community-acquired infections such as pneumonia, genitourinary tract infection, and septicaemia. There are various typing methods for *K. pneumoniae* strains, including serotyping, multilocus sequence typing (MLST), and clonal group (CG) typing [1], [2]. Studies indicate that the hypervirulence phenotype usually corresponds to K1/K2/K57 serotypes and CG23-ST23 [1], [2], [3], while the multidrug resistance (MDR) phenotype often corresponds to CG258-ST11/ST258 [4].

Studies on DNA methylation in *K. pneumoniae* strains using molecular biological techniques identified three DNA methyltransferases (MTases) and corresponding motifs, including two restriction-modification (R-M) systems (M.KpnI: GGT**A**CC; M.KpnBI: CAA**A**N_6_RTCA, where N = A or C or G or T and R = G or A) and one orphan MTase (Dam: G**A**TC) [5], [6], [7]. In each motif, the methylated nucleotide is shown in bold, and the nucleotide pairing with the methylated nucleotide on the complementary strand is marked with an underline. Further research on Dam revealed the epigenetic mechanism involved in regulating mismatch repair, virulence, and pathogenicity of *K. pneumoniae* strains [8].

Recent rapid progress on high-throughput sequencing techniques, such as Pacific Biosciences (PacBio) single-molecule real-time (SMRT) sequencing for accurate detection of modified bases [mainly *N*6-methyladenine (6mA)] on a genome-wide scale, and bisulfite sequencing for efficient analysis of genome-wide 5-methylcytosine (5mC) [9], [10], has greatly facilitated DNA methylome investigations in bacteria. It is well known that 6mA and 5mC are the two most important types of DNA methylation in prokaryotes [9]. Numerous bacterial methylomes have been precisely determined using the aforementioned two techniques, including *Escherichia coli*, *Mycoplasma genitalium*, *Bifidobacterium breve*, *Clostridium difficile*, *Campylobacter jejuni*, *Helicobacter pylori* [9], [10], [11], [12], [13], and *Mycobacterium tuberculosis* complexes (MTBC) reported by our group [14].

By precisely and comprehensively analyzing the bacterial methylome, a lot of valuable information has been obtained, including methylation motifs and their corresponding MTases, motif distributions in genomes, and related epigenetic regulatory mechanisms in bacteria [9], [15], [16]. Most identified MTases and their corresponding motifs belong to the R-M system, which primarily functions to disrupt (cleave) invading DNA and protect genomic DNA through methylation-mediated mechanisms [9], [10], [11], [12], [13], [14]. Distinctively, some orphan MTases (without cognate restriction enzymes) and their corresponding motifs perform multiple epigenetic regulatory functions in bacteria [15], [16], [17], [18]. Among them, the Dam/G**A**TC motif is the most well-known due to its presence in almost all Enterobacteriaceae bacteria, and its involvement in the epigenetic regulation of replication, transcription, and mismatch repair [15], [16], [17], [19], [20], [21]. In particular, its regulatory role in replication initiation has been studied in *E. coli*. Its replication origin (*oriC*) region contains five DnaA boxes and 11 G**A**TC motif sites. Replication-mediated passive demethylation causes the hemi-methylated G**A**TC motifs adjacent to the DnaA boxes to be specifically recognised and bound by SeqA, leading to competition for the motif sites between Dam and SeqA [17]. As a result, re-methylation of the motifs is delayed, which in turn prevents the initiation cascade for chromosome replication induced by the DnaA protein [17], [19]. Re-methylation of the upstream G**A**TC motifs of the third and fifth DnaA boxes are the rate-limiting steps for DNA replication initiation in *E. coli* strains [17]. Additionally, Dam also participates in the transcriptional regulation of downstream genes such as *opvAB* in *Salmonella enterica* [15].

Although several MTases and corresponding motifs have been revealed in *K. pneumoniae* strains, the whole methylome has not yet been reported. Herein, we obtained whole-genome sequences of 14 *K. pneumoniae* strains of various types, and characterized their methylomes using SMRT/bisulfite sequencing. A total of 15 methylation motifs were identified, including 13 6mA and two 5mC methylation motifs. Among them, eight motifs were novel, corresponding to eight novel MTases [*K. pneumoniae* adenine methyltransferases A–G (KamA–G) and *K. pneumoniae* cytosine methyltransferase A (KcmA)]. We further analyzed the distribution patterns of the G**A**TC and C**C**WGG (where W = A or T) methylation motifs shared by all *K. pneumoniae* strains. Importantly, by establishing a mathematical model to simulate the dynamic processes of passive demethylation and re-methylation for each motif in the exponential phase, we characterized the genome-wide *in vivo* methylation kinetics at single-base resolution. Motifs at different genomic locations displayed different re-methylation rates, and the G**A**TC motifs in the *oriC* regions and intergenic regions (IGRs) had slow re-methylation rates. Our findings indicate potential roles of epigenetic regulation in replication initiation and transcription in the *K. pneumoniae* genome, and provide important insight into *K. pneumoniae* epigenomics.

## Results

### General bioinformatic analysis of 14 *K. pneumoniae* strains

**Figure 1:**
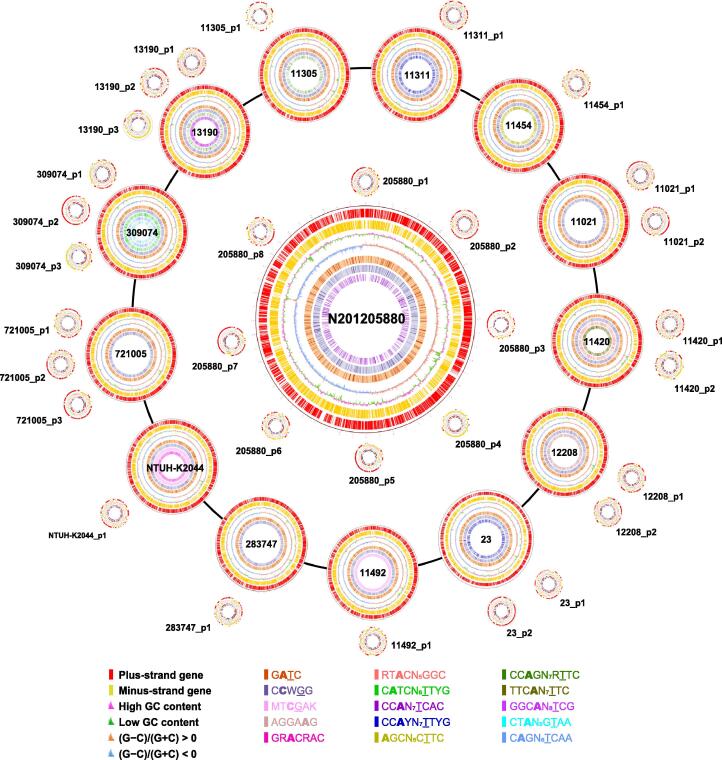
**Circos plots displaying general genomic information for 14*K. pneumoniae* strains** The circles are as follows (from outside to inside): 1) physical map scaled in megabases from base 1, the start of the putative replication origin; 2) coding sequences transcribed in a clockwise direction; 3) coding sequences transcribed in a counterclockwise direction; 4) GC content based on a 2-kb sliding window (red and green indicate a GC content higher or lower than average, respectively); 5) GC skew [(G−C)/(G+C)] based on a 2-kb sliding window (orange and green indicate GC skew above and below zero, respectively); 6) G**A**TC motifs; and 7) C**C**WGG motifs. The other circles inside indicate the other 13 methylation motifs. In each motif, the methylated nucleotide is shown in bold, and the nucleotide pairing with the methylated nucleotide on the complementary strand is marked with an underline. Degenerate bases used in the motifs are as follows: R = G or A, Y = C or T, M = A or C, K = G or T, S = G or C, W = A or T, B = C or G or T, D = A or G or T, H = A or C or T, V = A or C or G, N = A or C or G or T. Mb, megabase.

We first obtained the whole-genome sequences of 14 *K. pneumoniae* strains (including NTUH-K2044, 11492, 11420, 11454, 12208, 11311, 23, 11305, N201205880, 309074, 13190, 283747, 721005, and 11021) differing in terms of serotypes, MLSTs, CGs, viscosity/virulence, and drug resistance (Tables S1–S3) by SMRT sequencing followed by correction using Illumina sequencing (Figure 1). We then constructed a phylogenetic tree using 76 complete genomes of *K. pneumoniae* strains (14 from the current work and 62 obtained online) (Figure S1; Table S4). Our 14 *K. pneumoniae* strains covered many common CGs and MLSTs of *K. pneumoniae* strains in China (*e.g*., CG23-ST23 and CG258-ST11) [22], indicating good representation and diversity of *K. pneumoniae* strains selected in our study.

The bioinformatic analyses provided general genome information (Table 1, Figure 1), including genome size (5.20–5.50 Mb), GC content (57.37%–57.68%), predicted number of protein-coding genes (4990–5468), gene length (886–922 bp), and the percentage of coding regions (88.26%–90.23%) [23]. In addition, each *K. pneumoniae* strain contained 1–8 plasmids, with lower GC content (45.72%–54.85%), lower percentage of coding regions (75.98%–88.63%), and shorter average gene length (574–831 bp) (Table 1).

**Table 1:** **General genomic information for the 14 *K. pneumoniae* strains**

Strain name	**Chromosome/plasmid**	**Genomic size (Mb)**	**GC content (%)**	**No. of genes**	**Average gene** **size (bp)**	**Coding region (%)**	**No. of** **tRNAs**	**No. of** **rRNAs**
NTUH-K2044	NUTH-K2044_chr	5.25	57.68	5050	917	90.09	86	25
	pNUTH-K2044-1	0.22	50.17	243	701	83.28	0	0
11492	11492_chr	5.25	57.45	5017	922	88.43	87	25
	p11492-1	0.19	50.37	202	724	82.72	0	0
11420	11420_chr	5.44	57.46	5264	912	88.35	88	25
	p11420-1	0.23	50.10	257	688	83.43	0	0
	p11420-2	0.08	50.56	101	605	84.45	0	0
11454	11454_chr	5.20	57.60	4990	917	88.35	86	25
	p11454-1	0.21	50.67	225	717	83.74	0	0
12208	12208_chr	5.21	57.59	5067	910	89.42	82	25
	p12208-1	0.33	47.27	353	709	83.79	0	0
	p12208-2	0.21	49.69	232	698	82.90	0	0
11311	11311_chr	5.27	57.56	5048	918	88.26	86	25
	p11311-1	0.20	49.93	209	733	82.74	0	0
23	23_chr	5.29	57.54	5120	910	88.31	85	25
	p23-1	0.20	49.93	212	730	82.76	0	0
	p23-2	0.11	49.46	106	831	87.78	2	0
11305	11305_chr	5.32	57.46	5186	901	88.37	88	25
	p11305-1	0.06	53.29	60	690	75.98	0	0
N201205880	N201205880_chr	5.21	57.50	5106	901	88.85	87	25
	pN201205880-1	0.23	51.08	240	758	82.03	0	0
	pN201205880-2	0.15	52.33	146	755	88.63	0	0
	pN201205880-3	0.11	54.85	134	661	83.30	0	0
	pN201205880-4	0.11	49.31	108	815	88.07	1	0
	pN201205880-5	0.08	50.29	88	733	84.69	0	0
	pN201205880-6	0.08	54.00	96	596	78.08	0	0
	pN201205880-7	0.07	52.85	83	657	86.33	0	0
	pN201205880-8	0.06	52.45	69	609	82.19	0	0
309074	309074_chr	5.31	57.46	5181	907	90.23	89	25
	p309074-1	0.19	53.44	197	735	83.60	0	0
	p309074-2	0.07	51.83	85	668	83.78	0	0
	p309074-3	0.05	49.03	65	667	85.67	0	0
13190	13190_chr	5.26	57.50	5172	897	89.34	88	25
	p13190-1	0.31	47.26	316	703	84.09	0	0
	p13190-2	0.08	53.86	85	649	79.42	0	0
	p13190-3	0.04	45.72	50	674	86.66	0	0
283747	283747_chr	5.50	57.37	5441	891	90.07	85	25
	p283747-1	0.15	54.40	192	574	78.82	0	0
721005	721005_chr	5.48	57.38	5468	886	89.57	85	25
	p721005-1	0.27	46.48	283	716	82.85	0	0
	p721005-2	0.16	53.92	199	627	82.14	0	0
	p721005-3	0.06	51.76	77	631	88.59	0	0
11021	11021_chr	5.45	57.46	5386	893	89.67	85	25
	p11021-1	0.24	51.69	271	682	82.43	0	0
	p11021-2	0.17	53.48	217	605	81.85	0	0

Additionally, the relatively conserved genomic sequences and structures among the 14 *K. pneumoniae* strains were also indicated. Average nucleotide identity (ANI) analysis revealed more than 99% identity among the 14 *K. pneumoniae* strains; no extensive translocations, duplications, or inversions were found in the *K. pneumoniae* genomes except for strains 11492 and 11454, each containing a large inverted fragment in the genome (Figure S2).

### Seven known and eight novel methylation motifs and corresponding **MTases** in **K. pneumoniae** strains

A total of 13 6mA and two 5mC methylation motifs were identified in the 14 *K. pneumoniae* strains (Table 2, Table 3, Table 4) by SMRT and bisulfite sequencing techniques (Tables S2 and S5), including seven known and eight novel methylation motifs (Table 5, Table S6). It is worth noting that G**A**TC and C**C**WGG motifs were shared by all strains. The other motifs were shared by at most two *K. pneumoniae* strains (Table 2, Table 3, Table 4). Further analyses indicated the relationships between the motifs and stain types (serotype, MLST, and CG). The MT**C**GAK (where M = A or C, K = G or T) motif was present in NTUH-K2044 and 11492 belonging to the K1 serotype and ST23-CG23, the most common types of hypervirulent *K. pneumoniae* strains. The CC**A**YN_7_TTYG (where Y = C or T, N = A or C or G or T) motif was shared by two strains (11311 and 23) of hypervirulent serotype K57 and ST412. The CC**A**GN_7_RTTC (where N = A or C or G or T, R = G or A) motif was present in strains 11305 and 13190 belonging to MDR CG147.

**Table 2:** **The three types of modification patterns oftheGATC motif in the 14 *K. pneumoniae* strains**

Strain name	**Motif**	**No. of motifs**	**Methylated motif (%)**	**Hemi-methylated motif (%)**	**Un-methylated motif (%)**
NTUH-K2044	G**A**TC	30,727	30,151 (98.13%)	551 (1.79%)	25 (0.08%)
11492	G**A**TC	30,316	30,271 (99.85%)	31 (0.10%)	14 (0.05%)
11420	G**A**TC	31,847	26,835 (84.26%)	4669 (14.66%)	343 (1.08%)
11454	G**A**TC	30,278	28,046 (92.63%)	2142 (7.07%)	90 (0.30%)
12208	G**A**TC	31,512	30,695 (97.41%)	790 (2.51%)	27 (0.08%)
11311	G**A**TC	30,623	29,520 (96.4%)	1076 (3.51%)	27 (0.09%)
23	G**A**TC	31,275	26,523 (84.81%)	4423 (14.14%)	329 (1.05%)
11305	G**A**TC	30,369	29,222 (96.22%)	1122 (3.69%)	25 (0.09%)
N201205880	G**A**TC	33,171	28,007 (84.43%)	4852 (14.63%)	312 (0.94%)
309074	G**A**TC	31,208	30,384 (97.36%)	782 (2.51%)	42 (0.13%)
13190	G**A**TC	31,419	28,766 (91.56%)	2515 (8.00%)	138 (0.44%)
283747	G**A**TC	31,335	30,156 (96.24%)	1126 (3.59%)	53 (0.17%)
721005	G**A**TC	32,242	31,521 (97.76%)	687 (2.13%)	34 (0.11%)
11021	G**A**TC	32,080	27,907 (86.99%)	3968 (12.37%)	205 (0.64%)

*Note*: The methylated adenine in the motif is shown in bold; the underlined letter represents the thymine pairing with the methylated adenine on the complementary strand. The number of motifs include the ones on the plus and minus strands of chromosomes and plasmids.

**Table 3:** **Modification patterns of the 12 motifs with6mA in the 14 *K. pneumoniae* strains**

Strain name	**Motif**	**No. of motifs**	**Methylated motif (%)**	**Hemi-methylated motif (%)**	**Un-methylated motif (%)**
NTUH-K2044	GR**A**CRAC*	2104	2060 (97.91%)	/	44 (2.09%)
11420	RT**A**CN_5_GGC*	1275	1156 (90.67%)	/	119 (9.33%)
	TTC**A**N_7_TTC*	853	598 (70.11%)	231 (27.08%)	24 (2.81%)
11454	**A**GCN_5_CTTC	991	930 (93.84%)	61 (6.16%)	/
12208	AGGA**A**G*	2864	2848 (99.30%)	/	16 (0.7%)
11311^#^	CC**A**YN_7_TTYG*	667	608 (91.15%)	58 (8.70%)	1 (0.15%)
23^#^	CC**A**YN_7_TTYG*	687	534 (77.73%)	145 (21.11%)	8 (1.16%)
11305^	CC**A**GN_7_RTTC	342	325 (95.03%)	17 (4.97%)	/
N201205880	CC**A**N_7_TCAC*	543	433 (79.75%)	102 (18.78%)	8 (1.47%)
309074	CT**A**N_5_GTAA	165	164 (99.39%)	1 (0.61%)	/
	C**A**GN_6_TCAA*	439	413 (94.08%)	26 (5.92%)	/
	C**A**TCN_6_TTYG	634	589 (92.90%)	45 (7.10%)	/
13190^	CC**A**GN_7_RTTC	379	338 (89.19%)	40 (10.55%)	1 (0.26%)
	GGC**A**N_8_TCG	1065	874 (82.06%)	184 (17.28%)	7 (0.66%)

*Note*: The methylated adenine in each motif is shown in bold; the underlined letter represents the thymine pairing with the methylated adenine on the complementary strand. Degenerate bases used in our recognition sequences are as follows: R = G or A, Y = C or T, M = A or C, K = G or T, S = G or C, W = A or T, B = not A (C or G or T), D = not C (A or G or T), H = not G (A or C or T), V = not T (A or C or G), N = A or C or G or T. The number of motifs include the ones on the plus and minus strands of chromosomes and plasimds. *, newly reported motifs. ^#^, two strains (11311 and 23) possess motif CC**A**YN_7_TTYG. ^, two strains (11305 and 13190) possess motif CC**A**GN_7_RTTC.

**Table 4:** **Modification patterns of the two motifs with 5mC in the 14 *K. pneumoniae* strains**

Strain name	**Motif**	**No. of motifs**	**Methylated motif (%)**	**Hemi-methylated motif (%)**	**Un-methylated motif (%)**
NTUH-K2044	C**C**WGG	19,530	16,117 (82.52%)	1756 (8.99%)	1657 (8.49%)
	MT**C**GAK*	5410	2339 (43.23%)	270 (4.99%)	2801 (51.78%)
11492	C**C**WGG	19,284	19,015 (98.61%)	86 (0.45%)	183 (0.94%)
	MT**C**GAK*	5386	2368 (43.97%)	258 (4.79%)	2760 (51.24%)
11420	C**C**WGG	20,175	18,828 (93.32%)	839 (4.16%)	508 (2.52%)
11454	C**C**WGG	19,314	14,983 (77.58%)	2367 (12.26%)	1964 (10.16%)
12208	C**C**WGG	20,108	19,874 (98.84%)	35(0.17%)	199 (0.99%)
11311	C**C**WGG	19,668	19,126 (97.24%)	299 (1.52%)	243 (1.24%)
23	C**C**WGG	19,941	18,668 (93.62%)	963 (4.83%)	310 (1.55%)
11305	C**C**WGG	19,460	18,964 (97.45%)	261 (1.34%)	235 (1.21%)
N201205880	C**C**WGG	21,115	20,867 (98.83%)	38 (0.18%)	210 (0.99%)
309074	C**C**WGG	19,966	17,464 (87.47%)	1460 (7.31%)	1042 (5.22%)
13190	C**C**WGG	20,060	19,149 (95.46%)	694 (3.46%)	217 (1.08%)
283747	C**C**WGG	20,156	19,141 (94.96%)	574 (2.85%)	441 (2.19%)
721005	C**C**WGG	20,832	20,470 (98.26%)	145 (0.70%)	217 (1.04%)
11021	C**C**WGG	20,736	18,352 (88.50%)	1358 (6.55%)	1026 (4.95%)

*Note*: The methylated cytosine in the motif is shown in bold; the underlined letter represents the guanine pairing with the methylated cytosine on the complementary strand. Degenerate bases used in our recognition sequences are listed as follows: R = G or A, Y = C or T, M = A or C, K = G or T, S = G or C, W = A or T, B = not A (C or G or T), D = not C (A or G or T), H = not G (A or C or T), V = not T (A or C or G), N = A or C or G or T. The number of motifs include the ones on the plus and minus strands of chromosomes and plasimds. *, newly reported motifs.

**Table 5:** **The 15 methylation motifs and corresponding DNA MTases in the 14 *K. pneumoniae* strains**

Motif	**Modification**	**R-M/orphan**	**MTase**	**Comment**
G**A**TC	6mA	Orphan	Dam	Known
RT**A**CN_5_GGC	6mA	R-M system Type I	KamA	New
TTC**A**N_7_TTC	6mA	R-M system Type I	KamB	New
GR**A**CRAC	6mA	R-M system Type II	KamC	New
**A**GCN_5_CTTC	6mA	R-M system Type I	M.KpnGH01II	Known
AGGA**A**G	6mA	R-M system Type II	KamD	New
CC**A**YN_7_TTYG	6mA	R-M system Type I	KamE	New
CC**A**N_7_TCAC	6mA	R-M system Type I	KamF	New
CC**A**GN_7_RTTC	6mA	R-M system Type I	M.KpnAATI	Known
CT**A**N_5_GTAA	6mA	R-M system Type I	M.Kpn35657I	Known
C**A**GN_6_TCAA	6mA	R-M system Type I	KamG	New
C**A**TCN_6_TTYG	6mA	R-M system Type I	M.Kpn39795II	Known
GGC**A**N_8_TCG	6mA	R-M system Type I	M.KpnAATIV	Known
C**C**WGG	5mC	Orphan	Dcm	Known
MT**C**GAK	5mC	R-M system Type II	KcmA	New

*Note*: The methylated nucleotide in each motif is shown in bold; the underlined letter represents the nucleotide pairing with the methylated nucleotide on the complementary strand. Degenerate bases used in the recognition sequences are as follows: R = G or A, Y = C or T, M = A or C, K = G or T, S = G or C, W = A or T, B = not A (C or G or T), D = not C (A or G or T), H = not G (A or C or T), V = not T (A or C or G), N = A or C or G or T. The MTase prediction was based on the sequence alignment with REBASE database (http://rebase.neb.com/rebase/rebase.html). The predicted MTases were further classified as Type I, Type II, or orphan MTases according to the annotation information. MTase, methyltransferase; R-M, restriction-modification.

Modification analysis indicated that not all motif sites were fully methylated (methylated on both strands, Table 2, Table 3, Table 4). A minority of motif sites (< 30%) were detected as being hemi-methylated (methylated on one strand only) or un-methylated within the *K. pneumoniae* genomes. The only exception was the MT**C**GAK motif in the NTUH-K2044 and 11492 genomes, in which over half of the sites were hemi-/un-methylated (56.03%–56.77%). Further analysis indicated that the un-methylated MT**C**GAK motif tended to be preceded by a guanine (G) (Figure S3, Table S7).

To search for the respective MTases, we first predicted 22 MTase genes [24]. Among them, seven genes encode MTases which as well as their corresponding motifs had been verified in previous studies (Table 5). To determine the MTases responsible for the eight newly detected methylation motifs, we performed restriction digestion and SMRT/bisulfite sequencing using plasmids containing the predicted MTase genes in MTase-free *E. coli* ER2796. Crossover validation identified the corresponding eight MTases that could specifically recognize and methylate the respective eight novel motifs (Table 5, Tables S6 and S8; Figures S4–S6).

**Figure 2:**
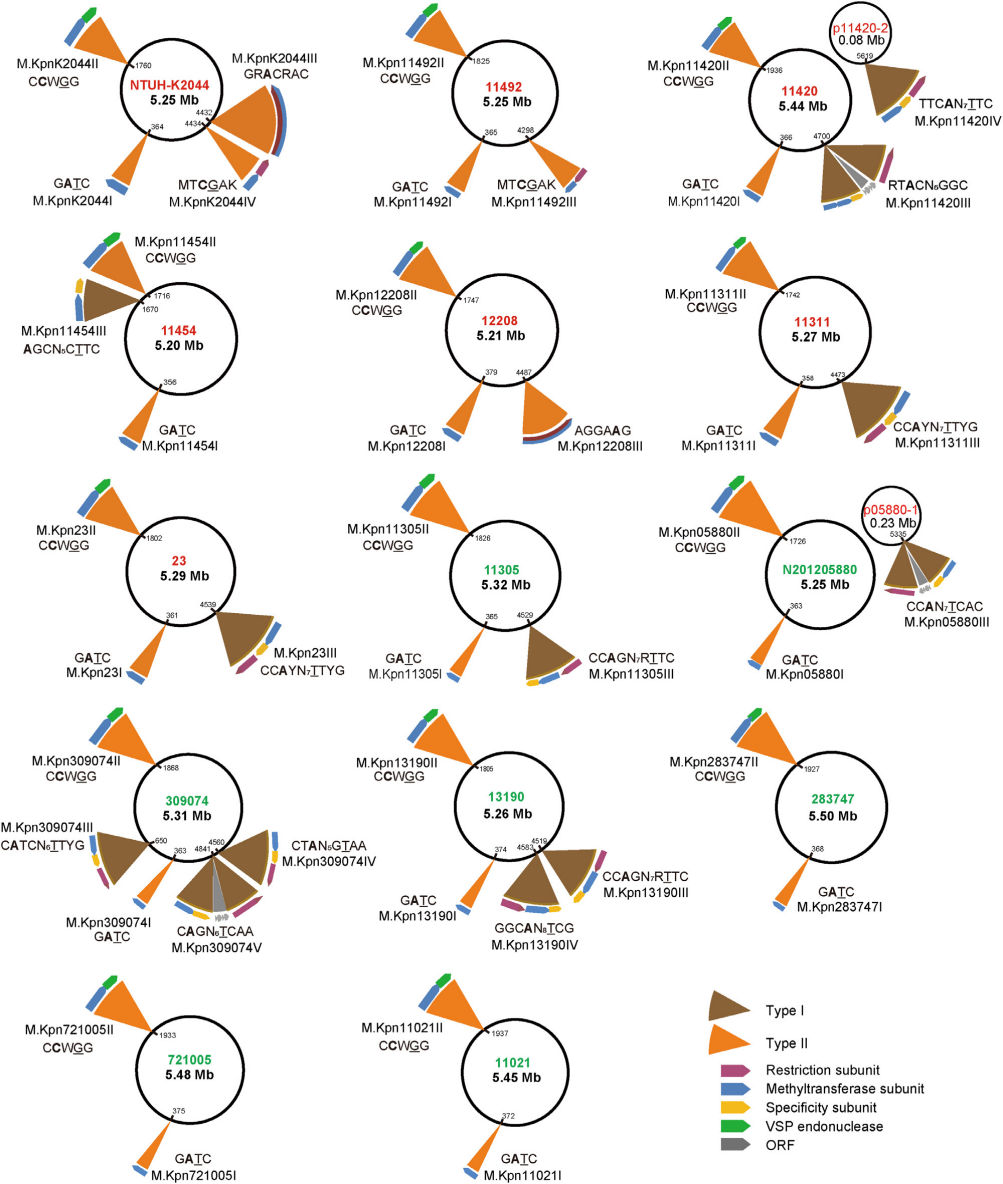
**The 15 MTase genes and corresponding methylationmotifs for the 14*K. pneumoniae*strains** Hypervirulent and low-virulent strains are labeled in red and green, respectively. The large and small circles represent chromosome and plasmid genomes, respectively. ORF, open reading frame.

We further analyzed the distribution of 15 MTase genes in the genomes of the *K. pneumoniae* strains. Thirteen genes were located on chromosomes, and two others were located on plasmids (Figure 2). Additionally, within the 15 identified MTases, there were 10 Type I MTases, three Type II MTases, and two classical orphan MTases (Dam and Dcm). Here, *dam* and *dcm* genes were present in all *K. pneumoniae* strains, and responsible for the methylation of G**A**TC and C**C**WGG motifs, respectively. Among the three newly identified Type II MTases, KamC and KamD were predicted to be Type IIG enzymes, for which endonuclease and methyltransferase activities are encoded by a single gene (Table 5, Table S6).

### Nonrandom distributions of GATC and CCWGG motifs in ***K. pneumoniae*** genomes

Among the 15 methylation motifs, G**A**TC and C**C**WGG had the most extensive distributions in all 14 *K. pneumoniae* strains, each containing ∼ 30,000 G**A**TC and ∼ 20,000 C**C**WGG modified sites (Table 2, Table 4). The distributions of the two motifs in the genomes of *K. pneumoniae* strains were further analyzed, and differential/uneven distributions were observed (Figure 1). Both motifs exhibited some high-density and low-density regions in the genomes, where the genes were clustered into different Clusters of Orthologous Groups (COG) functional categories (Figure S7). Notably, the G**A**TC motif showed the highest distribution density in the *oriC* region (∼ 34 sites/kb) of the 14 *K. pneumoniae* genomes (average density: 5–6 sites/kb) (Figure S8). By contrast, the C**C**WGG motif did not display such enrichment in the *oriC* region.

**Figure 3:**
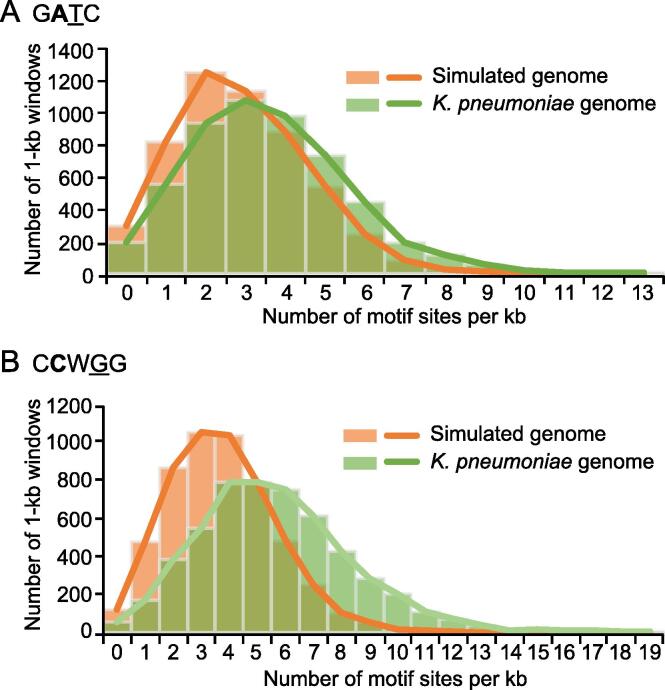
**Density distribution of the GATC/CCWGG motifsin the *K. pneumoniae* and randomly generated genomes** Green histograms show the density distributions of G**A**TC (**A**) and C**C**WGG (**B**) in the *K. pneumoniae* genome, which follow Poisson distributions with λ = 5.64/3.62. Orange histograms show the density distributions of G**A**TC (A) and C**C**WGG (B) motifs in the randomly generated genome, which also follow Poisson distributions with λ = 3.73/2.89 (total number of G**A**TC/C**C**WGG motifs × 1000 per genome size).

We then compared the density distributions of the two motifs in the 14 *K. pneumoniae* genomes and the simulated genome with the same base composition (Figure 3, Figures S9 and S10). The results revealed that their distribution densities in the *K. pneumoniae* genomes (1-kb consecutive window) were higher than those in the simulated genome, indicating a high-density/nonrandom distribution for these two motifs in the *K. pneumoniae* genome. To explore the underlying causes for their high-density distributions, we investigated the impact of selection pressure on these two motifs by calculating the ratio of nonsynonymous substitutions (Ka) to synonymous substitutions (Ks) [25] of the corresponding fragments in gene regions (GRs), and more than 90% of the two motifs were located in GRs (Figure S11). We observed that the amino acid (AA) codons with two motifs (two AA codons for the G**A**TC motif, two or three AA codons for the C**C**WGG motif) were under strong negative/purifying selection with Ka/Ks of ∼ 0.09/0.09, compared with a ratio of 0.39/0.54 for the scramble (control) sequences in GRs.

### Differential distribution patterns of methylated, hemi-methylated, and un-methylated GATC and CCWGG motifs in ***K. pneumoniae*** genomes

We identified three methylation patterns (methylated, hemi-methylated, and un-methylated) for the G**A**TC and C**C**WGG motifs. Most G**A**TC (84.26%–99.85%) and C**C**WGG (77.58%–98.84%) sites were found to be methylated (Tables 2 and 4), while only a small percentage of motif sites were hemi-methylated (< 15%) or un-methylated (< 10%) in the *K. pneumoniae* genomes. Further analysis demonstrated that the ratio of hemi-methylated G**A**TC motifs (∼ 6.48%) was much higher than that of un-methylated G**A**TC motifs (∼ 0.38%), while hemi-methylated (∼ 3.60%) and un-methylated (∼ 2.94%) C**C**WGG motifs accounted for similar proportions.

**Figure 4:**
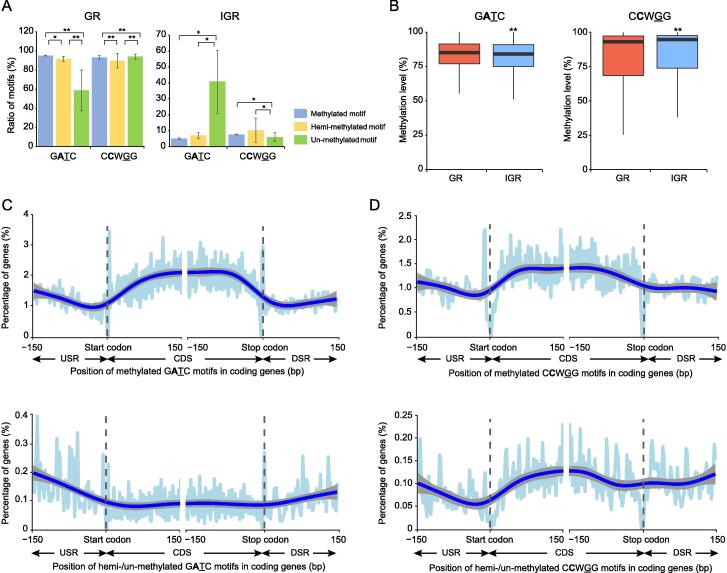
**Distribution of GATC/CCWGG motifs with differentmethylation patterns in GRs and IGRs** **A.** Bar plots showing the ratios of G**A**TC/C**C**WGG motifs with different methylation patterns in GRs and IGRs in the 14 *K. pneumoniae* strains. Blue, yellow, and green bars indicate the ratios of methylated, hemi-methylated, and un-methylated motifs in GRs and IGRs, respectively. **B.** Box plots showing the methylation levels of G**A**TC/C**C**WGG motifs in GRs and IGRs (red and blue boxes, respectively). **C.** Frequency distribution of the methylated (top) and hemi-/un-methylated (bottom) G**A**TC motifs in GRs and IGRs in the 14 *K. pneumoniae* strains. **D.** Frequency distribution of the methylated (top) and hemi-/un-methylated (bottom) C**C**WGG motifs in GRs and IGRs in the 14 *K. pneumoniae* strains. The dotted gray lines represent the positions of the start and stop codons. USR, 5′ upstream region; CDS, coding sequence; DSR, 3′ downstream region; GR, gene region; IGR, intergenic region. *, *P* < 0.05; **, *P* < 0.01.

We then investigated the distribution ratios of methylated, hemi-methylated, and un-methylated G**A**TC and C**C**WGG motifs in GRs and IGRs (Tables S9 and S10). The hemi-/un-methylated G**A**TC motifs tended to be located in IGRs, since their ratios in IGRs were significantly higher than that of the methylated G**A**TC motifs (7.78% for hemi-methylated, 41.79 for un-methylated, 5.14% for methylated; *P* < 0.01, Figure 4A, Table S9). Analysis of the ‘fraction of methylated reads’ (FRAC value) for the motifs in GRs/IGRs also supported the aforementioned finding that hemi-/un-methylated G**A**TC motifs tended to be located in the IGRs (Figure 4B).

Analysis of the G**A**TC motif density in the 5′ upstream region (USR), the coding sequence (CDS), and the 3′ downstream region (DSR) also supported the aforementioned conclusion: the methylated motifs displayed a higher density in GRs, while the hemi-/un-methylated motifs were more abundant in IGRs (both 5′ USR and 3′ DSR; Figure 4C). As for the C**C**WGG motifs, we did not observe higher abundant hemi-/un-methylated sites in IGRs than in GRs (Figure 4D).

We further explored the hemi-/un-methylated G**A**TC sites shared in the 5′ USRs of 14 *K. pneumoniae* strains, and 13 hemi-/un-methylated G**A**TC sites corresponding to 11 genes were observed (Table S11). We also detected 12 high-density hemi-/un-methylated G**A**TC clusters (no less than three consecutive hemi-/un-methylated motifs in at least two strains) in the 5′ USRs (Table S12).

We then analyzed the sequence conservation of the DNA fragments (20 nt on both sides) containing hemi-/un-methylated or methylated motifs to determine the causes leading to their differential distributions in IGRs. The results showed that the fragments with hemi-/un-methylated G**A**TC motifs (44 nt) exhibited higher conservation than those with methylated G**A**TC motifs in IGRs (Figure S12).

### Methylation kinetic analysis revels different re-methylation rates for GATC and CCWGG motifs during the growth cycle

**Figure 5:**
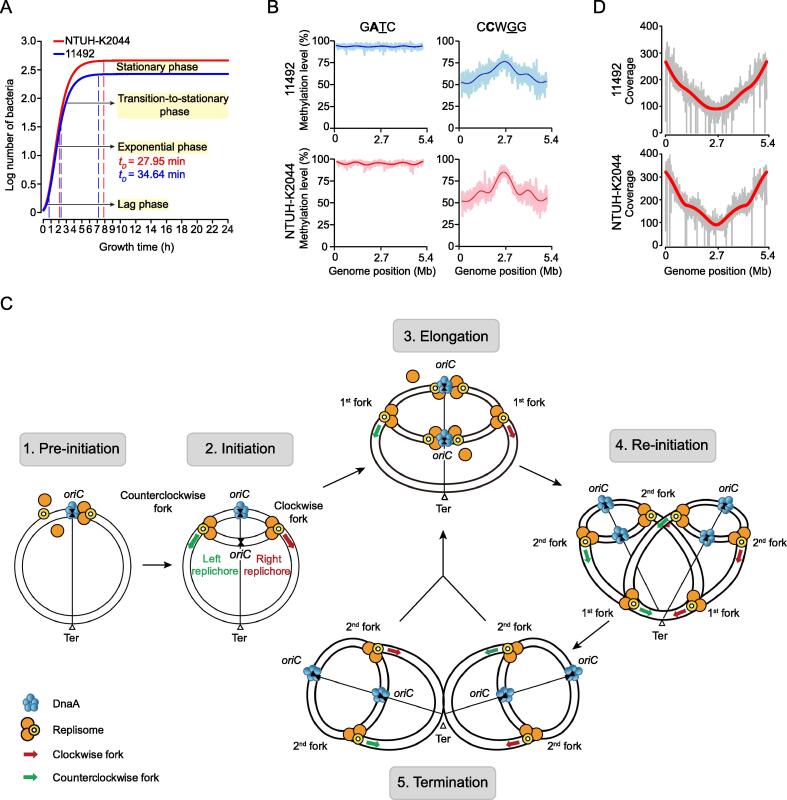
**Dynamic methylation analysis of GATC and CCWGGmotifs in NTUH-K2044 and 11492** **A.** Growth curves of *K. pneumoniae* NTUH-K2044 (red) and 11492 (blue). The X-axis represents the growth time, including lag phase, exponential phase, transition-to-stationary phase, and stationary phase. The Y-axis represents the logarithms of normalized OD values. At 1, 4, and 24 h, aliquots of bacterial cultures were collected and sequenced using PacBio and Illumina platforms. The *t_D_* values of the two strains are also labeled in the plot. **B.** Genome-wide methylation level *versus* genome position for the two motifs (G**A**TC and C**C**WGG) at the exponential phase. The bold fitting lines approximate the average methylation levels across the genomes (5-kb window size). **C.** Schematic diagram showing the process of DNA replication in the exponential phase. 1) The origin replication complex binds to the *oriC* region. 2) The first round of replication is initiated when the first replication complex binds the *oriC* region. 3) The second round of replication begins before completion of the first round of replication. 4) Six replication forks are generated in one bacterium. 5) The first round of replication completes, followed by a new cell replication cycle. **D.** Fitting of the genomic coverages with the mathematical model of replication. The genomic coverage plots of 11492 (upper) and NTUH-K2044 (lower) strains are shown in gray, and the mathematically fitting curves are shown in red. *t_D_*, doubling time; *oriC*, replication origin.

Since there are no demethylases in bacteria, *in vivo* methylation kinetics characterisation is based on the dynamic equilibrium between replication-mediated passive demethylation and MTase-catalyzed re-methylation [18]. To explore the features of methylation kinetics of G**A**TC and C**C**WGG motifs during the growth cycle, we first characterized the methylomes of the NTUH-K2044 and 11492 strains at the exponential phase (at 1 h), the transition-to-stationary phase (4 h), and the stationary phase (at 24 h) (Figure 5A; Tables S13 and S14). By comprehensively analyzing the genome-wide sequencing coverage and the fraction of methylated reads (methylated read ratio/FRAC value), G**A**TC and C**C**WGG motifs were found to exhibit distinct kinetic features during the growth cycle (Figure 5B, Figure S13). Regarding the G**A**TC motif, the methylated read ratios were more than 90% throughout the genomes in all three phases (Figure 5B), although the sequencing coverage varied between genomes during the growth cycle (Figure S13A). This indicates that the G**A**TC motif may be re-methylated in a very short time after passive demethylation caused by replication (*i.e.*, the re-methylation rate was almost identical to the passive demethylation rate).

Compared to the G**A**TC motif, the C**C**WGG motif showed a much slower re-methylation. First, in the exponential phase (at 1 h), the methylated read ratio of C**C**WGG motifs in the *oriC* regions (55.66% ± 18.54%) was much lower than that in the replication termination (*Ter*) regions (82.63% ± 12.71%; Figure S13B). Secondly, the average methylated read ratio in the transition-to-stationary phase (54.50% ± 27.14%, at 4 h) was close to that of the exponential phase (60.50% ± 17.71%, at 1 h), but not close to that of the stationary phase (80.28% ± 19.05%, at 24 h; Figure S13B). These findings suggest a much slower re-methylation, compared to the replication-mediated passive demethylation rate for the C**C**WGG motif, which resulted in the aforementioned differences in methylated read ratios.

To quantify the re-methylation time per motif in the exponential phase, we first investigated the replication kinetics of the two aforementioned *K. pneumoniae* strains. Figure S13 shows that the genome sequencing coverage in the *oriC* regions was more than twice as much as that in the *Ter* regions (2.93- and 3.02-fold for the 11492 and NTUH-K2044 strains, respectively), indicating that the next round of replication was re-initiated before replication termination in the exponential phase. We then constructed a replication model (Figure 5C) by fitting genome coverage data, and obtained the ratio (*t_D_*/*t_R_*) of doubling time (*t_D_*) to replication termination time (*t_R_*) as described in the Materials and methods. The *t_D_* in the exponential phase also reflects the re-initiation time. Interestingly, we obtained the same *t_D_*/*t_R_* values (0.59) for the two *K. pneumoniae* strains (Figure 5D), suggesting a similar regulatory mechanism for the replication cycle, as expected for *K. pneumoniae* strains. Two *t*_*D*_ values were then calculated by fitting growth curves (∼ 34.64 min for strain 11492; ∼ 27.95 min for NTUH-K2044; Figure 5A). We could therefore infer *t*_*R*_ from the *t_D_*/*t_R_* (∼ 58.71 min and ∼ 47.36 min for strains 11492 and NTUH-K2044, respectively).

We further obtained the re-methylation time per motif (Table 6) by simulating the dynamic processes of passive demethylation and re-methylation in the exponential phase based on five parameters: the methylation read fraction of each motif (*M*_(_*_x_*_)_), the initial methylation read fraction of each motif ($M_{\left(x\right)}^{0}$), *t*_*D*_, *t*_*R*_, and the distribution density of the first replication forks ($P_{\left(x_{1}\right)}$) (see Materials and methods for details).

**Table 6:** **Averagere-methylation time per motif intheNTUH-K2044 and 11492 strains**

**Motif sequence**	**No. of motifs (%)**	**Re-methylation time (min)**
G**A**TC	59,424 (100%)	3.52 ± 0.54
SG**A**TCS (fast)	17,041 (28.7%)	3.41 ± 0.54**
WG**A**TCW (slow)	9803 (16.5%)	3.78 ± 0.54***
C**C**WGG	35,516 (100%)	9.23 ± 4.93
SC**C**WGGS (slow)	13,573 (38.2%)	9.65 ± 4.88***
WC**C**WGGW (fast)	5800 (16.3%)	8.27 ± 4.48***
MT**C**GAK	10,467 (100%)	4.55 ± 0.54
GR**A**CRAC	2002 (100%)	3.46 ± 0.45

*Note*: Degenerate bases used in our recognition sequences are as follows: R = G or A, Y = C or T, M = A or C, K = G or T, S = G or C, W = A or T, B = not A (C or G or T), D = not C (A or G or T), H = not G (A or C or T), V = not T (A or C or G), N = A or C or G or T. Values for re-methylation time are shown as mean ± SD; ** and *** means that the re-methylation time of the motifs with certain flanking sequences show the significant difference (**, *P* < 0.01; ***, *P* < 0.001) comparing with the average re-methylation time of total motifs.

In general, the mean re-methylation time of 6mA was shorter (3.52 min and 3.46 min for G**A**TC and GR**A**CRAC motifs, respectively) than that of 5mC (9.23 min and 4.55 min for C**C**WGG and MT**C**GAK motifs, respectively) in the exponential phase (Table 6). Regarding specific motifs, the re-methylation time in the NTUH-K2044 strain was slightly shorter than that in the 11492 strain (Figure S14). In addition, the flanking bases could influence the re-methylation rates of the G**A**TC and C**C**WGG motifs; when the flanking bases were C/G rather than T/A, the G**A**TC motif exhibited a faster re-methylation, which was completely reversed for the C**C**WGG motif (Table 6; Figure S15).

### The re-methylation of GATC motifs in the intergenic and ***oriC*** regions is slow at the exponential phase

**Figure 6:**
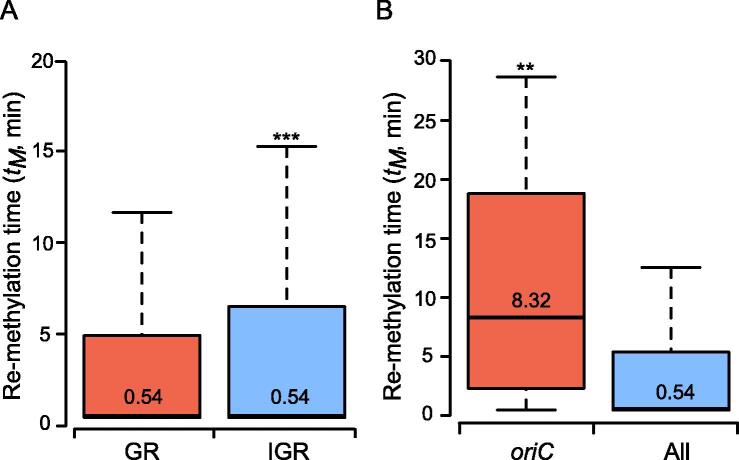
**Comparison of re-methylation time of GATC motifs in differentgenome regions** **A.** Box plot showing the re-methylation time of G**A**TC motifs in GRs and IGRs. **B.** Box plot showing the re-methylation time of G**A**TC motifs in *oriC* and all regions. The value in each box represents the midian. **, *P* < 0.01; ***, *P* < 0.001. *t_M_*, mean re-methylation time.

To investigate the role of G**A**TC motifs in transcriptional regulation in *K. pneumoniae* strains, we analyzed the re-methylation rates of the motifs in IGRs (including 5′ USRs and 3′ DSRs) at the exponential phase (Figure 6A). The results indicated slower re-methylation of G**A**TC motifs in IGRs (3.94 ± 5.82 min) than in GRs (3.50 ± 5.10 min). Since most 5′ USRs in bacteria overlap with the promoter regions, and are involved in transcriptional regulation [26], we further explored the COG functional categories of the top 5% of genes with the slowest re-methylation sites (> 7.04 min/motif) in 5′ USRs (Table S15). Four enriched functional categories (“cell cycle control, cell division, chromosome partitioning”, “carbohydrate transport and metabolism”, “intracellular trafficking, secretion, and vesicular transport”, and "translation, ribosomal structure and biogenesis") were observed (Table S15), of which at least 5% of genes contained slow re-methylation sites (> 7.04 min/motif) in 5′ USRs. Among them, the “carbohydrate transport and metabolism” functional category accounted for the most genes (22) with slow re-methylation in 5′ USRs, including three hemi-/un-methylated motifs shared in the 14 *K. pneumoniae* strains, as well as one hemi-/un-methylated motif cluster.

To further explore the role of the G**A**TC motifs in regulating replication in *K. pneumoniae* strains, we analyzed the re-methylation rates of the G**A**TC motifs in *oriC* regions (Figure 6B). The G**A**TC motifs in *oriC* regions had the highest distribution density (∼ 34 sites/kb) (Figure S8) and slower re-methylation rates (10.35 ± 8.69 min), compared to the average re-methylation rate of all genomic regions. Among the 14 G**A**TC motifs in *oriC* regions, nine and eight motifs with slow re-methylation (> 7.04 min/motif) were enriched in the upstream sequence of the AT-rich region in 11492 and NTUH-K2044 strains, respectively (Figure S16).

## Discussion

In this study, we precisely characterized the methylomes of 14 *K. pneumoniae* strains differing in serotypes, MLSTs, CGs, viscosity/virulence, and drug resistance using SMRT/bisulfite sequencing, and identified 15 DNA methylation motifs (eight of which were novel) corresponding to 13 R-M system MTases and two orphan MTases. Two motifs (G**A**TC and C**C**WGG) and their respective orphan MTases (Dam and Dcm) appeared to be the most important since they were present in all *K. pneumoniae* strains, with more extensive distributions in genomes than other motifs (Tables 2 and 4). This feature was also reported previously for almost all members of the Enterobacteriaceae family [21], [24]. Functional analysis in previous reports indicated that these two motifs, especially G**A**TC, may perform multiple functions including transcriptional regulation, cell cycle control, and mismatch repair in *E. coli* and *S. enterica* [15], [16], [17], [19], [20], [21]. We also demonstrated their high-density distributions in the *K. pneumoniae* genome relative to the simulated genome (Figure 3, Figures S9 and S10), presumably due to purifying selection (Figure S11), leading to their evolutionary conservation (Figure S12), as reported for other bacterial genomes [27]. This well-conserved feature also implies an essential role of these two motifs in *K. pneumoniae*.

The hemi-/un-methylated G**A**TC motifs tended to be localised in IGRs, including 5′ USRs and 3′ DSRs (Figure 4A and C). Since promoters in *K. pneumoniae* were also predicted to be located throughout the 5′ USRs (Figure S17), these hemi-/un-methylated G**A**TC motifs in 5′ USRs might be protected from methylation by competitively binding certain regulators to the promoter regions [17], [19]. In *E. coli* strains, this feature facilitates the epigenetic regulation of downstream gene expression [28]. The status of these motifs suggests similar epigenetic mechanisms in *K. pneumoniae* strains, which might be a consequence of selection during long-term evolution (Figure S11), since our findings suggest that the genomic fragments with hemi-/un-methylated G**A**TC motifs in IGRs have higher sequence conservation (Figure S12).

Importantly, by establishing a mathematical model to simulate the dynamic processes of passive demethylation and re-methylation for each motif in the exponential phase, we derived the re-methylation time for each motif throughout the whole genome (Table 6; Figure S14). Our studies revealed that the motifs at different genomic locations display different re-methylation rates. We could reasonably infer that the slower re-methylation in some sites/regions might also be due to the competitive binding of certain proteins to prevent methylation [17], [19]. Thus, the slow re-methylation could precisely reflect the methylation-mediated epigenetic regulation at these sites/regions *in vivo*.

There are two types of methylation-mediated epigenetic regulation, transcription and replication, and both were examined by methylation kinetic analysis in our study. Firstly, the transcriptional regulation analysis indicated that the G**A**TC motifs in IGRs present slower re-methylation than those in GRs (Figure 6). This is consistent with the distribution characteristics of the G**A**TC motifs in IGRs (*i.e.*, lower methylated read ratios and more hemi-/un-methylated motifs; Figure 4A and B). As described above, most IGRs in bacteria overlap with the promoter regions and participate in transcriptional regulation [26]. Thus, the promoter regions with slow re-methylation motifs should be the locations where the G**A**TC motifs with hemi-/un-methylated status function as transcription regulators in *K. pneumoniae* strains. Similarly, this should also be a consequence of the competitive binding between Dam and certain transcription regulatory proteins (such as OxyR) to these sites/regions [15], [28], [29], which is analogous to the epigenetic transcriptional regulation mediated by competition between DNA MTases and CCCTC-binding factor (CTCF) in CpG islands of eukaryotic cells [30]. Secondly, replication regulation analysis identified 7–8 slow G**A**TC motifs enriched in the upstream sequence of the fourth DnaA binding site (DnaA box) and the adjacent AT-rich region (Figure S16). SeqA has been shown to bind preferentially to these motifs in order to lower re-methylation rates [31] and prevent the initiation cascade for chromosome replication [17], [31]. Therefore, re-methylation of these motifs in the fourth DnaA box and AT-rich regions likely represents the main rate-limiting steps for triggering DNA replication initiation in *K. pneumoniae* strains. We also identified numerous promoter regions with slower re-methylation motifs in *K. pneumoniae* strains (Figure 6); therefore, it is reasonable to speculate that epigenetic regulation in bacteria is complex rather than simple, as previously believed.

Compared with the G**A**TC motif, the C**C**WGG motif has different distribution characteristics in GRs and IGRs (Figure 4A and B). We therefore performed COG functional analysis of genes with hemi-/un-methylated C**C**WGG sites in IGRs, since previous studies demonstrated the epigenetic regulation of hemi-/un-methylated sites in IGRs in bacteria [32]. Our findings showed that the top three enriched functional categories for genes with hemi-/un-methylated C**C**WGG sites in IGRs were “replication, recombination and repair”, “cell motility”, and “coenzyme transport and metabolism” (Figure S18A), suggesting the possible epigenetic regulation of C**C**WGG methylation in *K. pneumoniae*. However, the analysis implied totally different COG categories for genes with hemi-/un-methylated G**A**TC sites in IGRs (Figure S18B), suggesting differences in epigenetic regulation between the two main methylated sites in *K. pneumoniae*.

We also explored the methylation kinetic features of C**C**WGG motifs during the growth cycle. A total of 1961 C**C**WGG sites were differentially methylated during the growth cycle: 1954 sites that were hemi-/un-methylated in the exponential phase became methylated in the stationary phase, and seven sites that were un-methylated in the exponential phase became hemi-methylated in the stationary phase. No sites that were methylated in the exponential phase became hemi-/un-methylated in the stationary phase. Such dynamic changes in methylation status might be due to active and inactive replication in exponential and stationary phases [13]. Active replication at the exponential phase may result in more hemi-/un-methylated sites in the genome due to replication-mediated passive demethylation; inactive replication at the stationary phase may lead to more methylated sites in the genome due to almost complete MTase-catalyzed methylation. Incidentally, seven hemi-methylated C**C**WGG sites in the stationary phase are located in genes encoding four small subunit ribosomal RNAs, two mobile element proteins, and one possible transcription regulator (Table S16), which may be necessary for the survival of bacteria [33].

Importantly, eight novel MTases and related motifs were detected, including five Type I and three Type II MTases (Table 5, Tables S6 and S8). These novel MTases and cognate restriction endonucleases (REases) form R-M systems, which are known to protect bacterial cells by cleaving foreign phage DNA [9], [10], [11], [12], [13], [14]. We subsequently investigated the hemi-/un-methylated sites in IGRs, since previous studies demonstrated epigenetic regulation of hemi-/un-methylated sites in IGRs of some bacteria [32]. Among the eight novel methylation mofits, the MT**C**GAK motifs were shared by the NTUH-K2044 and 11492 strains, which possessed the most hemi-/un-methylated sites (56.77% and 56.03% for NTUH-K2044 and 11492 strains, respectively) among the strains investigated (Table 4). More than 60% of un-methylated MT**C**GAK motifs were shared by the two strains (Table S17), which were mainly enriched in the “transcription”, “inorganic ion transport and metabolism”, and ”replication, recombination and repair” COG categories (Figure S19). The CC**A**YN_7_TTYG motifs were also present in two strains (23 and 11311) (Table 3). Three hemi-methylated CC**A**YN_7_TTYG motifs were shared by these two strains, associated with *ascB*, *deoC*, and *fecA* genes, respectively (Table S18). These hemi-/un-methylated MT**C**GAK and CC**A**YN_7_TTYG motifs in IGRs were shared by two strains, and they may contribute to the epigenetic regulation of related gene expression. In addition, we also performed COG analysis on the other six newly identified methylation motifs, which were only detected in one of the *K. pneumoniae* strains (Figure S19). These hemi-/un-methylated sites in only one *K. pneumoniae* strain may be derived from replication-mediated passive demethylation [34], which represents a limitation.

Most previous studies on MTase kinetics focused on *in vitro* analysis [35], [36], [37]. Several analyses on *in vivo* methylation kinetics have been reported, including one publication on bacteria, but have not explored single-motif methylation kinetics [38], [39], [40]. Our current study is the first to characterize the *in vivo* methylation kinetics at single motif resolution throughout the whole genome. The findings provide valuable resources to better understand epigenetic regulation in this and other bacterial species.

## Materials and methods

### Strain information, growth curves, and phenotypic characterisation

Details of the 14 *K. pneumoniae* strains used in this study are included in Table S1. The strains were cultured overnight in Luria-Bertani (LB) medium at 37 °C. A 1 ml sample of overnight culture was transferred to a flask containing 200 ml of LB medium and cultured in a shaker at 200 r/min. The bacterial growth curve was determined by recording OD_600_ values at different time points. This experiment was performed in triplicate.

Drug susceptibility tests were performed by VITEK 2 (bioMérieux, Durham, NC), and drug resistance phenotypes were determined by Clinical and Laboratory Standards Institute (CLSI) standards (https://clsi.org/standards/products/microbiology/documents/m100/). The string test was conducted on *K. pneumoniae* strains, and hypermucoviscosity was defined by viscous strings with a length of more than 5 mm [41].

### Genomic DNA extraction, sequencing, assembly, correction, and annotation

Genomic DNA was extracted using TIANamp Bacteria Genomic DNA Kit (Catalog No. DP302, TIANGEN, Beijing, China). Whole-genome sequencing was performed using a PacBio RS II platform with P6/C4 chemistry (Pacific Biosciences, CA). Each strain was sequenced using 1–2 SMRT cells with genome coverage of more than 50× (Table S2).

*De novo* assembly of the genome was performed using Hierarchical Genome Assembly Process 3 (HGAP3) in the SMRT Portal (v2.2.0; https://smrt-analysis.readthedocs.io/en/latest/SMRT-Pipe-Reference-Guide-v2.2.0/). Gap closing was completed by PBJelly [42]. Based on BLAST results, genome circularisation was finished by manually removing overlapping contig regions.

To correct polymer errors, we re-sequenced strains using Illumina sequencing (Table S3). Paired-end libraries were prepared, and clean reads were obtained after eliminating redundant and low-quality raw reads. Paired reads were extracted and then mapped onto the assembled genome sequences to obtain unique mapped reads using BWA [43], and Pilon (v1.13) was subsequently employed to polish genome sequences using unique mapped reads [44].

Genome sequences were annotated by the Rapid Annotation using Subsystem Technology (RAST) [45]. Unannotated genes were then predicted by alignment in the NCBI non-redundant (NR) database using BLAST (https://blast.ncbi.nlm.nih.gov/Blast.cgi). Protein functions were annotated based on COG, tRNAs were predicted by tRNAscan-SE [46], and rRNAs were predicted by ‘search_for_rnas’ tools in the RAST server (http://RAST.nmpdr.org).

### Genome structure and phylogenetic analysis

ANI and coverage were calculated by ANI on EzBioCloud (http://www.ezbiocloud.net/tools/ani) and online BLAST. Multiple alignments of genomic sequences were performed using Mauve multiple alignment software [23].

Single nucleotide polymorphisms (SNPs) were detected by MUMmer [47] based on the 14 genomes in this study and 62 published genomes (Table S4) using HS11286 as reference. PRANK [48] was used to annotate the protein-coding genes of the 76 *K. pneumoniae* genomes, and Roary was employed to predict 3173 core genes [49]. SNPs (117,142) in core genes were used to construct a phylogenetic tree based on maximum likelihood using FastTree [50], followed by decoration using evolview (v2) [51].

### Genome-wide detection of **6**mA and related motifs using SMRT sequencing data

The SMRT Portal (v2.2.0) was applied to detect genome-wide 6mA modification and related motifs using standard settings in the “RS_Modification_and_Motif_Analysis.1” protocol as previously described (https://smrt-analysis.readthedocs.io/en/latest/SMRT-Pipe-Reference-Guide-v2.2.0/). We subsequently identified the motifs by selecting the top 1000 kinetic hits and submitting a window of ±20 bases around the detected modified base to MEME-ChIP [52], and then compared the results with predicted MTase targeting motif sequences in REBASE [24].

There are three methylation patterns for motifs: methylated, hemi-methylated, and un-methylated. Methylated/hemi-methylated/un-methylated motifs indicate sites with methylated nucleotides on both/one/no strands, respectively.

### Genome-wide detection of 5mC and related motifs using bisulfite sequencing data

The 5mC methylation was detected by bisulfite sequencing (Table S5). Trimmomatic (v0.32) [53] was used to trim adapters and low-quality bases using default parameters. Clean reads were mapped against reference genomes by Bismark (v0.12.2) [54]. We identified motifs by submitting a window of ±20 bases around the detected modified base to MEME-ChIP [52], and then compared the results with predicted MTase targeting motif sequences in REBASE [24].

### MTase cloning and verification

The predicted MTase genes were amplified from bacterial genomic DNA using gene-specific primers (Table S19) and cloned into the plasmid pRRS as previously described [14]. The corresponding methylation-sensitive restriction sites (used to detect the activity of MTases) were included in the 3′-end oligonucleotides. The recombinant plasmids were transformed into the ER2796 bacterial host (not containing known MTase genes), followed by bacterial culture overnight. Plasmids were prepared using QIAprep Spin Miniprep Kit (Catalog No. 27104, QIAGEN). The appropriate restriction enzymes were then used to determine the presence or absence of methylation motifs in recombinant plasmids. Digestion reactions were conducted for 4 h at 37 °C and products were separated on 1% agarose gels. Methylation motifs were further confirmed by SMRT/bisulfite sequencing of the recombinant plasmids.

### Density distributions of the GATC and CCWGG motifs in ***K. pneumoniae*** and simulated genomes

The simulated genome was generated based on the same length and GC content as the *K. pneumoniae* genomes. We then calculated the number of motifs in consecutive 1-kb segments to determine the density of GATC and CCWGG motifs across the *K. pneumoniae* and simulated genomes.

We defined high-/low-density regions by calculating the number of motifs in each 2-kb non-overlapping sliding window in the genome. Through normal distribution analysis using the “pnorm” function in R, the top and bottom 5% of regions were defined as high-density and low-density regions, respectively.

### Ka/Ks analysis of the GATC and CCWGG motif sequences and corresponding scramble sequences

We first extracted the minimum DNA sequences (2–3 codons) containing the motifs from the open reading frames of genes to obtain motif sequences. Corresponding scramble sequences were obtained by random shuffling (eliminating the motif sequences). The top 10 most frequent scramble sequences were used in subsequent analysis. We then identified the reference sequences of motif sequences/scramble sequences in the reference genome (HS11286) by Multiple Alignment using Fast Fourier Transform (MAFFT) [55]. The motif sequences, scramble sequences, and corresponding reference sequences of each strain were respectively concatenated, and their Ka/Ks ratios were calculated by ParaAT [56].

### Sequence conservation analysis of the GATC and CCWGG motifs

We extracted the methylated motifs and their flanking sequences (20 nt) in IGRs from one genome and obtained the corresponding sequences of 13 other genomes by multiple sequence alignment. Using the aforementioned 14 sequences, conservation scores were calculated by PhyloP [57]. The conservation scores of hemi-/un-methylated motifs with 20 nt flanking sequences in IGRs were calculated using the same method.

### Calculation of genomic replication termination time through simulating genomic coverage plots

Genomic coverage plots reflect the accumulated copy numbers across genomes. Since replication forks always proceed from the *oriC* to the doubling point (*s_DP_*), only a few can get close to the doubling point in the exponential phase. As a result, the sequencing coverage in the *oriC* region is much higher than that in the doubling point region. In each cell, the copy number for each site (*s*) depends on its relative position to the first replication fork (*s*_1_) and the minimal time of successive initiations (re-initiation). The re-initiation time is consistent with the *t_D_* in the exponential phase, reflecting the growth rate of *K. pneumoniae* strains.

After initiation, replication forks should advance bi-directionally at similar speeds in the exponential phase. Therefore, genome coverage plots are symmetrical near the doubling point. We divided the coverage plots into left ($s\leq s_{\mathrm{DP}}$) and right ($s\geq s_{\mathrm{DP}}$) parts by doubling point, and simulated the curves independently. We then obtained the relative position ($x\in \left(0,\right)\left(1\right]$) of each genomic site using Equation (1) where $S_{G}$ represents the half-length of the genome.

(1) $$\left\{\begin{array}{c} x=s/S_{G}\\ x=2-s/S_{G} \end{array}\right)\begin{array}{c} \left(s\leq s_{\mathrm{DP}}\right)\\ \left(s\geq s_{\mathrm{DP}}\right) \end{array}$$

We subsequently calculated the relative distance of the first replication fork when another one at *oriC* is re-initiated ($\Delta x_{R}$) using Equation (2) where *t_R_* represents the genomic replication termination time.

(2) $$\Delta x_{R}=t_{D}/t_{R}\;(0\leq t_{D}\leq t_{R})$$

By constructing the replication frequency matrix of genomic sites (*x*) with different distances from the first replication fork (*x*_1_), the copy number of the genomic site ($f_{\left(x,x_{1}\right)}$) can be determined using Equation (3). When the genomic site is in front of the first replication fork ($x\geq x_{1}$), its copy number is 1; when the site is between the first and second replication forks ($x_{1}-\Delta x_{R}\leq x\leq x_{1}$), its copy number is 2. The rest were deduced by analogy.

(3) $$f_{\left(x,\;x_{1}\right)}=\left\lceil 2^{\left\lceil \left(x_{1}-x\right)/\Delta x_{R}\right\rceil }\right\rceil$$

Next, we used the B distribution to assess cell population density with different genomic positions of the first replication forks ($x_{1}\in \left[0,1\right]$). Since *x* is not continuous data (step size = $1/S_{G}$), the cell population density of the first replication forks ($P_{\left(x_{1}\right)}$) at each genomic site was determined from the difference of accumulated densities ($I_{x}\left(\alpha ,\beta \right)$) of the adjacent sites.

(4) $$P_{\left(x_{1}\right)}=I_{{(x}_{1})}\left(\alpha ,\beta \right)-I_{\left(x_{1}-\frac{1}{S_{G}}\right)}\left(\alpha ,\beta \right)$$

Genome coverage plots could be determined as the integral of the copy numbers of each genomic site in the cellular populations $\left(H\left(x\right)\right)$ using Equation (5). The integral was calculated based on $P_{\left(x_{1}\right)}$ and the relative distance between x and $x_{1}$ ($x_{1}-x$). By substituting Equations (2)–(4) into Equation (5), we established a mathematical model to fit the genome coverage plots using Equation (6).

(5) $$H\left(x\right)=\int_{0}^{1}f_{\left(x,x_{1}\right)}*P_{\left(x_{1}\right)}d_{x_{1}}$$

(6) $$H\left(x\right)=\int_{0}^{1}\left\lceil 2^{\left\lceil \left(x_{1}-x\right)\text{*}t_{R}/t_{D}\right\rceil }\right\rceil \text{*}\left(I_{\left(x_{1}\right)}\left(\alpha ,\beta \right)-I_{\left(x_{1}-\frac{1}{S_{G}}\right)}\left(\alpha ,\beta \right)\right)d_{x_{1}}$$

We then substituted Equation (1) into Equation (6), and repeatedly fitted the genome coverage plots by selecting continuous parameters with step sizes of 0.1. We finally obtained the optimal solutions of ${\Delta x}_{R}$ and B distribution parameters (*α,β*) through goodness of fit tests. Since *t_D_* could be calculated from the growth curve of each *K. pneumoniae* strain, we could obtain the *t_R_* using Equation (2).

### Calculation of the re-modification time of motifs

In the exponential phase, the methylation read fraction (*M*_(_*_x_*_)_) of each motif was determined from its initial methylation read fraction ($M_{\left(x\right)}^{0}$), replication-induced passive demethylation, and MTase-catalyzed re-methylation. Based on *M*_(_*_x_*_)_ in the exponential phase and B distribution parameters (*α,β*), we calculated the proceeding distance of the corresponding replication fork when the hemi-methylated motif was re-methylated ($\Delta x_{M}$). As shown in Equation (7), $\Delta x_{M}$ corresponds to the ratio of mean re-methylation time (*t_M_*) to the genomic replication termination time (*t_R_*).

(7) $$\Delta x_{M}=t_{M}/t_{R}$$

When motif *x* is located downstream of a replication fork ($x_{j}\leq x$), its methylation read fraction (*M*_(_*_x_*_)_) remains unchanged due to no replication-induced passive demethylation. When motif *x* is located upstream of a replication fork with a longer distance ($x_{j}\geq x+\Delta x_{M}$), the methylation read fraction is also unchanged due to the completion of re-methylation of motif *x*. Thus, we only considered the demethylation effect of replication forks in a certain range ($x\leq x_{j}\leq \left(x+\Delta x_{M}\right)$) on *M*_(_*_x_*_)_ in the mathematical derivation, where j represents the serial number of the replication fork affecting *M*_(_*_x_*_)_.

If the hemi-methylated motifs can be rapidly re-modified before the next replication fork ($t_{M}\leq t_{D}$), we only need to evaluate the demethylation effect of one replication for all replication forks in the range of $x\leq x_{j}\leq x+\Delta x_{M}$. Here, we include the first replication forks ($x_{1}$) and other effective replication forks ($x_{j}$) in the range. We then substituted the accumulated B distribution density of the first replication forks ($x+\left(j-1\right)\Delta x_{R}\leq x_{1}\leq x+\Delta x_{M}+\left(j-1\right)\Delta x_{R}$) with that of the corresponding effective replication forks ($x\leq x_{j}\leq x+\Delta x_{M}$). Due to semi-conservative replication, we further obtain $\Delta x_{M}$ from Equation (8) where N represents the upper limit of j, which is determined by $\Delta x_{R}$ and the relative genomic position (*x*) of methylated motifs.

(8) $$\left\{\begin{array}{c} M\left(x\right)=M_{\left(x\right)}^{0}*\left(1-0.5*\sum_{j=1}^{N}\left(\begin{array}{c} I_{\left(x+\Delta x_{M}+\left(j-1\right)*\Delta x_{R}\right)}\left(\alpha ,\beta \right)-\;\\ \begin{array}{c} I_{\left(x+\left(j-1\right)*\Delta x_{R}\right)}\left(\alpha ,\beta \right) \end{array} \end{array}\right)\right)\;\\ N=\left\lceil \left(1-x\right)/\Delta x_{R}\right\rceil \end{array}\right)$$

If the methylation is slow, the hemi-methylated motifs may not be re-modified when the next replication fork crosses them ($t_{M}>t_{D}$). In this case, the effect of multiple passive demethylation on *M*_(_*_x_*_)_ should be considered using Equation (9), which is determined by the ratio of mean re-methylation time (*t_M_*) to re-initiation time ($t_{D}$), where n represents the upper limit of multiple passive demethylation times (i).

(9) $$n=\left\lceil t_{M}/t_{D}\right\rceil =\left\lceil \Delta x_{M}/\Delta x_{R}\right\rceil$$

We then converted the genomic positions of effective replication forks ($x_{j}$) into those of the corresponding first replication forks ($x_{1}$), and substituted the accumulated B distribution density of $x_{1}$ ($x+\left(j-1\right)*\Delta x_{R}\leq x_{1}\leq x+j*\Delta x_{R}$) for the $x_{j}$ population density ($x\leq x_{j}\leq x+\Delta x_{M}$). Considering the differential influences on *M*_(_*_x_*_)_ in the case of j<n and n≤j≤N, we calculated $\Delta x_{M}$ using Equation (10) by comprehensive calculations.

(10) $$\left\{\begin{array}{c} M\left(x\right)=M_{\left(x\right)}^{0}*\left(\begin{array}{c} \begin{array}{c} 1-\sum_{j=1}^{n-1}\sum_{i=1}^{j}0.5^{i}*\left(\begin{array}{c} I_{\left(x+j*\Delta x_{R}\right)}\left(\alpha ,\beta \right)-\\ I_{\left(x+\left(j-1\right)*\Delta x_{R}\right)}\left(\alpha ,\beta \right) \end{array}\right)\\ -\sum_{j=n}^{N}\sum_{i=1}^{n-1}0.5^{i}*\left(\begin{array}{c} I_{\left(x+j*\Delta x_{R}\right)}\left(\alpha ,\beta \right)-\\ I_{\left(x+\left(j-1\right)*\Delta x_{R}\right)}\left(\alpha ,\beta \right) \end{array}\right) \end{array}\\ -0.5^{n}*\sum_{j=n}^{N}\left(\begin{array}{c} I_{\left(x+\Delta x_{M}+\left(j-n\right)*\Delta x_{R}\right)}\left(\alpha ,\beta \right)-\\ \begin{array}{c} I_{\left(x+\left(j-1\right)*\Delta x_{R}\right)}\left(\alpha ,\beta \right) \end{array} \end{array}\right) \end{array}\right)\\ \begin{array}{c} n=\left\lceil t_{M}/t_{D}\right\rceil =\left\lceil \Delta x_{M}/\Delta x_{R}\right\rceil \\ N=\lceil \left(1-x\right)/\Delta x_{R}\rceil \end{array} \end{array}\right)$$

Based on the growth curves and genome coverage plots of the sequencing data, we deduced various parameters in different *K. pneumoniae* strains, including *t_D_*, *t_R_*, and $I_{x}\left(\alpha ,\beta \right)$. The mean re-methylation time (*t_M_*) of each motif could be further calculated by substituting the aforementioned parameters, Equations (2) and (7), the methylation read fraction in the exponential phase (*M*_(_*_x_*_)_), and the stationary phase ($M_{\left(x\right)}^{0}$) into Equation (10).

## Data availability

The genome data of 14 *K. pneumoniae* strains have been deposited in the Genome Sequence Archive [58] at the National Genomics Data Center, Beijing Institute of Genomics, Chinese Academy of Sciences/ China National Center for Bioinformation (GSA: CRA003482), and are publicly accessible at https://bigd.big.ac.cn/gsa. The data are also at the NCBI database (BioProject: PRJNA477755), and are publicly accessible at https://www.ncbi.nlm.nih.gov.

## CRediT author statement

**Jing Fu:** Validation, Writing - original draft, Resources. **Ju Zhang:** Methodology, Writing - original draft, Resources. **Li Yang:** Software, Formal analysis, Writing - original draft. **Nan Ding:** Formal analysis, Writing - original draft, Visualization. **Liya Yue:** Validation, Writing - original draft, Visualization. **Xiangli Zhang:** Formal analysis, Visualization. **Dandan Lu:** Formal analysis, Visualization. **Xinmiao Jia:** Visualization. **Cuidan Li:** Visualization, Data curation. **Chongye Guo:** Visualization. **Zhe Yin:** Resources. **Xiaoyuan Jiang:** Resources. **Yongliang Zhao:** Writing - review & editing. **Fei Chen:** Conceptualization, Writing - review & editing, Funding acquisition, Project administration, Supervision. **Dongsheng Zhou:** Conceptualization, Writing - review & editing, Project administration, Supervision. All authors have read and approved the final manuscript.

## Competing interests

The authors have declared no competing interests.

## Supplementary data

Supplementary File S1 **Accuracy evaluation of the mathematical model**

Supplementary Figure S1 **Phylogenetic analysis of 76 *K. pneumoniae* strains** The strains shown in red letters represent the 14 *K. pneumoniae* strains we sequenced; the strains shown in black letters indicate the 62 *K. pneumoniae* strains downloaded from NCBI. The colored strips and stars represent various types of MLSTs and serotypes of *K. pneumoniae* strains.

Supplementary Figure S2 **Genomic structural comparison of the 14 *K. pneumoniae* strains** Genomes are arranged from the top to bottom in the order of: NTUH-K2044; 11492; 11454; 11420; 11311; 23; 12208; 11305; 11021; 309074; N201205880; 13190; 283747 and 721005. The reference strain is HS11286. The same colors represent the homologous fragments as identified by the Mauve program.

Supplementary Figure S3 **Preferred flanking sequences of the unmethylated, hemimethylated, and methylated MTCGAK motifs in NTUH-K2044 and 11492** The sequence logos show the preferences of 10 nucleotides flanking the MTCGAK motifs in the two strains of 11492 (A) and NTUH-K2044 (B). The size of colored letters represents the probability of occurrence.

Supplementary Figure S4 **Characterization of seven novel 6mA MTases specificities by using restriction digestion and SMRT sequencing A.** Schematic diagram shows the whole MTase gene and its methylation motif sequence in the recombinant plasmids. We respectively cloned the MTases genes into pRRS plasmids. Type I MTases contain modification and specificity subunits, shown as blue and green bars. Type II MTases only include MTase subunits. The predicted methylation motif sequences are located downstream of the stop codon of the MTases. To identify the activity of MTases, we introduced some methylation-sensitive restriction enzymes, which recognition motifs share six bases (red dotted boxes) with the MTase motifs. If the MTases methylate the A bases (red bold letter) in the methyltransferase deficient *E. coli* strain ER2796, the restriction enzymes will fail to cut the corresponding motif sequences. **B.** Electrophoretogram identifying methylation activity of the MTases. As for KamB, the circular plasmid pRRS-KamB could not cleave into linear plasmid by the methylation-sensitive restriction enzymes, *Mfe*I and *Bst*BI (lane 4: circular plasmid control, lane 5: linear plasmid control, lane 6: plasmid pRRS-KamB cut by *Mfe*I, lane 7: plasmid pRRS-KamB cut by *Bst*BI), demonstrating that MTase KamB could successfully methylate TTC**A**N_7_TTC motif. As for KamC/D/E/F, since there were two restriction sites on the recombinant plasmids, the circular plasmid pRRS-KamC/D/E/F could cleave into a linear fragment if the motifs were methylated (lane 8/11/14/20: circular plasmid control, lane 9/12/15/21: linear plasmid control, lane 22: plasmid pRRS-KamC cut by *Pci*I, lane 13: plasmid pRRS-KamD cut by *Sca*I, lane 10: plasmid pRRS-KamE cut by *Bst*BI, lane 16: plasmid pRRS-KamF cut by *Sca*I). Similarly, as for KamA/G, there were three restriction sites on the recombinant plasmids, the circular plasmid pRRS-KamA/G could cleave into two linear fragments if the motifs were methylated (lane 1/17: circular plasmid control, lane 2/18: linear plasmid control, lane 3: plasmid pRRS-*Kam*A cut by *Bfu*AI, lane 19: plasmid pRRS-KamG cut by ScaI). For KamG, we also observed cleaved bands on the gel due to the incomplete methylation (indicated as black arrows). **C.** IPD ratio plot shows the seven methylation motifs in the plasmids. SMRT sequencing was adopted to confirm the specificities of MTases. The purple and orange bars show the IPD ratios on plus and minus strands, respectively. The red rectangle indicates the motif sequence for each MTase. The average IPD values of the methylated bases on plus and minus strands are between four and eight. M, modification subunit; S, specificity subunit.

Supplementary Figure S5 **Characterization of 5mC MTases specificity using restriction digestion A.** Schematic diagram shows the MTase gene and its methylation motif sequence in the recombinant plasmid. We cloned KcmA gene and predicted motif sequence (MT**C**GAK: at the downstream of the stop codon of the KcmA gene) into pRRS plasmid. The modification bases are marked in bold red letters. **B.** Electrophoretogram identifying the methylation activity of KcmA (a 5mC MTase). The KcmA gene was cloned into the pRRS plasmid and expressed in *E. coli* ER2796. Recombinant plasmid DNA (pRRS-KcmA) was prepared and digested by the restriction enzyme *Bsp*DI. The products were resolved on an agarose gel for analysis. Lane M: Takara trans2K plusII ladder; lane 1: recombinant plasmid pRRS-KcmA as a positive control; lane 2: linear recombinant plasmid pRRS-KcmA as negative control (digested by *Sbf*I); lane 3: pRRS-KcmA digested by *Bsp*DI to verify the methylation activity of KcmA.

Supplementary Figure S6 **Seven predicted MTases without methylation activity A.** Schematic diagram shows the seven predicted MTase genes and corresponding methylation motif in the recombinant plasmids. **B.** Electrophoretogram identifying methylation activity of the MTases. Lane 1/5/8/13/18/21/25/29: circular plasmid control; lane 2/6/9/14/19/22/26/30: linear plasmid control; lane 3: plasmid pRRS-M2B2 cut by *Mfe*I; lane 4: plasmid pRRS-M2B2 cut by *Bst*BI; lane 7: plasmid pRRS-M3B1 cut by *Bfu*AI; lane 10: plasmid pRRS-M4B1 cut by *Bfu*AI; lane 11: plasmid pRRS-M4B2 cut by *Mfe*I; lane 12: plasmid pRRS-M4B2 cut by *Bst*BI; lane 15: plasmid pRRS-M5B1 cut by *Bfu*AI; lane 16: plasmid pRRS-M5B2 cut by *Mfe*I; lane 17: plasmid pRRS-M5B2 cut by *Bst*BI; lane 20: plasmid pRRS-M8D cut by *Bfu*AI; lane 23: plasmid pRRS-M10E cut by *Bsp*EI; lane 24: plasmid pRRS-M10E cut by *Bst*BI; lane 27: plasmid pRRS-M11E cut by *Bsp*EI; lane 28: plasmid pRRS-M11E cut by *Bst*BI; lane 31: plasmid pRRS-M15G cut by *Sca*I.

Supplementary Figure S7 **COG categories of coding genes with GATC and CCWGG motifs in high-density and low-density regions** The X-axis shows the functional classes. Y-axis shows the number of genes in each functional class.

Supplementary Figure S8 **Distribution of GATC (upper panel) and CCWGG (low panel) motifs flanking the *oriC* region among the 14 *K. pneumoniae* strains** The color intensity indicates the number of motif in 1-kb window size.

Supplementary Figure S9 **Density distribution of the GATC/CCWGG motifs on the NTUH-K2044 genome and simulated genomes** The orange histograms show the density distribution of G**A**TC/C**C**WGG motifs on the simulated genomes of strain NTUH-K2044. The green histograms show the density distribution of G**A**TC/C**C**WGG on the NTUH-K2044 genome.

Supplementary Figure S10 **Density distribution of the GATC/CCWGG motifs on the 13 *K. pneumoniae* genomes and random generated genomes** The orange histograms show the density distribution of G**A**TC/C**C**WGG motifs on the randomly generated genomes. The green histograms show the density distribution of G**A**TC/C**C**WGG on the 13 *K. pneumoniae* genomes.

Supplementary Figure S11 **Ka/Ks ratios for GATC/CCWGG “motif sequences” and “scramble sequences” of the 14 *K. pneumoniae* genomes** Box plot showing the Ka/Ks ratios of G**A**TC/C**C**WGG “motif sequences” of the *K. pneumoniae* genomes. Red boxes indicate the Ka/Ks ratios of G**A**TC/C**C**WGG “motif sequences”; blue boxes indicate the corresponding “scramble sequences” as the controls.

Supplementary Figure S12 **Boxplot of conservations scores for methylated and hemi/un-methylated GATC/CCWGG motifs** Box plot shows the conservation values of G**A**TC/C**C**WGG motifs of the *K. pneumoniae* genomes. Red boxes indicate the conservation values of methylated G**A**TC/C**C**WGG motifs and their flanking regions (20 nt); blue boxes indicate the hemi/un-methylated motifs and their flanking regions (20 nt) as the controls.

Supplementary Figure S13 **Genome-wide sequencing coverage and methylation level of GATC and CCWGG motifs during cell cycles of NTUH-K2044 and 11492 A.** Genome-wide sequencing coverage versus genome position at three stages (1, 4, and 24 h) in the cell cycle of two K. pneumoniae strains. The replication bidirectionally begins from the origin (O) and completes at the terminus (T) (*i.e.*, doubling point: in the middle of genome). The bold lines approximate the average coverage across the genomes. Ratios of the average coverage at *oriC* to that at doubling point are labeled in the figure. **B.** Genome-wide methylation level versus genome position for the four motifs (G**A**TC, C**C**WGG, MT**C**GAK, and GR**A**CRAC) at the three growth stages in the cell cycle of two *K. pneumoniae* strains. The bold lines approximate the average methylation levels across the genomes (5-kb window size).

Supplementary Figure S14 **Box plot showing the re-methylation time of motifs in strain 11492 and NTUH-K2044** Box plots separately represent re-methylation time of motifs in strain 11492 (red) and NTUH-K2044 (blue). The black diamond mark represents the mean, and the transverse line represents the median.

Supplementary Figure S15 **Preferred sequences flanking the GATC and CCWGG motifs with fast and slow methylation rates in NTUH-K2044 and 11492 A.** Preferred sequences flanking G**A**TC motifs with fast and slow methylation rates. The fast methylation rate means that the FRAC values for the motif are more than 0.95; the slow methylation rate means that the FRAC values for the motif are less than 0.9. Left panel: X-axis shows the G**A**TC motif flanked by five nucleotides; Y-axis indicates the ratios of A/T/C/G. **B.** Preference sequences of flanking nucleotides of C**C**WGG motif. Quick methylation mode was defined as the FRAC values for 1 h, 4 h, and 24 h samples were all above 0.85, and the slow methylation mode was defined as the FRAC values for 1 h, 4 h, and 24 h samples were all below 0.55. X-axis indicated the positions of nucleotides, and Y-axis indicated the ratios of A/T/C/G. FRAC, fraction of methylated reads.

Supplementary Figure S16 **Re-methylation time of** G**A**TC **motifs in the *ori*C region (381 nt) of NTUH-K2044 and 11492** The DnaA boxes (blue), AT-rich region (yellow), and G**A**TC motifs (red) are labeled on the sequence. The re-methylation times of G**A**TC motifs in 11492 and NTUH-K2044 stains are marked in purple and green.

Supplementary Figure S17 **Distribution of the promoters in the upstream regions of 14 *K. pneumoniae* genomes** The X-axis shows the distance from the start codon; Y-axis shows the number of promoters locating corresponding positions of the region.

Supplementary Figure S18 **COG distributions of genes with hemi/un-methylated GATC/CCWGG motifs in intergenic regions** Box plots indicated the COG categories of coding genes with hemi/un-methylated CCWGG (A) and GATC (B) motifs in intergenic regions. X-axis shows the functional classes. Y-axis shows the ratio of genes in each functional class. [A] RNA processing and modification, [B] Chromatin structure and dynamics, [C] Energy production and conversion, [D] Cell cycle control, cell division, chromosome partitioning, [E] Amino acid transport and metabolism, [F] Nucleotide transport and metabolism, [G] Carbohydrate transport and metabolism, [H] Coenzyme transport and metabolism, [I] Lipid transport and metabolism, [J] Translation, ribosomal structure and biogenesis, [K] Transcription, [L] Replication, recombination and repair, [M] Cell wall/membrane/envelope biogenesis, [N] Cell motility, [O] Post-translational modification, protein turnover, and chaperones, [P] Inorganic ion transport and metabolism, [Q] Secondary metabolites biosynthesis, transport, and catabolism, [R] General function prediction only, [S] Function unknown, [T] Signal transduction mechanisms, [U] Intracellular trafficking, secretion, and vesicular transport, [V] Defense mechanisms.

Supplementary Figure S19 **COG distributions of genes with upstream hemi/un-methylated sites of novel motifs in intergenic regions** X-axis shows the functional classes. Y-axis shows the ratio of genes in each functional class. [C] Energy production and conversion, [D] Cell cycle control, cell division, chromosome partitioning, [E] Amino acid transport and metabolism, [F] Nucleotide transport and metabolism, [G] Carbohydrate transport and metabolism, [H] Coenzyme transport and metabolism, [I] Lipid transport and metabolism, [J] Translation, ribosomal structure and biogenesis, [K] Transcription, [L] Replication, recombination and repair, [M] Cell wall/membrane/envelope biogenesis, [N] Cell motility, [O] Post-translational modification, protein turnover, and chaperones, [P] Inorganic ion transport and metabolism, [Q] Secondary metabolites biosynthesis, transport, and catabolism, [R] General function prediction only, [S] Function unknown, [T] Signal transduction mechanisms, [U] Intracellular trafficking, secretion, and vesicular transport, [V] Defense mechanisms.

Supplementary Table S1 **Clinical information of 14 *K. pneumoniae* isolates**

Supplementary Table S2 **Sequencing data of the 14 *K. pneumoniae* strains using SMRT technology**

Supplementary Table S3 **Sequencing data of the 14 *K. pneumoniae* strains using Illumina Hiseq platform**

Supplementary Table S4 **Genomic information of the online 62 *K. pneumoniae* strains used in the phylogenetic tree**

Supplementary Table S5 **Bisulfite sequencing data of the 14 *K. pneumoniae* strains**

Supplementary Table S6 **Detailed information of the motifs and corresponding DNA MTases among the 14 *K. pneumoniae* strains**

Supplementary Table S7 **Fishers’ exact test of the un-methylated and methylated MTCGAK sites**

Supplementary Table S8 **22 predicted MTase genes and the corresponding 15 methylation motifs**

Supplementary Table S9 **Distribution of GATC motifs in GRs and IGRs among 14 *K. pneumoniae* strains**

Supplementary Table S10 **Distribution of CCWGG motifs in GRs and IGRs among 14 *K. pneumoniae* strains**

Supplementary Table S11 **Summary of genes with upstream hemi/un-methylated GATC sites shared in the 14 *K. pneumoniae* strains**

Supplementary Table S12 **Summary of genes with upstream hemi/un-methylated GATC motif clusters in the 14 *K. pneumoniae* strains**

Supplementary Table S13 **SMRT sequencing data of the samples at three growth time points (1, 4, and 24 h) of 11492 and NTUH-K2044**

Supplementary Table S14 **Bisulfite sequencing data of the samples at three growth time points (1, 4, and 24 h) of 11492 and NTUH-K2044**

Supplementary Table S15 **Slow re-methylated GATC motifs in the 5' USR of genes enriched in four COG categories**

Supplementary Table S16 **The seven CCWGG sites changed in methylation state during the growth phase of NTUH-K2044**

Supplementary Table S17 **Summary of the genes with upstream hemi/un-methylated MTCGAK sites shared in the NUTH-K2044 and 11492 strains**

Supplementary Table S18 **Summary of the genes with upstream hemi/un-methylated CCAYN7TTYG sites shared in the 23 and 11311 strains**

Supplementary Table S19 **Oligonucleotide primers used in this study**
