# Supplementary material for: Precision Methylome and In Vivo Methylation Kinetics Characterization of Klebsiella pneumoniae
Source: Genomics Proteomics Bioinformatics. 2021 Jun 29;20(2):418–34. doi: 10.1016/j.gpb.2021.04.002 (PMC9684165; doi:10.1016/j.gpb.2021.04.002)
Supplement: Supplementary File S1 — Accuracy evaluation of the mathematical model [file mmc1.docx]

**File S1 Accuracy evaluation of the mathematical model**

To evaluate the accuracy of the mathematical model, we first obtained the fitting methylated-read ratios (FRAC_model_) of GATC and CCWGG motifs in the exponential phase through the methylation kinetic formula (Materials and methods). Then we compared them with the real methylated-read ratios (FRAC_PacBio_) obtained from PacBio sequencing data. As shown in Figure, red and blue lines indicate the fitting and real methylated-read ratios (FRAC_model_ and FRAC_PacBio_). We found that the fitting and real methylated-read ratios are basically consistent, indicating the accuracy of the mathematical model. Furthermore, quantitative study showed lower percentage errors between FRAC_model_ and FRAC_PacBio_ (G**A**TC motif: median = 2.79%, C**C**WGG motif: median = 0.96%), indicating the accuracy of the mathematical model again.

In addition, previous reports indicated the delayed modification of GATC motifs in the *oriC* region of *E. coli*, since the competitive occupation of the motif sites between Dam and SeqA [1–3]. This is consistent with our findings, in which the GATC motifs in the *oriC* region of *K. pneumoniae* strains had slower re-methylation rates (10.35 ± 8.69 min) than in whole genome (3.523 ± 5.142) (Figure 6B). These also support our re-modification time model on the other side.


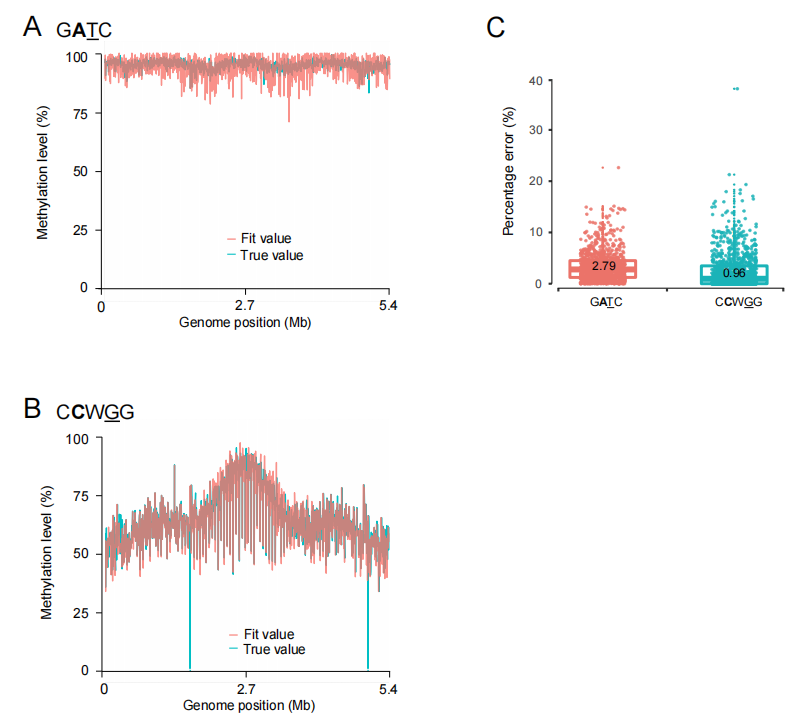


**Figure Accuracy evaluation of the mathematical model**

**A.** Comparison between the fitting and real methylated-read ratios (FRAC_model_ and FRAC_PacBio_) of G**A**TC motif (5-kb window). **B.** Comparison between the fitting and real methylated-read ratios (FRAC_model_ and FRAC_PacBio_) of C**C**WGG motif (5-kb window). **C.** Boxplot showing the percentage errors of G**A**TC and C**C**WGG motifs. FRAC, fraction of methylated reads.

**Reference**

[1] Nievera C, JJC Torgue, JE Grimwade, AC Leonard. SeqA blocking of DnaA-*oriC* interactions ensures staged assembly of the *E. coli* pre-RC. Mol Cell 2006;24:581–92.

[2] Wolanski M, Donczew R, Zawilak-Pawlik A, Zakrzewska-Czerwinska J. *oriC*-encoded instructions for the initiation of bacterial chromosome replication. Front Microbiol 2015;5:735.

[3] Lobner-Olesen A, Hansen FG, Rasmussen KV, Martin B, Kuempel PL. The initiation cascade for chromosome replication in wild-type and Dam methyltransferase deficient *Escherichia coli* cells. EMBO J 1994;13:1856–62.
