# Supplementary figures and images for: Precision Methylome and In Vivo Methylation Kinetics Characterization of Klebsiella pneumoniae
Source: Genomics Proteomics Bioinformatics. 2021 Jun 29;20(2):418–34. doi: 10.1016/j.gpb.2021.04.002 (PMC9684165; doi:10.1016/j.gpb.2021.04.002)

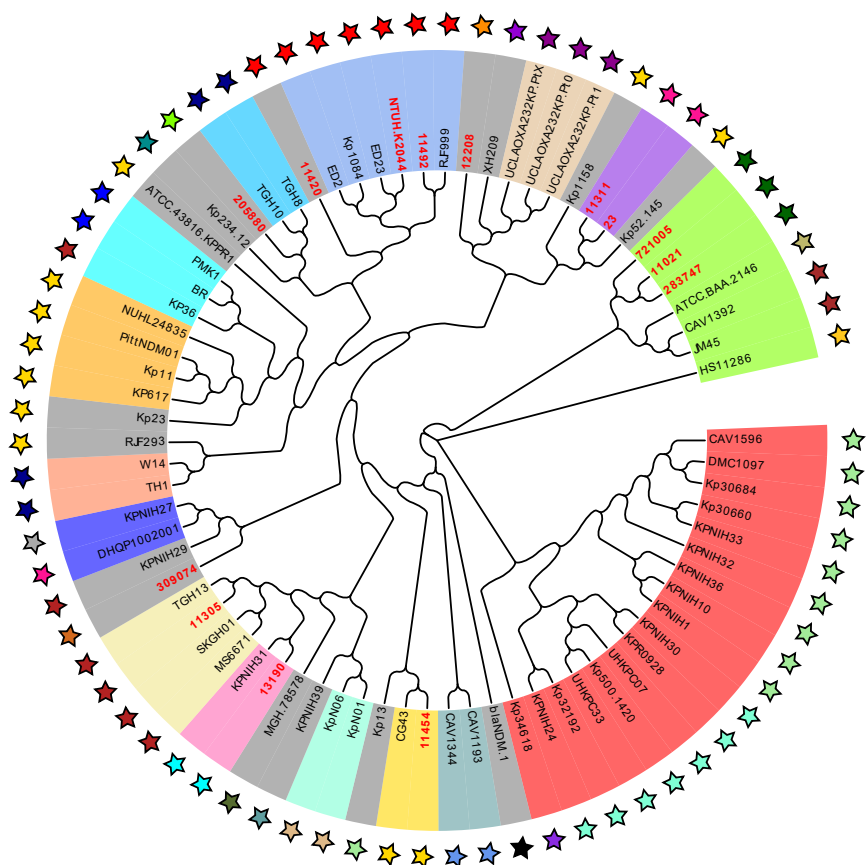

- ST258
- ST16
- ST1536
- ST383
- ST15
- ST14
- ST23
- ST86
- ST34
- ST412
- ST392
- ST147
- ST278
- ST941
- ST11
- Others

- K1
- K2
- K21
- K24
- K25
- K27
- K30
- K35
- K37
- K38
- K47
- K51
- K52
- K54
- K57
- K63
- K64
- K74
- K103
- K106
- K107
- K108
- K112
- K125
- K136
- K15

Supplement: Supplementary Figure S1 — Phylogenetic analysis of 76 K. pneumoniae strains The strains shown in red letters represent the 14 K. pneumoniae strains we sequenced; the strains shown in black letters indicate the 62 K. pneumoniae strains downloaded from NCBI. The colored strips and stars represent various types of MLSTs and serotypes of K. pneumoniae strains. [file mmc2.pdf]

## Strain

HS11286

NTUH-K2044

11492

11454

11420

11311

23

12208

11305

11021

309074

N201205880

13190

383747

721005

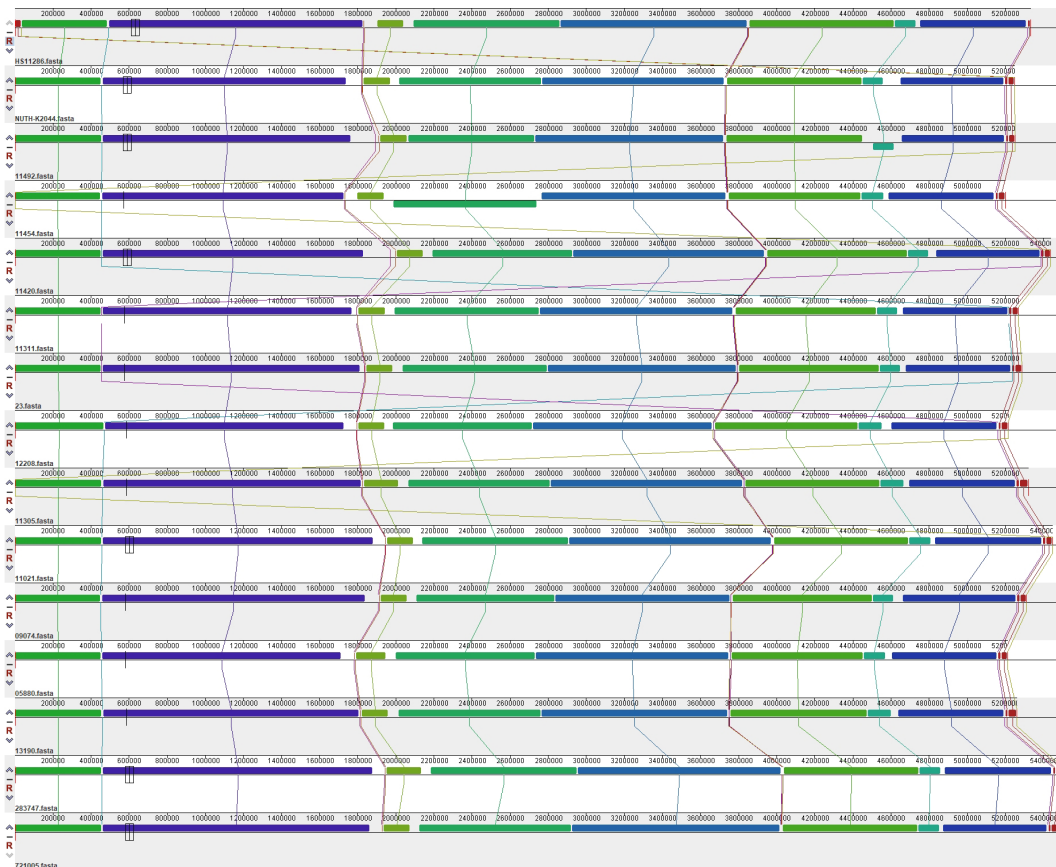

Supplement: Supplementary Figure S2 — Genomic structural comparison of the 14 K. pneumoniae strains Genomes are arranged from the top to bottom in the order of: NTUH-K2044; 11492; 11454; 11420; 11311; 23; 12208; 11305; 11021; 309074; N201205880; 13190; 283747 and 721005. The reference strain is HS11286. The same colors represent the homologous fragments as identified by the Mauve program. [file mmc3.pdf]

A 11492

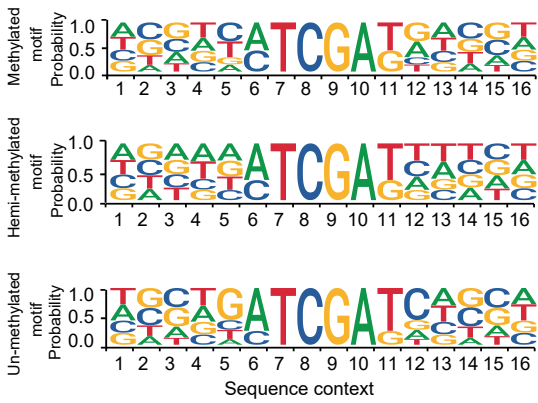

B NTUH-K2044

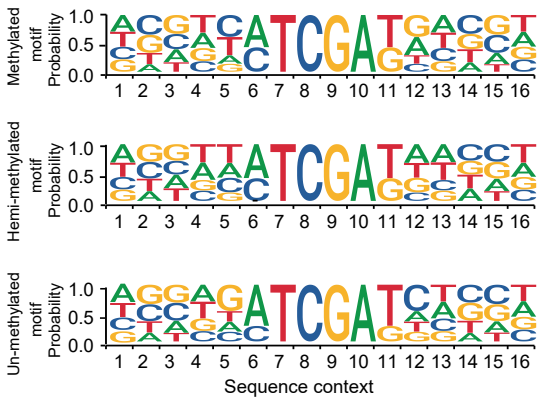

Supplement: Supplementary Figure S3 — Preferred flanking sequences of the unmethylated, hemimethylated, and methylated MTCGAK motifs in NTUH-K2044 and 11492 The sequence logos show the preferences of 10 nucleotides flanking the MTCGAK motifs in the two strains of 11492 (A) and NTUH-K2044 (B). The size of colored letters represents the probability of occurrence. [file mmc4.pdf]

A

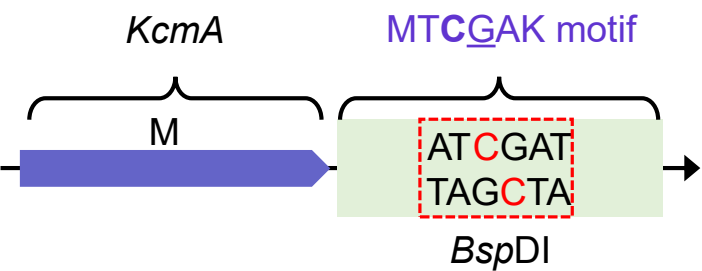

B

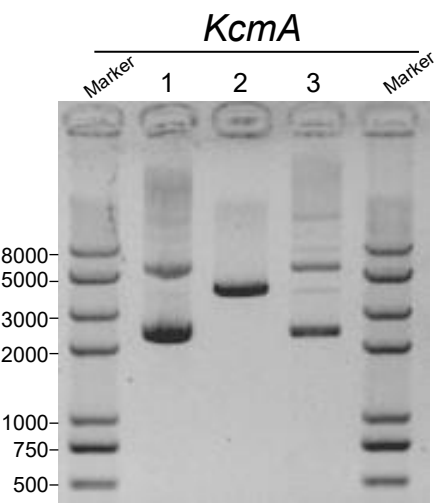

Supplement: Supplementary Figure S5 — Characterization of 5mC MTases specificity using restriction digestion A. Schematic diagram shows the MTase gene and its methylation motif sequence in the recombinant plasmid. We cloned KcmA gene and predicted motif sequence (MTCGAK: at the downstream of the stop codon of the KcmA gene) into pRRS plasmid. The modification bases are marked in bold red letters. B. Electrophoretogram identifying the methylation activity of KcmA (a 5mC MTase). The KcmA gene was cloned into the pRRS plasmid and expressed in E. coli ER2796. Recombinant plasmid DNA (pRRS-KcmA) was prepared and digested by the restriction enzyme BspDI. The products were resolved on an agarose gel for analysis. Lane M: Takara trans2K plusII ladder; lane 1: recombinant plasmid pRRS-KcmA as a positive control; lane 2: linear recombinant plasmid pRRS-KcmA as negative control (digested by SbfI); lane 3: pRRS-KcmA digested by BspDI to verify the methylation activity of KcmA. [file mmc6.pdf]

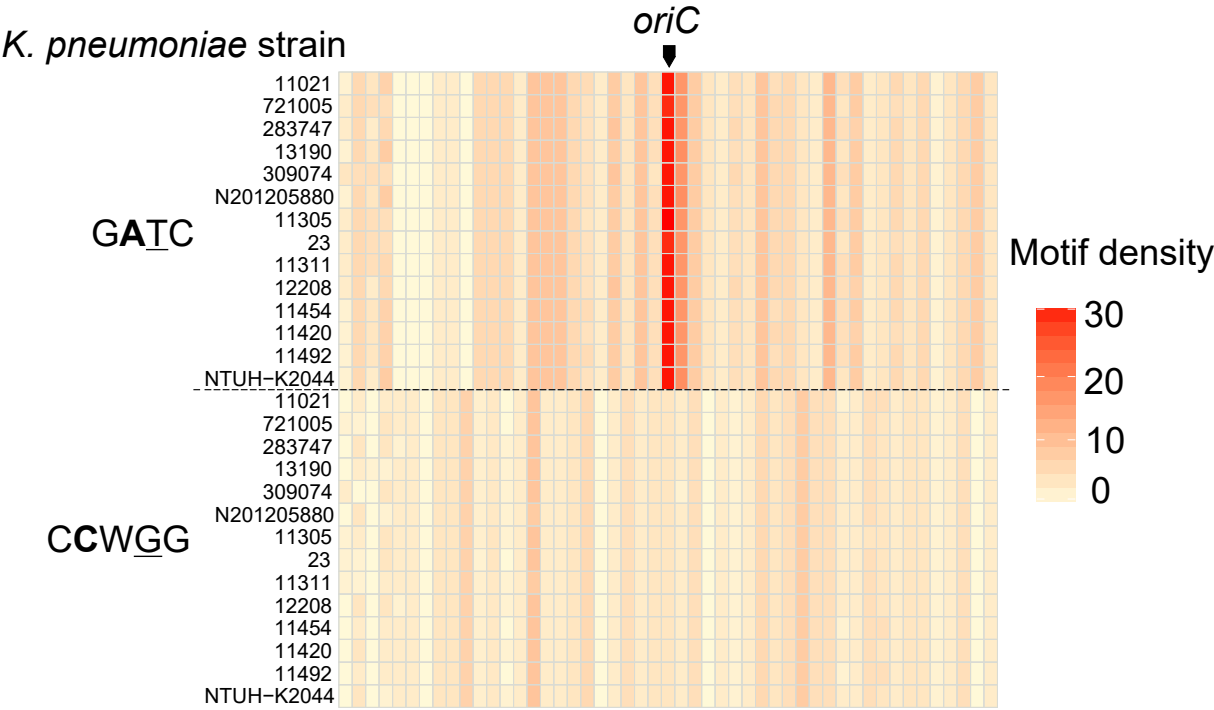

Supplement: Supplementary Figure S8 — Distribution of GATC (upper panel) and CCWGG (low panel) motifs flanking the oriC region among the 14 K. pneumoniae strains The color intensity indicates the number of motif in 1-kb window size. [file mmc9.pdf]

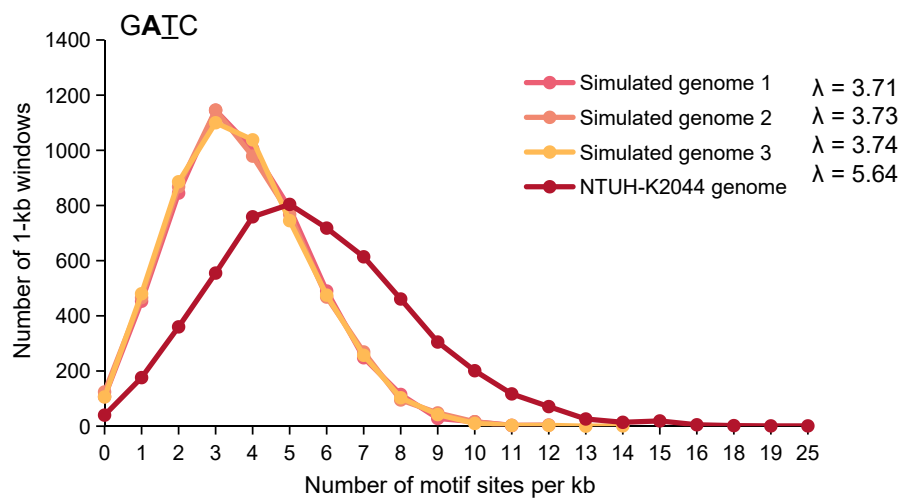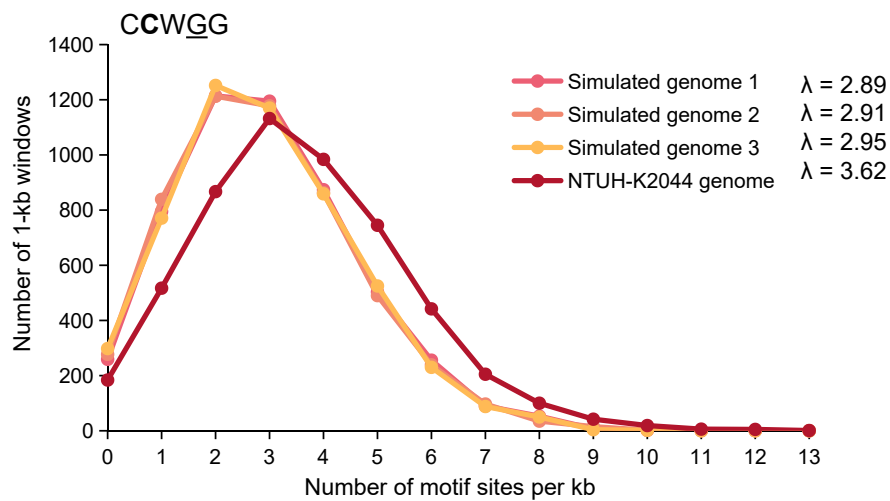

Supplement: Supplementary Figure S9 — Density distribution of the GATC/CCWGG motifs on the NTUH-K2044 genome and simulated genomes The orange histograms show the density distribution of GATC/CCWGG motifs on the simulated genomes of strain NTUH-K2044. The green histograms show the density distribution of GATC/CCWGG on the NTUH-K2044 genome. [file mmc10.pdf]

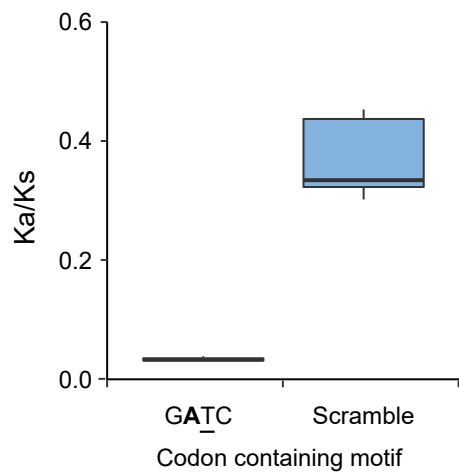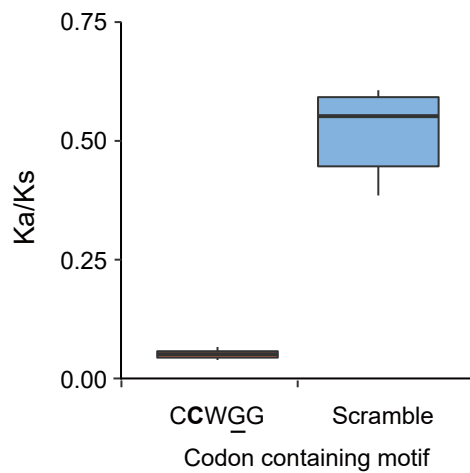

Supplement: Supplementary Figure S11 — Ka/Ks ratios for GATC/CCWGG “motif sequences” and “scramble sequences” of the 14 K. pneumoniae genomes Box plot showing the Ka/Ks ratios of GATC/CCWGG “motif sequences” of the K. pneumoniae genomes. Red boxes indicate the Ka/Ks ratios of GATC/CCWGG “motif sequences”; blue boxes indicate the corresponding “scramble sequences” as the controls. [file mmc12.pdf]

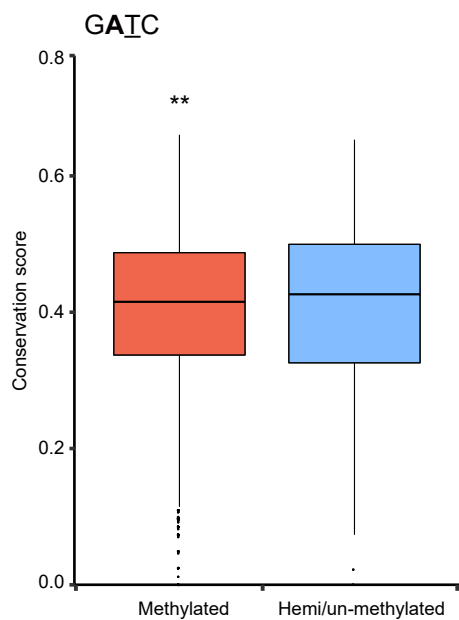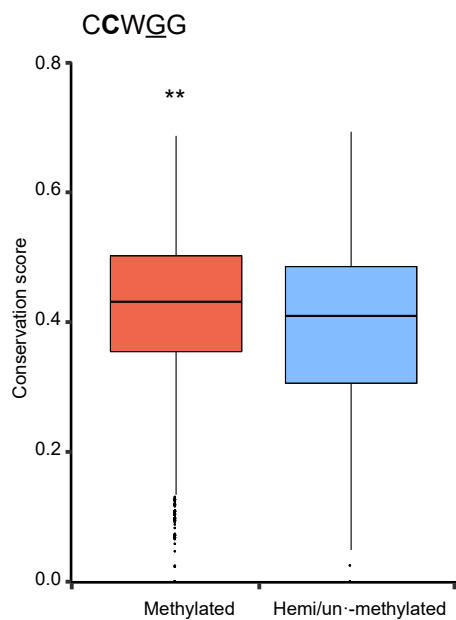

Supplement: Supplementary Figure S12 — Boxplot of conservations scores for methylated and hemi/un-methylated GATC/CCWGG motifs Box plot shows the conservation values of GATC/CCWGG motifs of the K. pneumoniae genomes. Red boxes indicate the conservation values of methylated GATC/CCWGG motifs and their flanking regions (20 nt); blue boxes indicate the hemi/un-methylated motifs and their flanking regions (20 nt) as the controls. [file mmc13.pdf]

## A Sequencing coverage

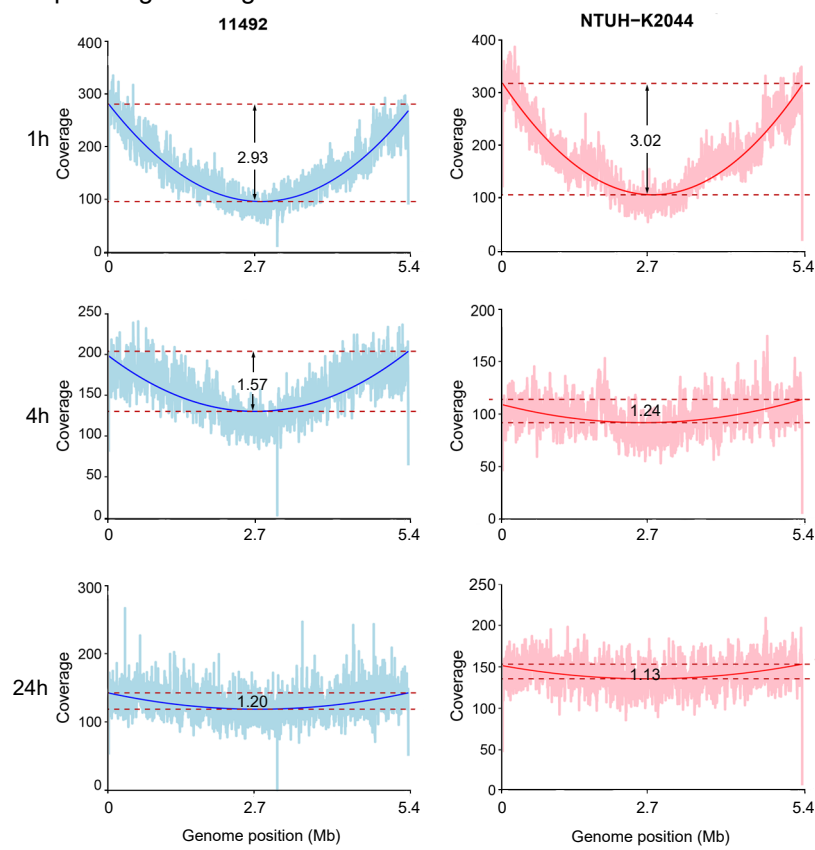

## B Methylation level

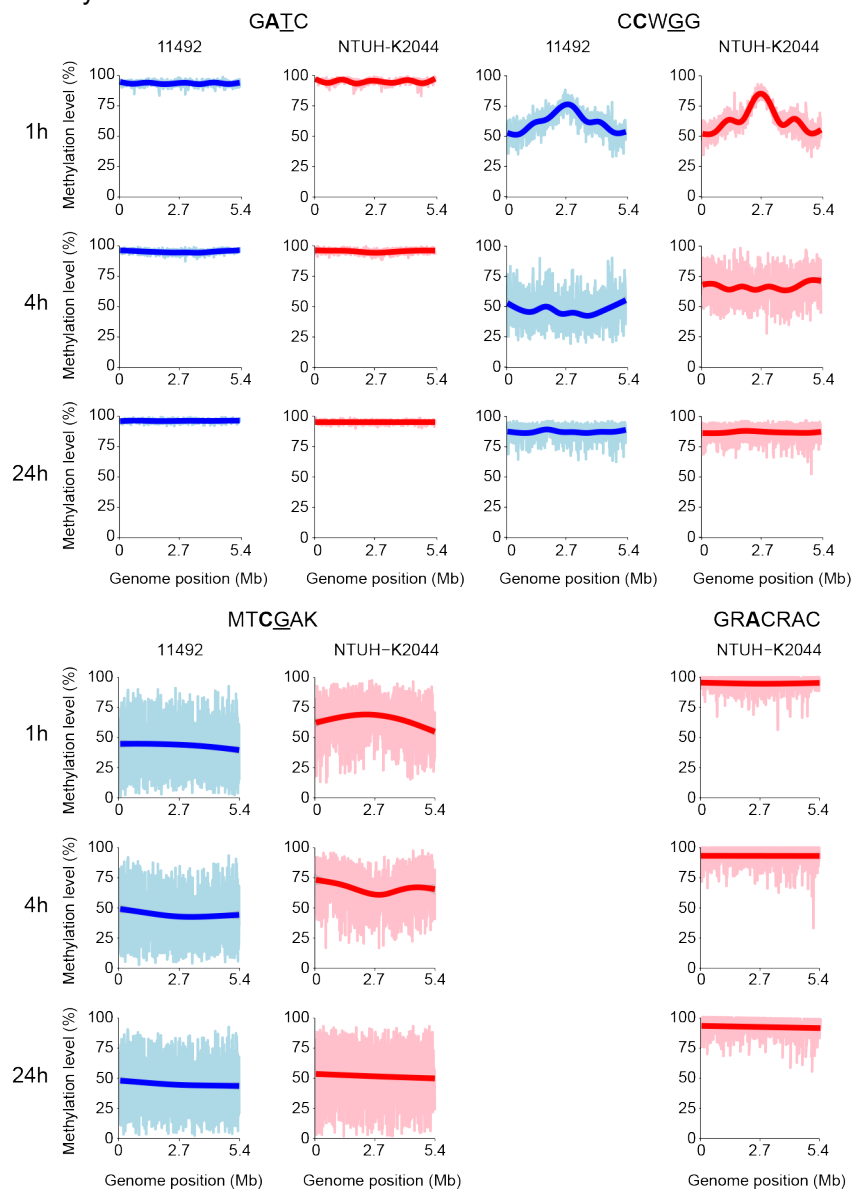

Supplement: Supplementary Figure S13 — Genome-wide sequencing coverage and methylation level of GATC and CCWGG motifs during cell cycles of NTUH-K2044 and 11492 A. Genome-wide sequencing coverage versus genome position at three stages (1, 4, and 24 h) in the cell cycle of two K. pneumoniae strains. The replication bidirectionally begins from the origin (O) and completes at the terminus (T) (i.e., doubling point: in the middle of genome). The bold lines approximate the average coverage across the genomes. Ratios of the average coverage at oriC to that at doubling point are labeled in the figure. B. Genome-wide methylation level versus genome position for the four motifs (GATC, CCWGG, MTCGAK, and GRACRAC) at the three growth stages in the cell cycle of two K. pneumoniae strains. The bold lines approximate the average methylation levels across the genomes (5-kb window size). [file mmc14.pdf]

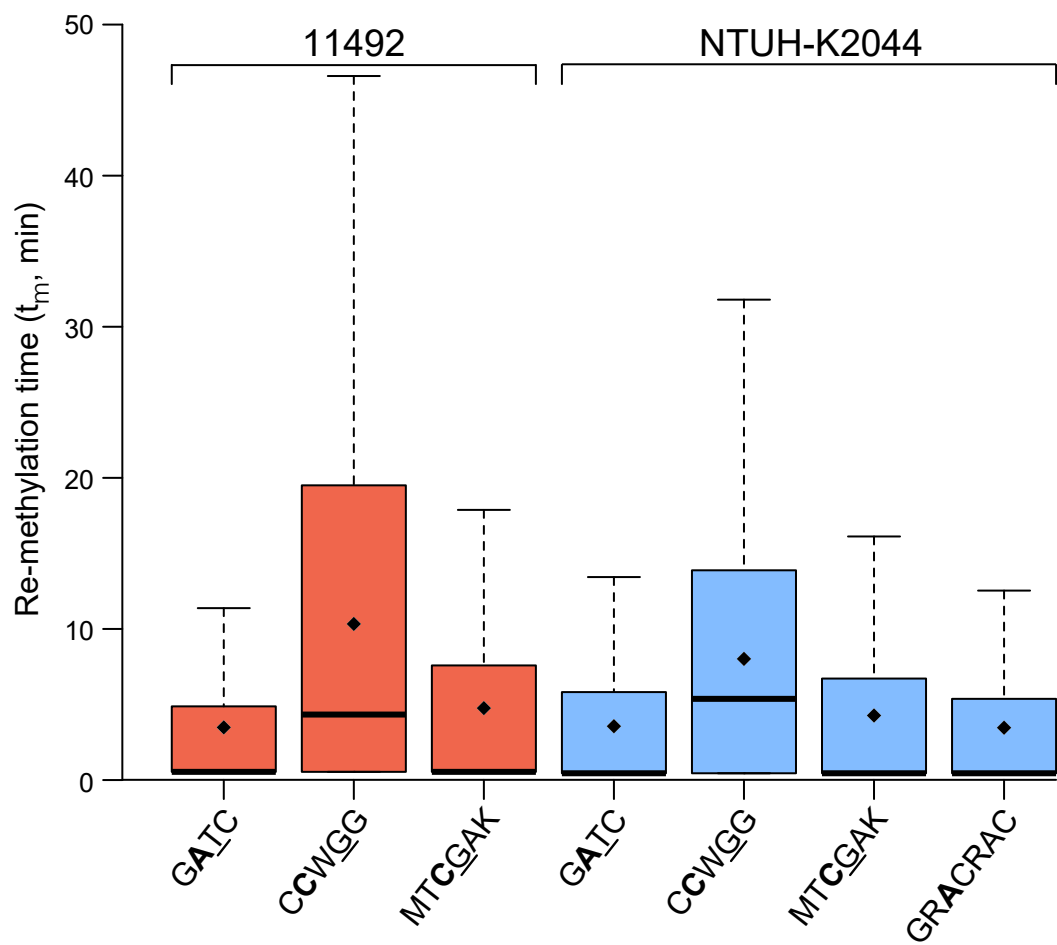

Supplement: Supplementary Figure S14 — Box plot showing the re-methylation time of motifs in strain 11492 and NTUH-K2044 Box plots separately represent re-methylation time of motifs in strain 11492 (red) and NTUH-K2044 (blue). The black diamond mark represents the mean, and the transverse line represents the median. [file mmc15.pdf]

# A GATC

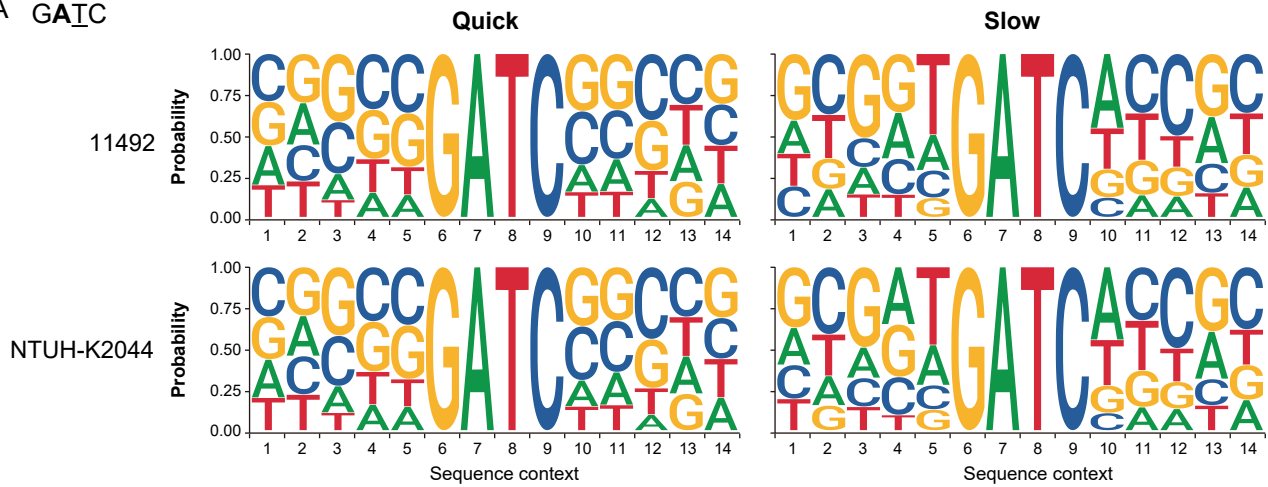

# B CCWGG

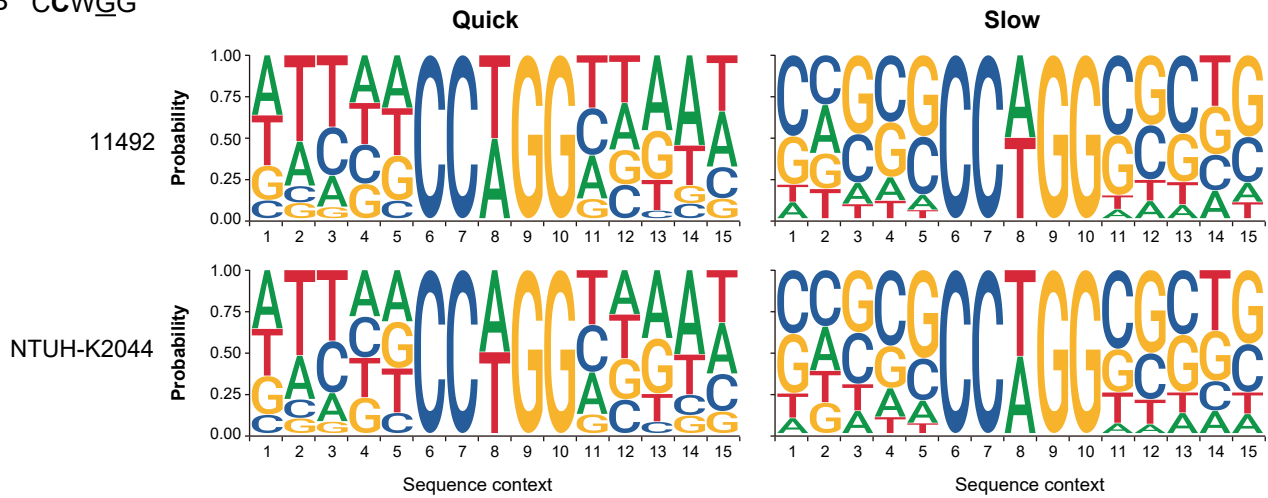

Supplement: Supplementary Figure S15 — Preferred sequences flanking the GATC and CCWGG motifs with fast and slow methylation rates in NTUH-K2044 and 11492 A. Preferred sequences flanking GATC motifs with fast and slow methylation rates. The fast methylation rate means that the FRAC values for the motif are more than 0.95; the slow methylation rate means that the FRAC values for the motif are less than 0.9. Left panel: X-axis shows the GATC motif flanked by five nucleotides; Y-axis indicates the ratios of A/T/C/G. B. Preference sequences of flanking nucleotides of CCWGG motif. Quick methylation mode was defined as the FRAC values for 1 h, 4 h, and 24 h samples were all above 0.85, and the slow methylation mode was defined as the FRAC values for 1 h, 4 h, and 24 h samples were all below 0.55. X-axis indicated the positions of nucleotides, and Y-axis indicated the ratios of A/T/C/G. FRAC, fraction of methylated reads. [file mmc16.pdf]

Promoter distribution

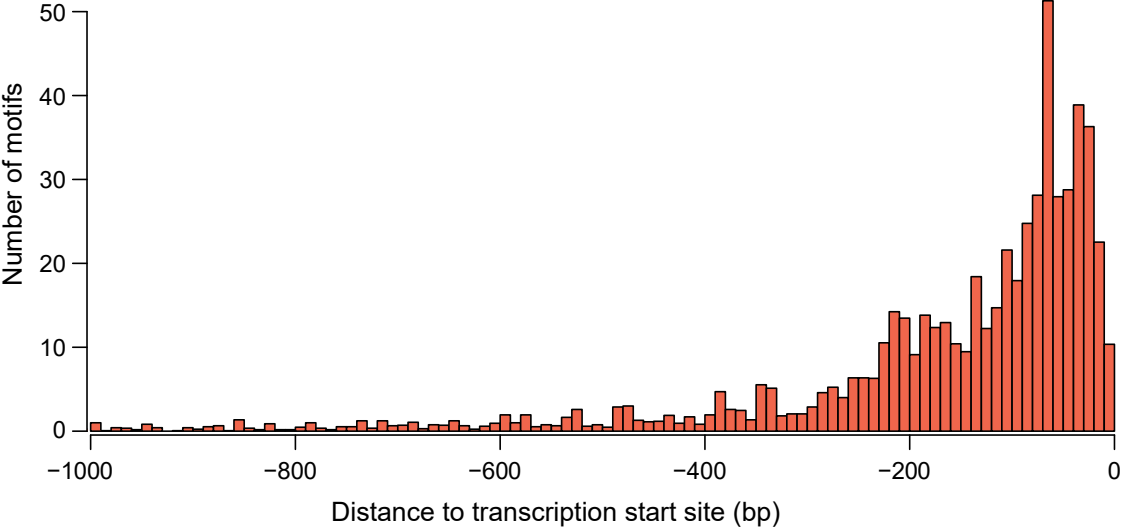

Supplement: Supplementary Figure S17 — Distribution of the promoters in the upstream regions of 14 K. pneumoniae genomes The X-axis shows the distance from the start codon; Y-axis shows the number of promoters locating corresponding positions of the region. [file mmc18.pdf]

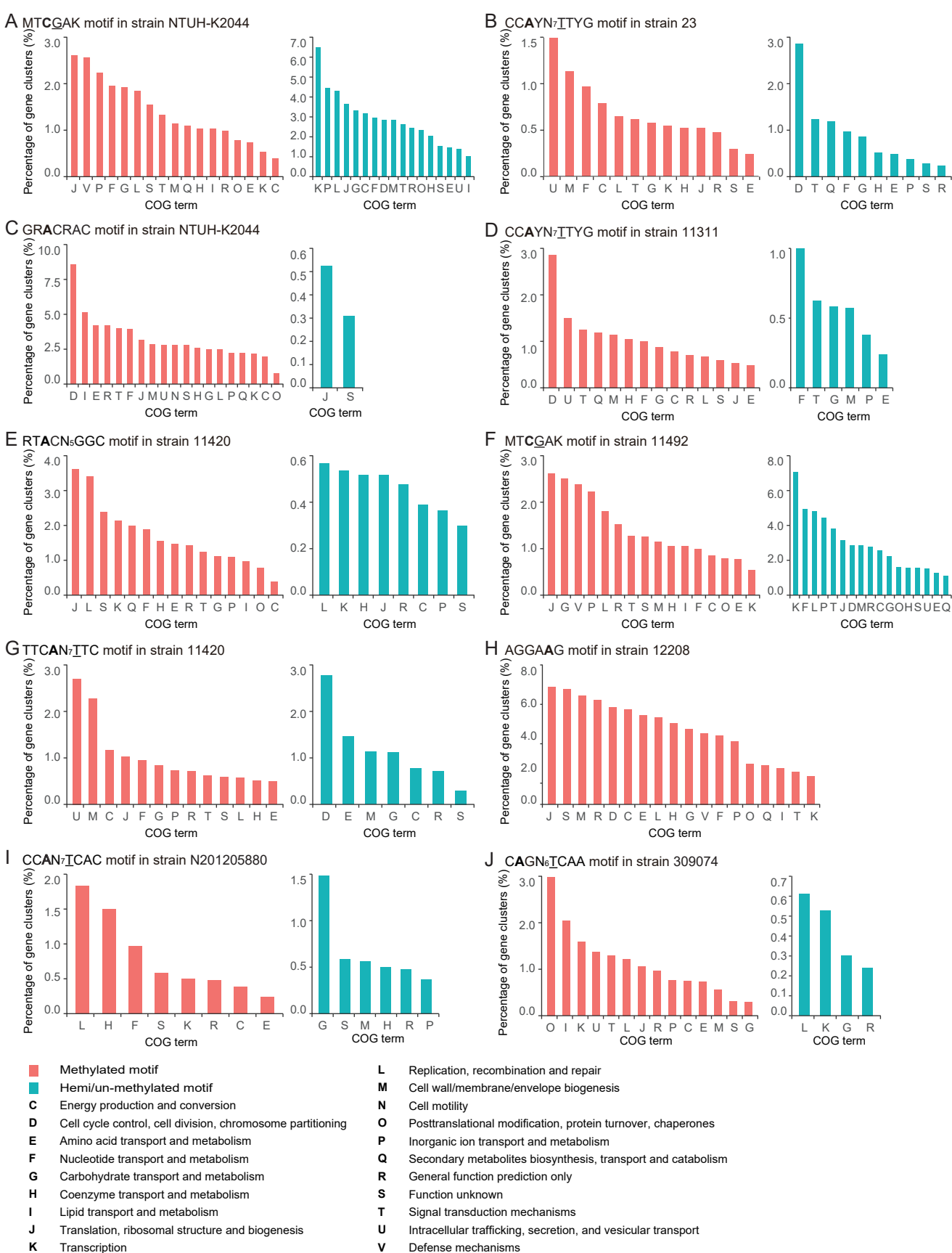

Supplement: Supplementary Figure S19 — COG distributions of genes with upstream hemi/un-methylated sites of novel motifs in intergenic regions X-axis shows the functional classes. Y-axis shows the ratio of genes in each functional class. [C] Energy production and conversion, [D] Cell cycle control, cell division, chromosome partitioning, [E] Amino acid transport and metabolism, [F] Nucleotide transport and metabolism, [G] Carbohydrate transport and metabolism, [H] Coenzyme transport and metabolism, [I] Lipid transport and metabolism, [J] Translation, ribosomal structure and biogenesis, [K] Transcription, [L] Replication, recombination and repair, [M] Cell wall/membrane/envelope biogenesis, [N] Cell motility, [O] Post-translational modification, protein turnover, and chaperones, [P] Inorganic ion transport and metabolism, [Q] Secondary metabolites biosynthesis, transport, and catabolism, [R] General function prediction only, [S] Function unknown, [T] Signal transduction mechanisms, [U] Intracellular trafficking, secretion, and vesicular transport, [V] Defense mechanisms. [file mmc20.pdf]
