# Supplementary material for: Precision Methylome and In Vivo Methylation Kinetics Characterization of Klebsiella pneumoniae
Source: Genomics Proteomics Bioinformatics. 2021 Jun 29;20(2):418–34. doi: 10.1016/j.gpb.2021.04.002 (PMC9684165; doi:10.1016/j.gpb.2021.04.002)
Supplement: Supplementary Figure S4 — Characterization of seven novel 6mA MTases specificities by using restriction digestion and SMRT sequencing A. Schematic diagram shows the whole MTase gene and its methylation motif sequence in the recombinant plasmids. We respectively cloned the MTases genes into pRRS plasmids. Type I MTases contain modification and specificity subunits, shown as blue and green bars. Type II MTases only include MTase subunits. The predicted methylation motif sequences are located downstream of the stop codon of the MTases. To identify the activity of MTases, we introduced some methylation-sensitive restriction enzymes, which recognition motifs share six bases (red dotted boxes) with the MTase motifs. If the MTases methylate the A bases (red bold letter) in the methyltransferase deficient E. coli strain ER2796, the restriction enzymes will fail to cut the corresponding motif sequences. B. Electrophoretogram identifying methylation activity of the MTases. As for KamB, the circular plasmid pRRS-KamB could not cleave into linear plasmid by the methylation-sensitive restriction enzymes, MfeI and BstBI (lane 4: circular plasmid control, lane 5: linear plasmid control, lane 6: plasmid pRRS-KamB cut by MfeI, lane 7: plasmid pRRS-KamB cut by BstBI), demonstrating that MTase KamB could successfully methylate TTCAN7TTC motif. As for KamC/D/E/F, since there were two restriction sites on the recombinant plasmids, the circular plasmid pRRS-KamC/D/E/F could cleave into a linear fragment if the motifs were methylated (lane 8/11/14/20: circular plasmid control, lane 9/12/15/21: linear plasmid control, lane 22: plasmid pRRS-KamC cut by PciI, lane 13: plasmid pRRS-KamD cut by ScaI, lane 10: plasmid pRRS-KamE cut by BstBI, lane 16: plasmid pRRS-KamF cut by ScaI). Similarly, as for KamA/G, there were three restriction sites on the recombinant plasmids, the circular plasmid pRRS-KamA/G could cleave into two linear fragments if the motifs were methylated (lane 1/17: circular plasmid control, lane 2/18: l [file mmc5.pdf]

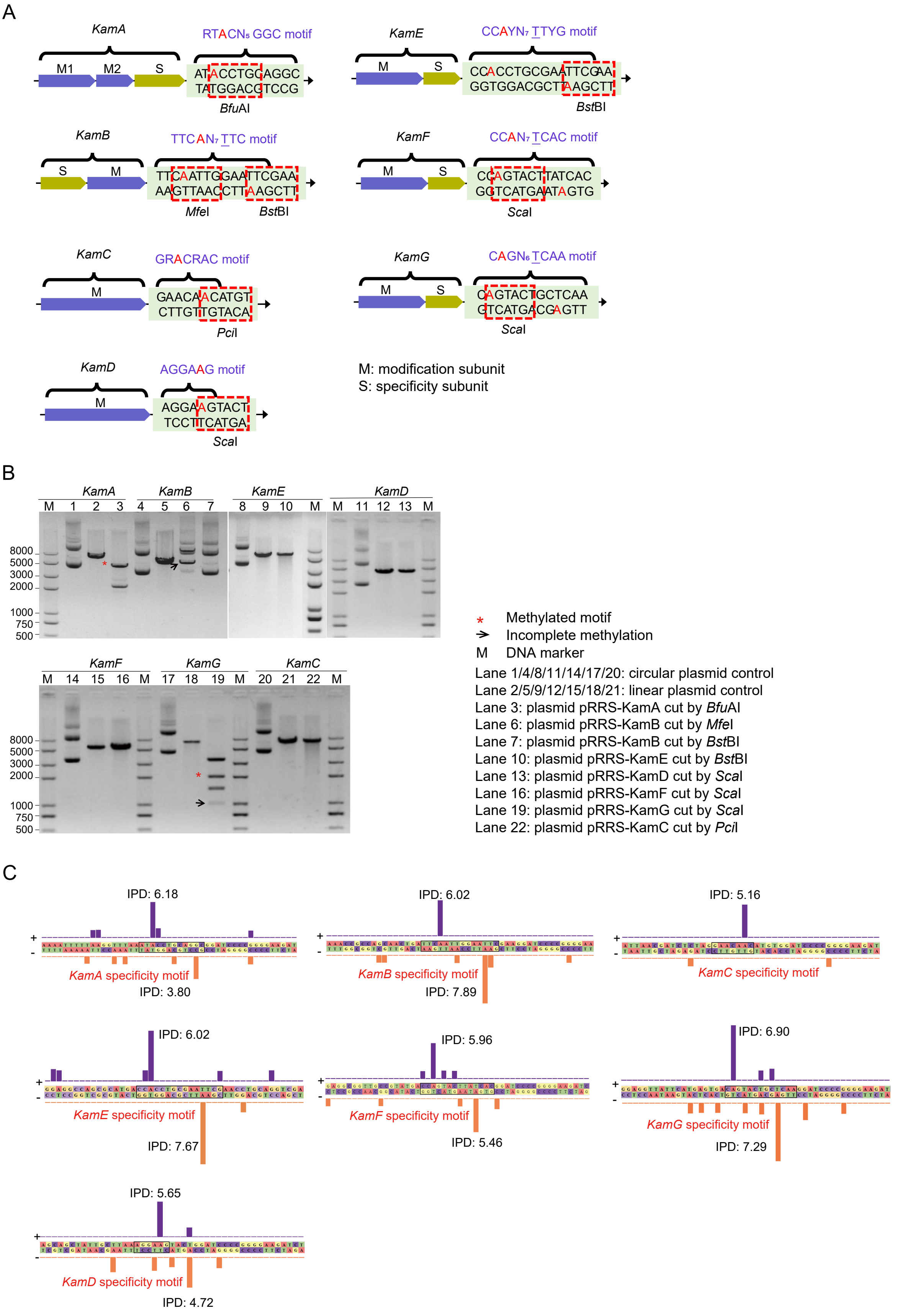

A
KamA
RTACN5 GGC motif
M1
M2
S
ATACCTGCAGGC
TATGGACGTCCG
BfuAI
KamE
CCAYN7 TTYG motif
M
S
CCACCTGCGAATTCGAA
GGTGGACGCTTAAGCTT
BstBI
KamB
TTCAN7 TTC motif
S
M
TTCAATTGGAATTCGAA
AAGTTAACCTTAAGCTT
MfeI
BstBI
KamF
CCAN7 TCAC motif
M
S
CCAGTACTTATCAC
GGTCATGAATAGTG
ScaI
KamC
GRACRAC motif
M
GAACAACATGT
CTTGTTGTACA
PciI
KamG
CAGN6 TCAA motif
M
S
CAGTACTGCTCAA
GTCATGACGAGTT
ScaI
KamD
AGGAAG motif
M
AGGAAGTACT
TCCTTCATGA
ScaI
M: modification subunit
S: specificity subunit
B
KamA
KamB
KamE
KamD
M 1 2 3 4 5 6 7 8 9 10 M M 11 12 13 M
8000
5000
3000
2000
1000
750
500
KamF
KamG
KamC
M 14 15 16 M 17 18 19 M 20 21 22 M
* Methylated motif
Incomplete methylation
M DNA marker
Lane 1/4/8/11/14/17/20: circular plasmid control
Lane 2/5/9/12/15/18/21: linear plasmid control
Lane 3: plasmid pRRS-KamA cut by BfuAI
Lane 6: plasmid pRRS-KamB cut by MfeI
Lane 7: plasmid pRRS-KamB cut by BstBI
Lane 10: plasmid pRRS-KamE cut by BstBI
Lane 13: plasmid pRRS-KamD cut by ScaI
Lane 16: plasmid pRRS-KamF cut by ScaI
Lane 19: plasmid pRRS-KamG cut by ScaI
Lane 22: plasmid pRRS-KamC cut by PciI
C
IPD: 6.18
KamA specificity motif
IPD: 3.80
IPD: 6.02
KamB specificity motif
IPD: 7.89
IPD: 5.16
KamC specificity motif
IPD: 6.02
KamE specificity motif
IPD: 7.67
IPD: 5.96
KamF specificity motif
IPD: 5.46
IPD: 6.90
KamG specificity motif
IPD: 7.29
IPD: 5.65
KamD specificity motif
IPD: 4.72
