# Supplementary material for: Precision Methylome and In Vivo Methylation Kinetics Characterization of Klebsiella pneumoniae
Source: Genomics Proteomics Bioinformatics. 2021 Jun 29;20(2):418–34. doi: 10.1016/j.gpb.2021.04.002 (PMC9684165; doi:10.1016/j.gpb.2021.04.002)
Supplement: Supplementary Figure S6 — Seven predicted MTases without methylation activity A. Schematic diagram shows the seven predicted MTase genes and corresponding methylation motif in the recombinant plasmids. B. Electrophoretogram identifying methylation activity of the MTases. Lane 1/5/8/13/18/21/25/29: circular plasmid control; lane 2/6/9/14/19/22/26/30: linear plasmid control; lane 3: plasmid pRRS-M2B2 cut by MfeI; lane 4: plasmid pRRS-M2B2 cut by BstBI; lane 7: plasmid pRRS-M3B1 cut by BfuAI; lane 10: plasmid pRRS-M4B1 cut by BfuAI; lane 11: plasmid pRRS-M4B2 cut by MfeI; lane 12: plasmid pRRS-M4B2 cut by BstBI; lane 15: plasmid pRRS-M5B1 cut by BfuAI; lane 16: plasmid pRRS-M5B2 cut by MfeI; lane 17: plasmid pRRS-M5B2 cut by BstBI; lane 20: plasmid pRRS-M8D cut by BfuAI; lane 23: plasmid pRRS-M10E cut by BspEI; lane 24: plasmid pRRS-M10E cut by BstBI; lane 27: plasmid pRRS-M11E cut by BspEI; lane 28: plasmid pRRS-M11E cut by BstBI; lane 31: plasmid pRRS-M15G cut by ScaI. [file mmc7.pdf]

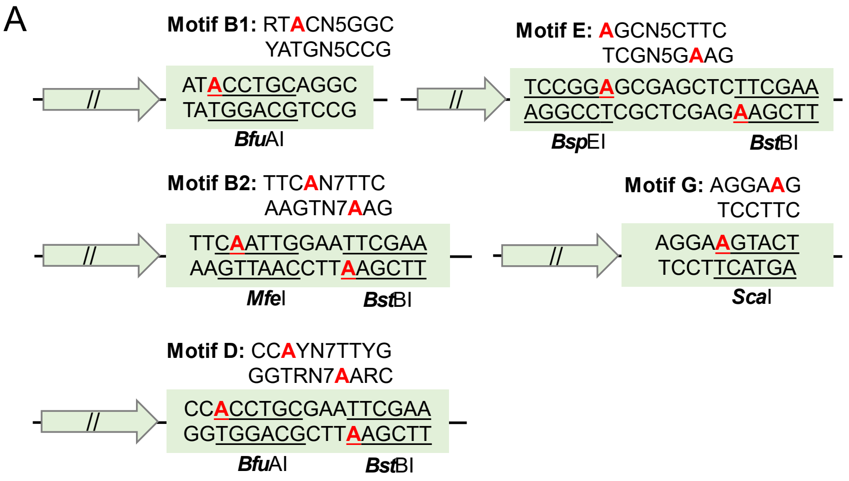

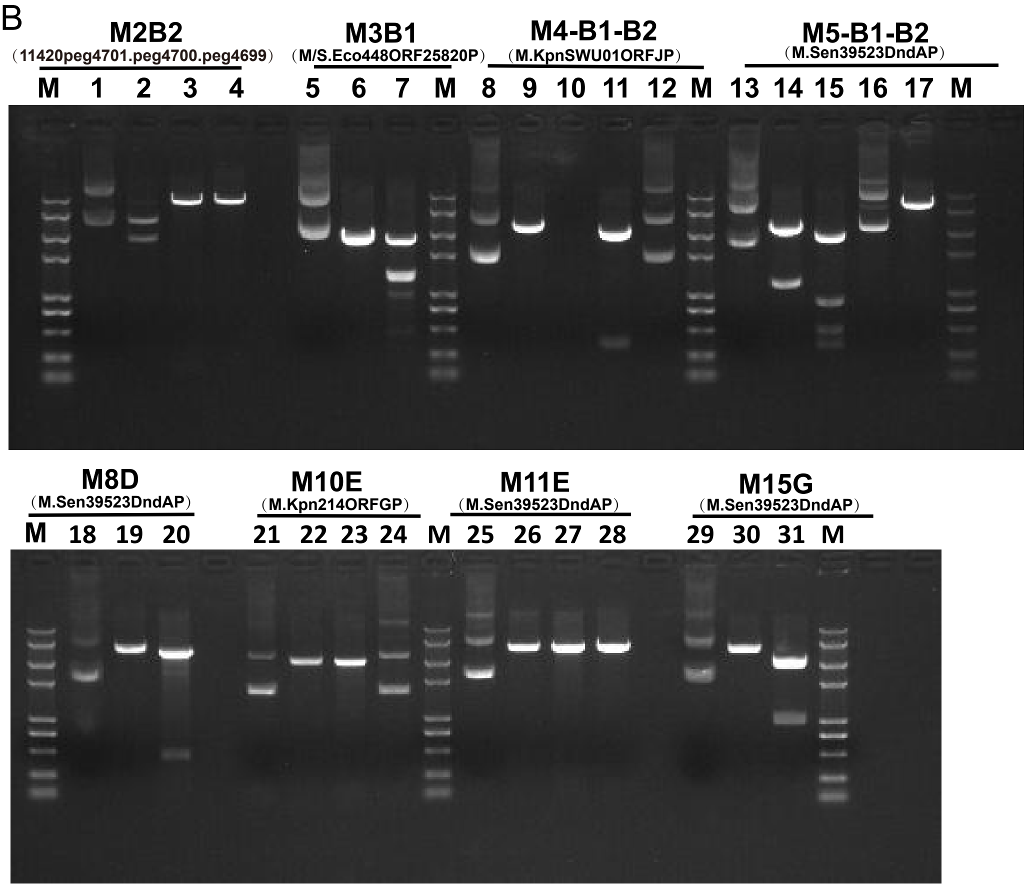

Lane M	Takara trans2K plusII ladder	Lane 15	Plasmid pRRS-M5B1 cut by *Bfu*AI
Lane 1/5/8/13/18/21/25/29	Circular plasmid contro II	Lane 16	Plasmid pRRS-M5B2 cut by *Mfe*I
Lane 2/6/9/14/19/22/26/30	Linear plasmid control	Lane 17	Plasmid pRRS-M5B2 cut by *Bst*BI
Lane 3	Plasmid pRRS-M2B2 cut by *Mfe*I	Lane 20	Plasmid pRRS-M8D cut by *Bfu*AI
Lane 4	Plasmid pRRS-M2B2 cut by *Bst*BI	Lane 23	Plasmid pRRS-M10E cut by *Bsp*EI
Lane 7	Plasmid pRRS-M3B1 cut by *Bfu*AI	Lane 24	Plasmid pRRS-M10E cut by *Bst*BI
Lane 10	Plasmid pRRS-M4B1 cut by *Bfu*AI	Lane 27	Plasmid pRRS-M11E cut by *Bsp*EI
Lane 11	Plasmid pRRS-M4B2 cut by *Mfe*I	Lane 28	Plasmid pRRS-M11E cut by *Bst*BI
Lane 12	Plasmid pRRS-M4B2 cut by *Bst*BI	Lane 31	Plasmid pRRS-M15G cut by *Sca*I
