# Supplementary material for: Precision Methylome and In Vivo Methylation Kinetics Characterization of Klebsiella pneumoniae
Source: Genomics Proteomics Bioinformatics. 2021 Jun 29;20(2):418–34. doi: 10.1016/j.gpb.2021.04.002 (PMC9684165; doi:10.1016/j.gpb.2021.04.002)
Supplement: Supplementary Figure S10 — Density distribution of the GATC/CCWGG motifs on the 13 K. pneumoniae genomes and random generated genomes The orange histograms show the density distribution of GATC/CCWGG motifs on the randomly generated genomes. The green histograms show the density distribution of GATC/CCWGG on the 13 K. pneumoniae genomes. [file mmc11.pdf]

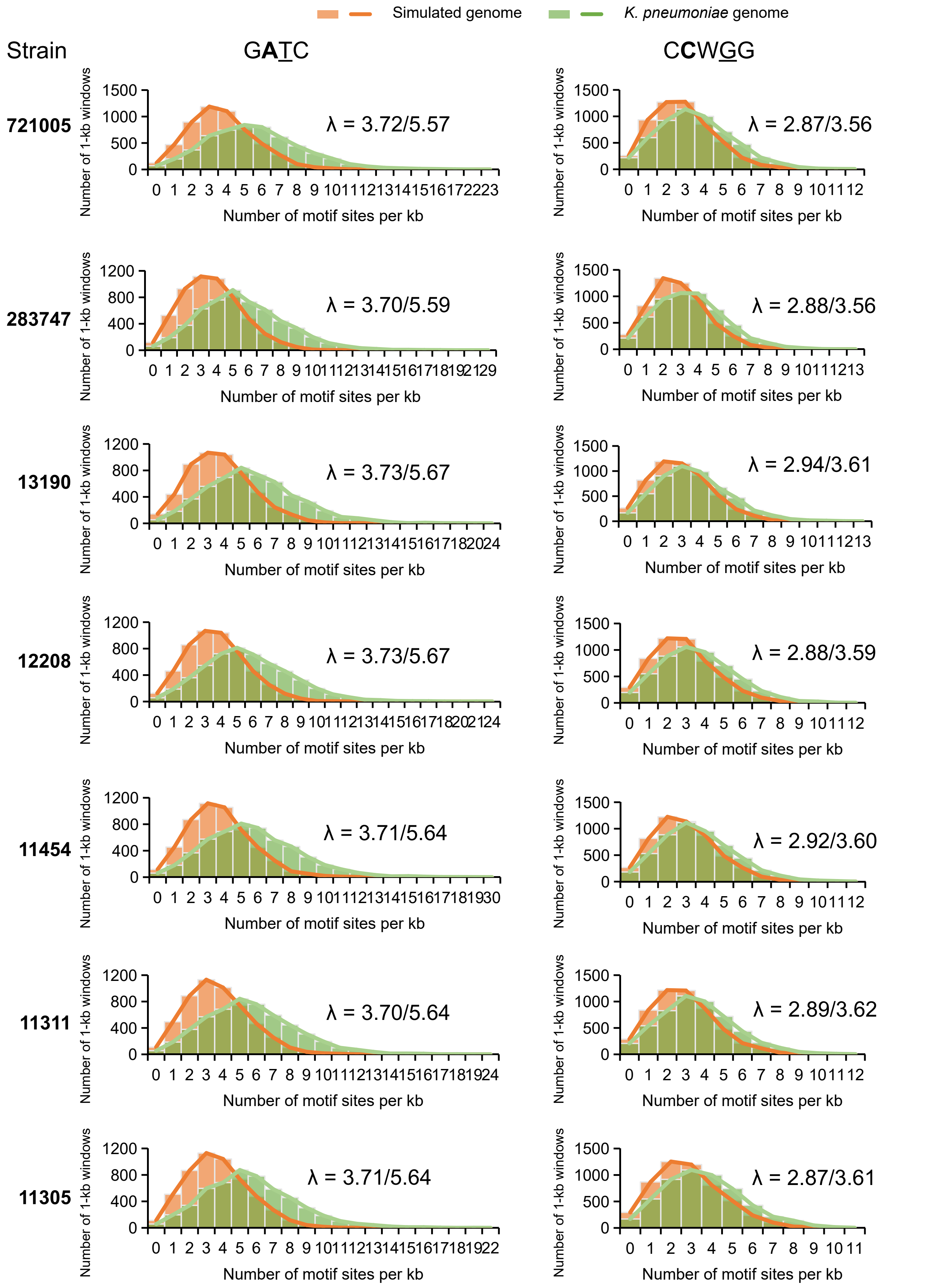

Simulated genome
K. pneumoniae genome
Strain
GATC
CCWGG
721005
λ = 3.72/5.57
λ = 2.87/3.56
283747
λ = 3.70/5.59
λ = 2.88/3.56
13190
λ = 3.73/5.67
λ = 2.94/3.61
12208
λ = 3.73/5.67
λ = 2.88/3.59
11454
λ = 3.71/5.64
λ = 2.92/3.60
11311
λ = 3.70/5.64
λ = 2.89/3.62
11305
λ = 3.71/5.64
λ = 2.87/3.61
Number of 1-kb windows
Number of motif sites per kb

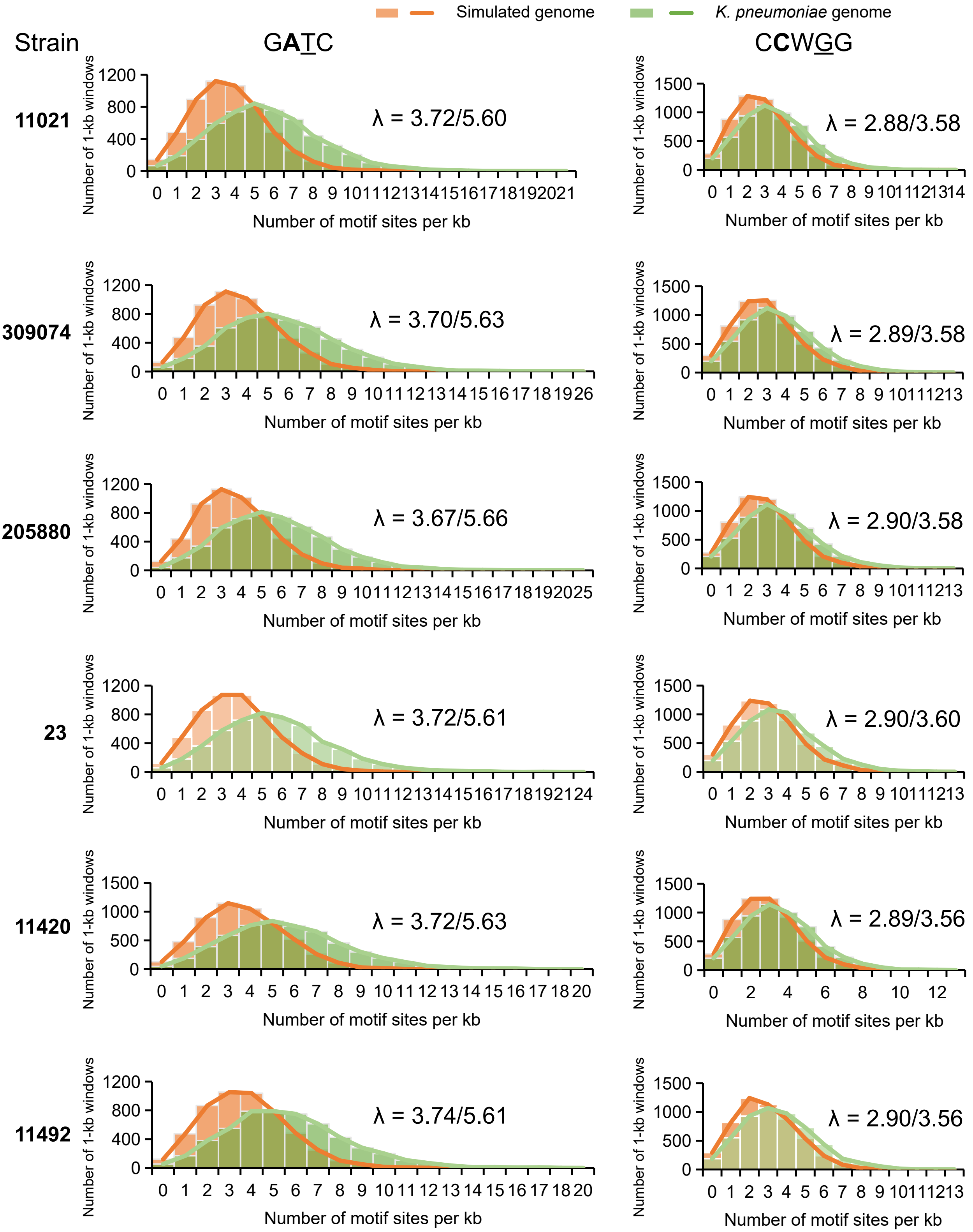

Simulated genome
K. pneumoniae genome
Strain
GATC
CCWGG
11021
309074
205880
23
11420
11492
Number of 1-kb windows
Number of motif sites per kb
λ = 3.72/5.60
λ = 2.88/3.58
λ = 3.70/5.63
λ = 2.89/3.58
λ = 3.67/5.66
λ = 2.90/3.58
λ = 3.72/5.61
λ = 2.90/3.60
λ = 3.72/5.63
λ = 2.89/3.56
λ = 3.74/5.61
λ = 2.90/3.56
