# Supplementary material for: Precision Methylome and In Vivo Methylation Kinetics Characterization of Klebsiella pneumoniae
Source: Genomics Proteomics Bioinformatics. 2021 Jun 29;20(2):418–34. doi: 10.1016/j.gpb.2021.04.002 (PMC9684165; doi:10.1016/j.gpb.2021.04.002)
Supplement: Supplementary Figure S16 — Re-methylation time of GATC motifs in the oriC region (381 nt) of NTUH-K2044 and 11492 The DnaA boxes (blue), AT-rich region (yellow), and GATC motifs (red) are labeled on the sequence. The re-methylation times of GATC motifs in 11492 and NTUH-K2044 stains are marked in purple and green. [file mmc17.pdf]

t_m = 7.6 t_m = 10.3 **DnaA box** t_m = 1.1

11492
5′- GAATTGTAAAGATCGTCCGATCTTCTGTGGATAACCATGCTTAAAAGCTTGGATCAACCG 60
CTTAACATTTCTAGCAGGCTAGAAGACACCTATTGGTACGAATTTTCGAACCTAGTTGGC 60
NTUH-K2044

t_m = 7.6 t_m = 1.3 t_m = 0.4

DnaA box **DnaA box**

11492
GTAGTTATCCAAAGAATAACCGTAGTATGGTTTTTGAGCTGTGCATAACCCTTCATTCTG 120
CATCAATAGGTTTCTTATTGGCATCATACCAAAAACTCGACACGTATTGGGAAGTAAGAC 120
NTUH-K2044

t_m = 3.3 t_m = 3.8 t_m = 12.5 t_m = 28.7

11492
ATCCCAGCTTATACGGACCAGGATCACCGATCATTCACAGATAGTGATCCTTCTTAAGCT 180
TAGGGTCGAATATGCCTGGTCCTAGTGGCTAGTAAGTGTCTATCACTAGGAAGAATTCGA 180
NTUH-K2044

t_m = 0.4 t_m = 0.4 t_m = 23.7 t_m = 24.2

t_m = 19.5 t_m = 21.7 **DnaA box** t_m = 7.0 t_m = 8.1

11492
TTTGATCTTACTTCGCGGATCCGACTTATCCACAAAGAGAGTCGATCCTAATAAGAGATC 240
AAACTAGAATGAAGCGCCTAGGCTGAATAGGTGTTTCTCTCAGCTAGGATTATTCTCTAG 240
NTUH-K2044

t_m = 9.9 t_m = 8.5 t_m = 19.3 t_m = 0.4

AT-rich region

t_m = 9.8 t_m = 13.0 t_m = 3.8

11492
ACAATAGAACAGATCTCTATATAAAGATCTTCTTTTTAATACTCAGGATCCCGGAGTCTT 300
TGTTATCTTGTCTAGAGATATATTTCTAGAAGAAAAATTATGAGTCCTAGGGCCTCAGAA 300
NTUH-K2044

t_m = 0.4 t_m = 24.6 t_m = 18.4

11492
TCTCGAAAGACGAAAGTTGAGTAGAATCCACGGCCCGGGCTTCAATCCATTTTCATACCG 360
AGAGCTTTCTGCTTTCAACTCATCTTAGGTGCCGGGCCCGAAGTTAGGTAAAAGTATGGC 360
NTUH-K2044

11492
CTTTATGCGAGGCAGACCACC -3′ 381
GAAATACGCTCCGTCTGGTGG 381
NTUH-K2044
