# Supplementary material for: Precision Methylome and In Vivo Methylation Kinetics Characterization of Klebsiella pneumoniae
Source: Genomics Proteomics Bioinformatics. 2021 Jun 29;20(2):418–34. doi: 10.1016/j.gpb.2021.04.002 (PMC9684165; doi:10.1016/j.gpb.2021.04.002)
Supplement: Supplementary Figure S18 — COG distributions of genes with hemi/un-methylated GATC/CCWGG motifs in intergenic regions Box plots indicated the COG categories of coding genes with hemi/un-methylated CCWGG (A) and GATC (B) motifs in intergenic regions. X-axis shows the functional classes. Y-axis shows the ratio of genes in each functional class. [A] RNA processing and modification, [B] Chromatin structure and dynamics, [C] Energy production and conversion, [D] Cell cycle control, cell division, chromosome partitioning, [E] Amino acid transport and metabolism, [F] Nucleotide transport and metabolism, [G] Carbohydrate transport and metabolism, [H] Coenzyme transport and metabolism, [I] Lipid transport and metabolism, [J] Translation, ribosomal structure and biogenesis, [K] Transcription, [L] Replication, recombination and repair, [M] Cell wall/membrane/envelope biogenesis, [N] Cell motility, [O] Post-translational modification, protein turnover, and chaperones, [P] Inorganic ion transport and metabolism, [Q] Secondary metabolites biosynthesis, transport, and catabolism, [R] General function prediction only, [S] Function unknown, [T] Signal transduction mechanisms, [U] Intracellular trafficking, secretion, and vesicular transport, [V] Defense mechanisms. [file mmc19.pdf]

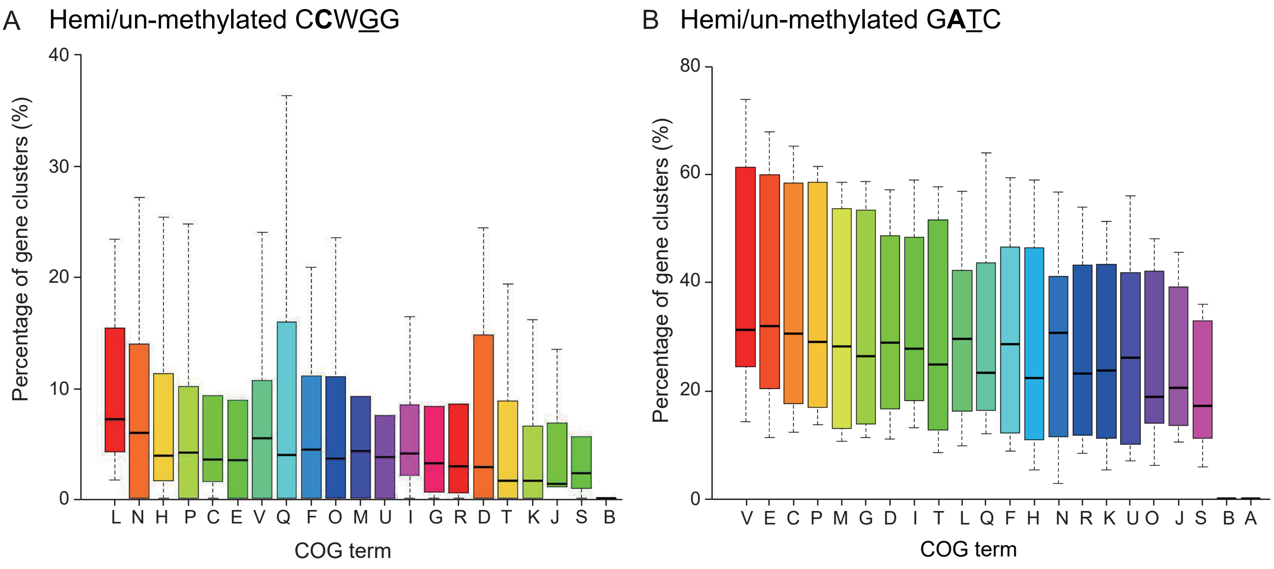

A RNA processing and modification
B Chromatin structure and dynamics
C Energy production and conversion
D Cell cycle control, cell division, chromosome partitioning
E Amino acid transport and metabolism
F Nucleotide transport and metabolism
G Carbohydrate transport and metabolism
H Coenzyme transport and metabolism
I Lipid transport and metabolism
J Translation, ribosomal structure and biogenesis
K Transcription
L Replication, recombination and repair
M Cell wall/membrane/envelope biogenesis
N Cell motility
O Posttranslational modification, protein turnover, chaperones
P Inorganic ion transport and metabolism
Q Secondary metabolites biosynthesis, transport and catabolism
R General function prediction only
S Function unknown
T Signal transduction mechanisms
U Intracellular trafficking, secretion, and vesicular transport
V Defense mechanisms
