# Supplementary material for: Precision Methylome and In Vivo Methylation Kinetics Characterization of Klebsiella pneumoniae
Source: Genomics Proteomics Bioinformatics. 2021 Jun 29;20(2):418–34. doi: 10.1016/j.gpb.2021.04.002 (PMC9684165; doi:10.1016/j.gpb.2021.04.002)
Supplement: Supplementary Table S1 — Clinical information of 14 K. pneumoniae isolates [file mmc21.doc]

**Table S1**  **Clinical information of 14 *K. pneumoniae*** isolates

| **Strain name** | **Samples** | **Capsular**  **type** | **MLST** | **Clonal group (CG)** | **Strains string test** | **Drug** [**sensibility**](../../../../C:/Program%20Files%20(x86)/Youdao/Dict/7.3.0.0817/resultui/dict/%3Fkeyword=sensibility)**^a^** | | | | |
| --- | --- | --- | --- | --- | --- | --- | --- | --- | --- | --- |
|  |  |  |  |  |  | **CFZ** | **CAZ** | **IPM** | **CIP** | **AK** |
| NTUH-K2044 | abscess/meningitis | K1 | 23 | 23 | + | I | S | I | S | S |
| 11492 | blood/abscess | K1 | 23 | 23 | + | R | R | R | R | S |
| 11420 | [ascites](../../../../C:/Program%20Files%20(x86)/Youdao/Dict/7.3.0.0817/resultui/dict/%3Fkeyword=ascites)/sputum | K1 | 1265 | 23 | + | R | R | R | S | S |
| 11454 | blood/abscess | K2 | 86 | / | + | S | S | S | S | S |
| 12208 | sputum | K54 | 4 | 29 | - | R | R | R | S | S |
| 11311 | blood/abscess | K57 | 412 | / | + | S | S | S | S | S |
| 23 | blood/abscess | K57 | 412 | / | + | S | S | S | S | S |
| 11305 | blood/cervical secretion | K64 | 38 | 147 | + | R | R | R | R | S |
| N201205880 | sputum | K21 | 86 | / | - | R | R | R | R | R |
| 309074 | sputum | K24 | 10 | / | - | R | R | R | R | R |
| 13190 | sputum | K27 | 40 | 147 | - | R | R | R | R | R |
| 283747 | sputum | K47 | 4 | 258 | - | R | R | R | R | R |
| 721005 | [urine](../../../../C:/Program%20Files%20(x86)/Youdao/Dict/7.3.0.0817/resultui/dict/%3Fkeyword=urine) | K47 | 4 | 258 | - | R | R | R | R | R |
| 11021 | [urine](../../../../C:/Program%20Files%20(x86)/Youdao/Dict/7.3.0.0817/resultui/dict/%3Fkeyword=urine) | K47 | 4 | 258 | - | R | R | R | R | R |

*Note*: ^a^ CFZ, cefazolin; CAZ, ceftazidime; IPM, [imipenem](../../../../C:/Program%20Files%20(x86)/Youdao/Dict/7.3.0.0817/resultui/dict/%3Fkeyword=imipenem); CIP, [ciprofloxacin](../../../../C:/Program%20Files%20(x86)/Youdao/Dict/7.3.0.0817/resultui/dict/javascript:%3B); AK, [amikacin](../../../../C:/Program%20Files%20(x86)/Youdao/Dict/7.3.0.0817/resultui/dict/%3Fkeyword=amikacin); S, susceptible; R, resistant; I, intermediate.
