# Supplementary material for: Precision Methylome and In Vivo Methylation Kinetics Characterization of Klebsiella pneumoniae
Source: Genomics Proteomics Bioinformatics. 2021 Jun 29;20(2):418–34. doi: 10.1016/j.gpb.2021.04.002 (PMC9684165; doi:10.1016/j.gpb.2021.04.002)
Supplement: Supplementary Table S2 — Sequencing data of the 14 K. pneumoniae strains using SMRT technology [file mmc22.doc]

**Table S2 Sequencing data of the 14 *K. pneumoniae*** strains using SMRT technology

| **Sample** | **Cell** | **No. of bases (bp)** | **Mean read length (bp)** | **Mean subread length (bp)** | **Coverage** |
| --- | --- | --- | --- | --- | --- |
| NTUH-K2044 | 1 | 886,769,768 | 15,421 | 12,257 | 117× |
| 11492 | 2 | 952,838,022 | 8164 | 7142 | 165× |
| 11420 | 1 | 541,500,849 | 10,787 | 3374 | 80× |
| 11454 | 2 | 773,967,122 | 8457 | 2732 | 95× |
| 12208 | 1 | 892,071,700 | 13,096 | 7507 | 134× |
| 11311 | 1 | 891,654,097 | 11,116 | 7490 | 124× |
| 23 | 2 | 737,867,481 | 7954 | 2862 | 82× |
| 11305 | 1 | 666,166,026 | 9720 | 7355 | 103× |
| N201205880 | 1 | 615,336,756 | 14,233 | 7527 | 60× |
| 309074 | 1 | 1,101,489,716 | 12,287 | 8500 | 160× |
| 13190 | 1 | 952,259,010 | 14,438 | 9004 | 67× |
| 283747 | 1 | 623,152,844 | 14,379 | 8926 | 95× |
| 721005 | 1 | 1,152,468,329 | 12,851 | 8130 | 151× |
| 11021 | 1 | 522,202,437 | 14,804 | 7477 | 55× |
