# Supplementary material for: Precision Methylome and In Vivo Methylation Kinetics Characterization of Klebsiella pneumoniae
Source: Genomics Proteomics Bioinformatics. 2021 Jun 29;20(2):418–34. doi: 10.1016/j.gpb.2021.04.002 (PMC9684165; doi:10.1016/j.gpb.2021.04.002)
Supplement: Supplementary Table S3 — Sequencing data of the 14 K. pneumoniae strains using Illumina Hiseq platform [file mmc23.doc]

## Table S3 Sequencing data of the 14 *K. pneumoniae* strains using Illumina Hiseq platform

| **Sample** | **Clean**  **reads** | **Duplication**  **deleted** | **Filter** | **Mate paired** | **Mapped** | **Unique mapped** | **Depth** | **Coverage**  **(10×)** |
| --- | --- | --- | --- | --- | --- | --- | --- | --- |
| NTUH-K2044 | 22,870,446 | 20,436,158 | 19,919,947 | 19,425,742 | 18,118,021 | 17,934,586 | 493 | 99.41% |
| 11492 | 9,548,040 | 8,274,524 | 8,203,572 | 8,140,190 | 7,962,700 | 7,874,315 | 218 | 98.96% |
| 11420 | 8,535,078 | 8,520,932 | 8,247,425 | 8,044,246 | 7,849,334 | 7,753,861 | 168 | 99.22% |
| 11454 | 15,106,774 | 15,037,602 | 14,464,415 | 13,990,144 | 13,921,432 | 13,755,535 | 317 | 99.20% |
| 12208 | 5,809,476 | 5,734,298 | 5,476,698 | 4,906,182 | 4,906,182 | 4,719,438 | 126 | 99.89% |
| 11311 | 7,722,008 | 6,170,366 | 6,063,244 | 5,960,572 | 5,858,494 | 5,797,267 | 160 | 99.21% |
| 23 | 8,726,760 | 8,706,494 | 8,385,358 | 8,135,502 | 8,024,562 | 7,935,682 | 177 | 99.19% |
| 11305 | 9,133,612 | 7,855,376 | 7,789,872 | 7,730,848 | 7,315,970 | 7,224,924 | 202 | 99.04% |
| N201205880 | 7,448,260 | 7,334,122 | 7,277,008 | 7,128,778 | 7,128,718 | 6,762,212 | 243 | 99.99% |
| 309074 | 10,483,754 | 9,279,180 | 9,253,276 | 9,004,180 | 9,004,180 | 6,745,943 | 286 | 95.31% |
| 13190 | 8,748,960 | 8,589,348 | 8,121,571 | 7,853,518 | 5,482,369 | 5,369,274 | 144 | 98.45% |
| 283747 | 10,371,170 | 10,225,530 | 9,890,486 | 9,312,452 | 9,312,452 | 8,885,307 | 239 | 99.99% |
| 721005 | 6,790,294 | 6,739,216 | 6,715,998 | 6,715,998 | 6,482,828 | 6,147,004 | 245 | 99.05% |
| 11021 | 7,191,852 | 7,102,130 | 7,045,938 | 6,902,228 | 6,902,228 | 6,592,975 | 246 | 99.99% |
