# Supplementary material for: Precision Methylome and In Vivo Methylation Kinetics Characterization of Klebsiella pneumoniae
Source: Genomics Proteomics Bioinformatics. 2021 Jun 29;20(2):418–34. doi: 10.1016/j.gpb.2021.04.002 (PMC9684165; doi:10.1016/j.gpb.2021.04.002)
Supplement: Supplementary Table S5 — Bisulfite sequencing data of the 14 K. pneumoniae strains [file mmc25.doc]

## Table S5 Bisulfite sequencing data of the 14 *K. pneumoniae* strains

| **Strain name** | **Clean pair reads^a^** | **Genome depth (×)** | **BS-con rate^b^** | **Number of C**  **(≥ 20×)** | **C covered^c^**  **(≥ 20×)** | **Number of methylated C^d^ (≥ 20×)** |
| --- | --- | --- | --- | --- | --- | --- |
| NTUH-K2044 | 5,120,161 | 190 | 99.96% | 1,558,949 | 99.37% | 19,489/19,457 |
| 11492 | 7,816,589 | 309 | 99.88% | 1,542,407 | 99.10% | 21,570/21,574 |
| 11420 | 4,961,543 | 184 | 99.97% | 1,628,192 | 99.24% | 19,531/19,524 |
| 11454 | 6,801,540 | 263 | 99.45% | 1,545,707 | 99.17% | 16,146/16,195 |
| 12208 | 6,329,978 | 213 | 99.99% | 1,613,576 | 98.70% | 19,903/19,884 |
| 11311 | 9,262,793 | 389 | 99.94% | 1,557,774 | 99.27% | 19,283/19,276 |
| 23 | 3,902,961 | 146 | 99.99% | 1,586,682 | 99.27% | 19,365/19,382 |
| 11305 | 4,704,639 | 177 | 99.92% | 1,532,062 | 99.07% | 19,096/19,102 |
| N201205880 | 8,519,543 | 270 | 100% | 1,707,387 | 98.79% | 20,891/20,875 |
| 309074 | 8,439,345 | 354 | 99.97% | 1,594,371 | 99.19% | 18,185/18,207 |
| 13190 | 6,286,874 | 269 | 99.72% | 1,585,130 | 98.08% | 19,694/19,635 |
| 283747 | 6,093,694 | 190 | 99.90% | 1,597,379 | 98.68% | 19,431/19,424 |
| 721005 | 8,553,164 | 244 | 99.60% | 1,665,462 | 98.46% | 20,578/20,541 |
| 11021 | 6,137,777 | 197 | 99.98% | 1,644,827 | 98.48% | 19,059/19,003 |

*Note*: ^a^ “Clean pair reads” represents the paired reads mapped uniquely to the reference genome by Bismark. ^b^ “BS-con Rate” indicates the bisulfite conversion rate. ^c^ “C covered” indicates the proportion of mapped C sites (≥ 20**×**) over total C sites in the reference genome. ^d^ The number of methylated C on the plus and minus strands of chromosomes and plasimds.
