# Supplementary material for: Precision Methylome and In Vivo Methylation Kinetics Characterization of Klebsiella pneumoniae
Source: Genomics Proteomics Bioinformatics. 2021 Jun 29;20(2):418–34. doi: 10.1016/j.gpb.2021.04.002 (PMC9684165; doi:10.1016/j.gpb.2021.04.002)
Supplement: Supplementary Table S6 — Detailed information of the motifs and corresponding DNA MTases among the 14 K. pneumoniae strains [file mmc26.doc]

## Table S6 Detailed information of the motifs and corresponding DNA MTases among the 14 *K. pneumoniae* strains

| **Strain name** | **Sequence motif ^a^** | **MTase** | **Modification** | **Type^b^** | **Locus**  **M gene** | **Locus**  **S gene** | **Comments ^c^** |
| --- | --- | --- | --- | --- | --- | --- | --- |
| NTUH-K2044 | G**A**TC | M.KpnK2044I (Dam) | 6mA | orphan | peg364 | - | 100% aa identity to M.Kpn43816Dam |
|  | C**C**WGG | M.KpnK2044II (Dcm) | 5mC | orphan | peg1760 | - | 100% aa identity to M.Kpn62629II |
|  | GR**A**CRAC | M.KpnK2044III (KamC) | 6mA | RM-II | peg4432 | - | New |
|  | MT**C**GAK | M.KpnK2044IV (KcmA) | 5mC | RM-II | peg4434 | - | New |
| 11492 | G**A**TC | M.Kpn11492I (Dam) | 6mA | orphan | peg365 | - | 100% aa identity to M.Kpn43816Dam |
|  | C**C**WGG | M.Kpn11492II (Dcm) | 5mC | orphan | peg1825 | - | 100% aa identity to M.Kpn62629II |
|  | MT**C**GAK | M.Kpn11492III (KcmA) | 5mC | RM-II | peg4298 | - | New |
| 11420 | G**A**TC | M.Kpn11420I (Dam) | 6mA | orphan | peg366 | - | 100% aa identity to M.Kpn43816Dam |
|  | C**C**WGG | M.Kpn11420II (Dcm) | 5mC | orphan | peg1936 | - | 100% aa identity to M.Kpn62629II |
|  | RT**A**CN_5_GGC | M1.Kpn11420III  M2.Kpn11420III (KamA) | 6mA | RM-I | peg4701 peg4700 | peg4699 | New |
|  | TTC**A**N_7_TTC | M.Kpn11420IV (KamB) | 6mA | RM-I | peg5619 | peg5618 | New |
| 11454 | G**A**TC | M.Kpn11454I (Dam) | 6mA | orphan | peg356 | - | 100% aa identity to M.Kpn43816Dam |
|  | C**C**WGG | M.Kpn11454 (Dcm) | 5mC | orphan | peg1716 | - | 100% aa identity to M.Kpn62629II |
|  | **A**GCN_5_CTTC | M.Kpn11454III | 6mA | ? | peg1670 | peg1671 | 99.9% aa identity to M.KpnGH01II |
| 12208 | G**A**TC | M.Kpn11208I (Dam) | 6mA | orphan | peg379 | - | 100% aa identity to M.Kpn43816Dam |
|  | C**C**WGG | M.Kpn11208II (Dcm) | 5mC | orphan | peg1747 | - | 100% aa identity to M.Kpn62629II |
|  | AGGA**A**G | M.Kpn12208III (KamD) | 6mA | RM-II | peg4487 | - | New |
| 11311 | G**A**TC | M.Kpn11311I (Dam) | 6mA | orphan | peg358 | - | 100% aa identity to M.Kpn43816Dam |
|  | C**C**WGG | M.Kpn11311II (Dcm) | 5mC | orphan | peg1742 | - | 100% aa identity to M.Kpn62629II |
|  | CC**A**YN_7_TTYG | M.Kpn11311III (KamE) | 6mA | RM-I | peg4473 | peg4474 | New |
| 23 | G**A**TC | M.Kpn23I (Dam) | 6mA | orphan | peg361 | - | 100% aa identity to M.Kpn43816Dam |
|  | C**C**WGG | M.Kpn23II (Dcm) | 5mC | orphan | peg1802 | - | 100% aa identity to M.Kpn62629II |
|  | CC**A**YN_7_TTYG | M.Kpn23III (KamE) | 6mA | RM-I | peg4539 | peg4540 | New |
| 11305 | G**A**TC | M.Kpn11305I (Dam) | 6mA | orphan | peg365 | - | 100% aa identity to M.Kpn43816Dam |
|  | C**C**WGG | M.Kpn11305II (Dcm) | 5mC | orphan | peg1826 | - | 100% aa identity to M.Kpn62629II |
|  | CC**A**GN_7_RTTC | M.Kpn11305III | 6mA | RM-I | peg4529 | peg4530 | 100% aa identity to M.KpnAATI |
| N201205880 | G**A**TC | M.Kpn05880I (Dam) | 6mA | orphan | peg363 | - | 100% aa identity to M.Kpn43816Dam |
|  | C**C**WGG | M.Kpn05880II (Dcm) | 5mC | orphan | peg1726 | - | 100% aa identity to M.Kpn62629II |
|  | CC**A**N_7_TCAC | M.Kpn05880III (KamG) | 6mA | RM-I | peg5335 | peg5336 | New |
| 309074 | G**A**TC | M.Kpn309074I (Dam) | 6mA | orphan | peg363 | - | 100% aa identity to M.Kpn43816Dam |
|  | C**C**WGG | M.Kpn309074II (Dcm) | 5mC | orphan | peg1868 | - | 100% aa identity to M.Kpn62629II |
|  | CATCN_6_TTYG | M.Kpn309074III | 6mA | RM-I | peg650 | peg649 | 100% aa identity to M.Kpn39795II |
|  | CTAN_5_GTAA | M.Kpn309074IV | 6mA | RM-I | peg4560 | peg4561 | 99.8% aa identity to M.Kpn35657I |
|  | CAGN_6_TCAA | M.Kpn309074V (KamH) | 6mA | RM-I | peg4841 | peg4840 | New |
| 13190 | G**A**TC | M.Kpn13190I (Dam) | 6mA | orphan | peg374 | - | 100% aa identity to M.Kpn43816Dam |
|  | C**C**WGG | M.Kpn13190II (Dcm) | 5mC | orphan | peg1805 | - | 100% aa identity to M.Kpn62629II |
|  | CC**A**GN_7_RTTC | M.Kpn13190III | 6mA | RM-I | peg4519 | peg4520 | 100% aa identity to M.KpnAATI |
|  | GGC**A**N_8_TCG | M.Kpn13190IV | 6mA | RM-I | peg4583 | peg4582 | 100% aa identity to M.KpnAATIV |
| 283747 | G**A**TC | M.Kpn283747I (Dam) | 6mA | orphan | peg368 | - | 100% aa identity to M.Kpn43816Dam |
|  | C**C**WGG | M.Kpn283747II (Dcm) | 5mC | orphan | peg1927 | - | 100% aa identity to M.Kpn62629II |
| 721005 | G**A**TC | M.Kpn721005I (Dam) | 6mA | orphan | peg375 | - | 100% aa identity to M.Kpn43816Dam |
|  | C**C**WGG | M.Kpn721005II (Dcm) | 5mC | orphan | peg1933 | - | 100% aa identity to M.Kpn62629II |
| 11021 | G**A**TC | M.Kpn11021I (Dam) | 6mA | orphan | peg372 | - | 100% aa identity to M.Kpn43816Dam |
|  | C**C**WGG | M.Kpn11021II (Dcm) | 5mC | orphan | peg1937 | - | 100% aa identity to M.Kpn62629II |

*Note*: ^a^ The methylated nucleotide in the motif is shown as bold letter. The underlined letter represents the guanine pairing with the methylated cytosine on the complementary strand. Degenerate bases used in our recognition sequences are listed in the following: R = G or A, Y = C or T, M = A or C, K = G or T, S = G or C, W = A or T, B = not A (C or G or T), D = not C (A or G or T), H = not G (A or C or T), V = not T (A or C or G), N = A or C or G or T; ^b^ The MTase prediction was based on the sequence alignment with REBASE database (http://rebase.neb.com/rebase/rebase.html); The predicted MTases were further classified as Type I, Type II, or orphan MTases according to the annotation information. ^c^ Some predicted MTases showed ~ 99.8%–100% identities with the known MTases as previously reported. “New” indicates the eight newly identified methylation motifs and corresponding MTases in our study.
