# Supplementary material for: Precision Methylome and In Vivo Methylation Kinetics Characterization of Klebsiella pneumoniae
Source: Genomics Proteomics Bioinformatics. 2021 Jun 29;20(2):418–34. doi: 10.1016/j.gpb.2021.04.002 (PMC9684165; doi:10.1016/j.gpb.2021.04.002)
Supplement: Supplementary Table S7 — Fishers’ exact test of the un-methylated and methylated MTCGAK sites [file mmc27.doc]

## Table S7 Fisher’s exact test of the un-methylated and methylated MTCGAK sites

| **Strain name** | **Sequence** | **Methylated motif** | **Un-methylated motif** | **Fisher’s exact test** | **Total motif** |
| --- | --- | --- | --- | --- | --- |
| 11492 | GMT**C**GAK | 476 | 1092 | *P* < 0.001 | 1574 |
|  | HMT**C**GAK | 2636 | 709 |  | 3400 |
| NTUH-K2044 | GMT**C**GAK | 349 | 1107 | *P* < 0.001 | 1546 |
|  | HMT**C**GAK | 1840 | 711 |  | 2551 |

*Note*: Degenerate bases used in the recognition sequences are listed in the following: M = A or C, K = G or T, H = not G (A or C or T).
