# Supplementary material for: Precision Methylome and In Vivo Methylation Kinetics Characterization of Klebsiella pneumoniae
Source: Genomics Proteomics Bioinformatics. 2021 Jun 29;20(2):418–34. doi: 10.1016/j.gpb.2021.04.002 (PMC9684165; doi:10.1016/j.gpb.2021.04.002)
Supplement: Supplementary Table S8 — 22 predicted MTase genes and the corresponding 15 methylation motifs [file mmc28.doc]

## Table S8 22 predicted MTase genes and the corresponding 15 methylation motifs

| 1. **M Type** | **Candidate MTase genes** | | **Strain** | **Motif ^a^** | | **REBASE ^b^** |
| --- | --- | --- | --- | --- | --- | --- |
| **Type II** | **K2044peg364/11492peg365/11420peg366/**  **11454peg356/12208/peg379/11311peg358/23peg361/**  **11305peg365/N201005880peg363/309074peg363/**  **13190peg374/283747peg368/721005peg375/**  **11021peg372** | **M1A** | **14 strains** | **GATC** | A | 100% aa identity to M.Kpn43816Dam |
| **Type I** | **11420peg4701.peg4700.peg4699** | **M2B1** | **11420** | **RTACN_5_GGC** | B1 | **New** |
| Type I | M/S.Eco448ORF25820P | M3B1 |  |  |  |  |
| Type II | M.KpnSWU01ORFJP | M4B1 |  |  |  |  |
| Type II | M.Sen39523DndAP | M5B1 |  |  |  |  |
| Type I | M1/M2/S.KpnHSL4ORFAP | M2B2 |  | **TTCAN_7_TTC** | B2 | **New** |
| **Type I** | **11420peg5619.peg5618** | **M3B2** |  |  |  |  |
| Type II | M.KpnSWU01ORFJP | M4B2 |  |  |  |  |
| Type II | M.Sen39523DndAP | M5B2 |  |  |  |  |
| **Type I** | **11305peg4529.peg4530** | **M6C** | **11305** | **CCAGN_7_RTTC** | C | 100% aa identity to M.KpnAATI |
| **Type I** | **23peg4539.peg4540/11311peg4473.peg4474** | **M7D** | **23/11311** | **CCAYN_7_TTYG** | D | **New** |
| Type II | M.Sen39523DndAP | M8D |  |  |  |  |
| **Type I** | **11454peg1670.peg1671** | **M9E** | **11454** | **AGCN_5_CTTC** | E | 99.9% aa identity to M.KpnGH01II |
| Type III | M.Kpn214ORFGP | M10E |  |  |  |  |
| Type II | M.Sen39523DndAP | M11E |  |  |  |  |
| **Type I** | **13190peg4519.peg4520** | **M12** | **13190** | **CCAGN_7_RTTC** | C | 100% aa identity to M.KpnAATI |
| **Type I** | **13190peg4583.peg4582** | **M13** |  | **GGCAN_8_TCG** | F | 100% aa identity to M.KpnAATIV |
| **Type II** | **12208peg4487** | **M14** | **12208** | **AGGAAG** | G | **New** |
| Type II | M.Sen39523DndAP | M15 |  |  |  |  |
| **Type I** | **N201205880peg5335.peg5336** | **M17** | **N201205880** | **CCAN_7_TCAC** | I | **New** |
| **Type I** | **309074peg650.peg649** | **M18** | **309074** | **CATCN_6_TTYG** | J | 100% aa identity to M.Kpn39795II |
| **Type I** | **309074peg4560.peg4561** | **M20** |  | **CTAN_5_GTAA** | M | 99.8% aa identity to M.Kpn35657I |
| **Type I** | **309074peg4841.peg4840** | **M21** |  | **CAGN_6_TCAA** | N | **New** |
| **Type II** | **K2044peg4432** | **M24** | **NTUH-K2044** | **GRACRAC** | P | **New** |
| **Type II** | **K2044peg4434/11492peg4298** | **M27** | **NTUH-K2044**  **/11492** | **MTCGAK** | R | **New** |
| **Type II** | **K2044peg1760/11492peg1825/11420peg1936/**  **11454peg1716/12208peg1747/11311peg1742/**  **23peg1802/11305peg1826/N201205880peg1726/**  **309074peg1868/13190peg1805/283747peg1927/**  **721005peg1933/11021peg1937** | **M32** | **14 strains** | **CCWGG** | V | 100% aa identity to M.Kpn62629II |

*Note*: ^a^ The methylated nucleotide in the motif is shown as bold letter. The underlined letter represents the guanine pairing with the methylated cytosine on the complementary strand. Degenerate bases used in our recognition sequences are listed in the following: R = G or A, Y = C or T, M = A or C, K = G or T, S = G or C, W = A or T, B = not A (C or G or T), D = not C (A or G or T), H = not G (A or C or T), V = not T (A or C or G), N = A or C or G or T; ^b^ The MTase prediction was based on the sequence alignment with REBASE database (http://rebase.neb.com/rebase/rebase.html); The predicted MTases were further classified as Type I, Type II, or orphan MTases according to the annotation information. Some predicted MTases showed ~ 99.8%–100% identities with the known MTases as previously reported. “New” indicates the eight newly identified methylation motifs and corresponding MTases in our study.
