# Supplementary material for: Precision Methylome and In Vivo Methylation Kinetics Characterization of Klebsiella pneumoniae
Source: Genomics Proteomics Bioinformatics. 2021 Jun 29;20(2):418–34. doi: 10.1016/j.gpb.2021.04.002 (PMC9684165; doi:10.1016/j.gpb.2021.04.002)
Supplement: Supplementary Table S10 — Distribution of CCWGG motifs in GRs and IGRs among 14 K. pneumoniae strains [file mmc30.doc]

## Table S9 Distribution of GATC motifs in GRs and IGRs among 14 *K. pneumoniae* strains

| **Strains No.** | **Methylated sites (GATC)** | | | | **Hemi-methylated sites (GATC)** | | | | **Un-methylated sites (GATC)** | | | |
| --- | --- | --- | --- | --- | --- | --- | --- | --- | --- | --- | --- | --- |
|  | **Total** | **GR** | **IGR** | **IGR (%)** | **Total** | **GR** | **IGR** | **IGR (%)** | **Total** | **GR** | **IGR** | **IGR (%)** |
| 11492 | 30,271 | 28,622 | 1649 | 5.45 | 31 | 26 | 5 | 16.13 | 14 | 3 | 11 | 78.57 |
| 11420 | 26,835 | 25,440 | 1395 | 5.20 | 4669 | 4376 | 293 | 6.28 | 343 | 292 | 51 | 14.87 |
| NTUH-K2044 | 30,151 | 28,861 | 1290 | 4.28 | 551 | 507 | 44 | 7.99 | 25 | 9 | 16 | 64.00 |
| 11454 | 28,046 | 26,620 | 1426 | 5.08 | 2142 | 1989 | 153 | 7.14 | 90 | 61 | 29 | 32.22 |
| 12208 | 30,695 | 28,993 | 1702 | 5.54 | 790 | 716 | 74 | 9.37 | 27 | 10 | 17 | 62.96 |
| 11311 | 29,520 | 27,967 | 1553 | 5.26 | 1076 | 999 | 77 | 7.16 | 27 | 15 | 12 | 44.44 |
| 23 | 26,523 | 25,120 | 1403 | 5.29 | 4423 | 4181 | 242 | 5.47 | 329 | 279 | 50 | 15.20 |
| N201205880 | 28,007 | 26,322 | 1685 | 6.02 | 4852 | 4574 | 278 | 5.73 | 312 | 267 | 45 | 14.42 |
| 11305 | 29,222 | 27,668 | 1554 | 5.32 | 1122 | 1033 | 89 | 7.93 | 25 | 16 | 9 | 36.00 |
| 721005 | 31,521 | 30,122 | 1399 | 4.44 | 687 | 638 | 49 | 7.13 | 34 | 13 | 21 | 61.76 |
| 11021 | 27,907 | 26,435 | 1472 | 5.27 | 3968 | 3744 | 224 | 5.65 | 205 | 168 | 37 | 18.05 |
| 309074 | 30,384 | 28,841 | 1543 | 5.08 | 782 | 722 | 60 | 7.67 | 42 | 17 | 25 | 59.52 |
| 13190 | 28,766 | 27,199 | 1567 | 5.45 | 2515 | 2310 | 205 | 8.15 | 138 | 99 | 39 | 28.26 |
| 283747 | 30,156 | 28,855 | 1301 | 4.31 | 1126 | 1046 | 80 | 7.10 | 53 | 24 | 29 | 54.72 |
