# Supplementary material for: Precision Methylome and In Vivo Methylation Kinetics Characterization of Klebsiella pneumoniae
Source: Genomics Proteomics Bioinformatics. 2021 Jun 29;20(2):418–34. doi: 10.1016/j.gpb.2021.04.002 (PMC9684165; doi:10.1016/j.gpb.2021.04.002)
Supplement: Supplementary Table S11 — Summary of genes with upstream hemi/un-methylated GATC sites shared in the 14 K. pneumoniae strains [file mmc31.doc]

## Table S11 Summary of the genes with upstream hemi/un-methylated GATC sites shared in the 14 *K. pneumoniae* strains

| **Gene annotation** | **Location^a^** | ***K. pneumoniae* strains^b^** | | | | | | | | | | | | | |
| --- | --- | --- | --- | --- | --- | --- | --- | --- | --- | --- | --- | --- | --- | --- | --- |
|  |  | **K2044** | **11492** | **11420** | **11454** | **12208** | **11311** | **23** | **05880** | **11305** | **21005** | **11021** | **09074** | **13190** | **283747** |
| Hydroxyaromatic non-oxidative decarboxylase protein B | -75bp | **-/-** | **-/-** | **-/-** | **-/-** | **-/-** | **-/-** | **-/-** | **-/-** | **-/+** | **-/+** | **-/-** | **-/-** | **-/-** | **-/-** |
| PTS system, glucitol /sorbitol-specific IIC component | -85bp | **-/-** | **-/-** | **-/-** | **-/-** | **-/-** | **-/-** | **-/-** | **-/-** | **-/-** | **-/-** | **-/-** | **-/-** | **-/-** | **-/-** |
| PTS system, mannitol -specific IIA/B/C component | -157bp | **-/+** | **-/+** | **-/+** | **-/+** | **-/+** | **-/+** | **-/+** | **-/-** | **-/+** | **-/-** | **-/-** | **-/-** | **-/+** | **-/-** |
| Transcriptional regulator of arabinitol utilization, DeoR family | -141bp | **-/-** | **-/-** | **-/-** | **-/-** | **-/-** | **-/-** | **-/-** | **-/-** | **-/-** | **-/-** | **-/-** | **-/-** | **-/-** | **-/-** |
|  | -168bp | **-/+** | **-/-** | **-/-** | **-/-** | **-/-** | **-/-** | **-/-** | **-/-** | **-/-** | **-/-** | **-/-** | **-/-** | **-/-** | **-/-** |
| D-arabinitol 4-dehydrogenase | -41bp | **-/+** | **-/-** | **-/-** | **-/-** | **-/-** | **-/-** | **-/-** | **-/-** | **-/-** | **-/-** | **-/-** | **-/-** | **-/-** | **-/-** |
|  | -68bp | **-/-** | **-/-** | **-/-** | **-/-** | **-/-** | **-/-** | **-/-** | **-/-** | **-/-** | **-/-** | **-/-** | **-/-** | **-/-** | **-/-** |
| Antiholin-like protein LrgA | -61bp | **-/+** | **-/-** | **-/-** | **-/-** | **-/-** | **-/+** | **-/-** | **-/+** | **-/+** | **-/-** | **-/+** | **-/+** | **-/-** | **-/+** |
| C4-type zinc finger protein, DksA/TraR family | -30bp | **-/+** | **-/+** | **-/+** | **-/+** | **-/+** | **-/+** | **-/+** | **-/+** | **-/+** | **-/+** | **-/+** | **-/+** | **-/+** | **-/+** |
| Alpha-galactosidase | -37bp | **-/-** | **-/-** | **-/+** | **-/-** | **-/-** | **-/-** | **-/-** | **-/-** | **-/-** | **-/-** | **-/-** | **-/+** | **-/-** | **-/-** |
| Putative outer membrane protein | -59bp | **-/+** | **-/+** | **-/-** | **-/-** | **-/-** | **-/+** | **-/-** | **-/-** | **-/+** | **-/+** | **-/+** | **-/-** | **-/+** | **-/+** |
| Nudix hydrolase family protein YffH | -63bp | **-/+** | **-/-** | **-/-** | **-/-** | **-/+** | **-/+** | **-/+** | **-/-** | **-/+** | **-/+** | **-/+** | **-/+** | **-/-** | **-/+** |
| FIG00732740: hypothetical protein | -125bp | **-/-** | **-/-** | **-/-** | **-/-** | **-/-** | **-/-** | **-/-** | **-/-** | **-/-** | **-/-** | **-/-** | **-/-** | **-/-** | **-/-** |

*Note*: ^a^ The distance from the hemi/Un-methylated site to the start codon of the downstream gene; ^b^ ‘-/-’ denotes the upstream Un-methylated motifs on both strands. ‘-/+’ indicates the upstream hemi-methylated motif.
