# Supplementary material for: Precision Methylome and In Vivo Methylation Kinetics Characterization of Klebsiella pneumoniae
Source: Genomics Proteomics Bioinformatics. 2021 Jun 29;20(2):418–34. doi: 10.1016/j.gpb.2021.04.002 (PMC9684165; doi:10.1016/j.gpb.2021.04.002)
Supplement: Supplementary Table S12 — Summary of genes with upstream hemi/un-methylated GATC motif clusters in the 14 K. pneumoniae strains [file mmc32.doc]

## Table S12 Summery of the genes with upstream hemi/Un-methylated GATC motif clusters in the 14 *K. pneumoniae* strains^a^

| **Genes** | **Strains^b^** | **Hemi/Un-methylated motifs^a^ (No.)** |
| --- | --- | --- |
| PTS system, mannitol-specific IIC component | 6 | 3–4 |
| tRNA uridine 5-carboxymethylaminomethyl modification enzyme GidA | 5 | 4–5 |
| Glycerol uptake facilitator protein | 4 | 4–6 |
| Leucine-responsive regulatory protein, regulator for leucine (or lrp) regulon and high-affinity branched-chain amino acid transport system | 3 | 3–8 |
| Putative membrane protein precursor | 3 | 3–8 |
| Ferredoxin | 3 | 3–4 |
| Outer membrane protein W precursor | 2 | 3–4 |
| GMP synthase [glutamine-hydrolyzing] | 2 | 4–6 |
| Putative transport protein | 2 | 3–5 |
| Transaldolase | 2 | 5–6 |
| ParB | 2 | 5–6 |
| Glutathione S-transferase, theta | 2 | 3–4 |

*Note*: ^a^ “Upstream hemi/Un-methylated GATC motif cluster” means that an upstream intergenic region contains at least three consecutive hemi/Un-methylated motifs; ^b^ “Strains” represents the strains with the hemi/Un-methylated GATC motif clusters.
