# Supplementary material for: Precision Methylome and In Vivo Methylation Kinetics Characterization of Klebsiella pneumoniae
Source: Genomics Proteomics Bioinformatics. 2021 Jun 29;20(2):418–34. doi: 10.1016/j.gpb.2021.04.002 (PMC9684165; doi:10.1016/j.gpb.2021.04.002)
Supplement: Supplementary Table S13 — SMRT sequencing data of the samples at three growth time points (1, 4, and 24 h) of 11492 and NTUH-K2044 [file mmc33.doc]

## Table S13 SMRT Sequencing data of the samples at three growth time points (1, 4, and 24 h) of 11492 and NTUH-K2044

| **Sample** | **Cell** | **Number of Bases (bp)** | **Mean Read Length (bp)** | **Mean Subread length (bp)** | **Coverage** |
| --- | --- | --- | --- | --- | --- |
| 11492_1h | 1 | 1,463,421,074 | 14,095 | 8209 | 160**×** |
| 11492_4h | 1 | 1,290,931,013 | 12,382 | 9168 | 159**×** |
| 11492_24h | 1 | 1,061,611,880 | 10,129 | 6510 | 132**×** |
| NTUH-K2044_1h | 1 | 1,446,229,850 | 14,195 | 8384 | 177**×** |
| NTUH-K2044_4h | 1 | 681,653,271 | 11,213 | 9378 | 100**×** |
| NTUH-K2044_24h | 1 | 1,446,068,018 | 13,125 | 9085 | 145**×** |
