# Supplementary material for: Precision Methylome and In Vivo Methylation Kinetics Characterization of Klebsiella pneumoniae
Source: Genomics Proteomics Bioinformatics. 2021 Jun 29;20(2):418–34. doi: 10.1016/j.gpb.2021.04.002 (PMC9684165; doi:10.1016/j.gpb.2021.04.002)
Supplement: Supplementary Table S14 — Bisulfite sequencing data of the samples at three growth time points (1, 4, and 24 h) of 11492 and NTUH-K2044 [file mmc34.doc]

## Table S14 Bisulfite sequencing data of the samples at three growth time points (1, 4 and 24 h) of 11492 and NTUH-K2044

| **Sample** | **Clean pair**  **Reads^a^** | **Genome depth** | **BS-con Rate^b^** | **Number of C**  **(≥ 20×)** | **C covered^c^**  **(≥ 20×)** | **Number of methylated**  **C^d^ (≥ 20×)** |
| --- | --- | --- | --- | --- | --- | --- |
| 11492_1h | 53,139,694 | 2706**×** | 99.36% | 1,542,690 | 99.12% | 25,054/24,943 |
| 11492_4h | 40,388,108 | 2061**×** | 99.83% | 1,543,117 | 99.15% | 23,525/23,466 |
| 11492_24h | 45,586,499 | 2299**×** | 98.89% | 1,543,566 | 99.18% | 24,713/24,609 |
| NTUH-K2044_1h | 6,049,372 | 245**×** | 100% | 1,501,581 | 95.59% | 20,435/20,414 |
| NTUH-K2044_4h | 5,266,802 | 223**×** | 100% | 1,498,879 | 95.42% | 16,896/16,752 |
| NTUH-K2044_24h | 83,926,262 | 4168**×** | 98.63% | 1,551,123 | 98.75% | 29,835/29,882 |

*Note*: ^a^ “Clean pair reads” represents the paired reads mapped uniquely to the reference genome by Bismark. ^b^ “BS-con Rate” indicates the bisulfite conversion rate. ^c^ “C covered” indicates the proportion of mapped C sites (≥ 20**×**) over total C sites in the reference genome. ^d^ The number of methylated C on the plus and minus strands of chromosomes and plasimds.
