# Supplementary material for: Precision Methylome and In Vivo Methylation Kinetics Characterization of Klebsiella pneumoniae
Source: Genomics Proteomics Bioinformatics. 2021 Jun 29;20(2):418–34. doi: 10.1016/j.gpb.2021.04.002 (PMC9684165; doi:10.1016/j.gpb.2021.04.002)
Supplement: Supplementary Table S16 — The seven CCWGG sites changed in methylation state during the growth phase of NTUH-K2044 [file mmc36.doc]

**Table S16 The seven CCWGG sites changed in methylation state during the growth phase of NTUH-K2044**

| **Genome position** | **Gene** | **Function** |
| --- | --- | --- |
| 1,768,284 | peg.1709 | Possible transcriptionl regulator |
| 3,136,272 | peg.3082 | Mobile element protein |
| 3,754,070 | peg.3650 | Mobile element protein |
| 454,539 | rna.3 | Small Subunit Ribosomal RNA |
| 454,598 | rna.3 | Small Subunit Ribosomal RNA |
| 455,077 | rna.3 | Small Subunit Ribosomal RNA |
| 4,955,936 | rna.87 | Small Subunit Ribosomal RNA |
