# Supplementary material for: Precision Methylome and In Vivo Methylation Kinetics Characterization of Klebsiella pneumoniae
Source: Genomics Proteomics Bioinformatics. 2021 Jun 29;20(2):418–34. doi: 10.1016/j.gpb.2021.04.002 (PMC9684165; doi:10.1016/j.gpb.2021.04.002)
Supplement: Supplementary Table S18 — Summary of the genes with upstream hemi/un-methylated CCAYN7TTYG sites shared in the 23 and 11311 strains [file mmc38.doc]

## Table S18 Summary of the genes with upstream hemi/un-methylated CCAYN_7_TTYG sites shared in the 23 and 11311 strains

| **Gene annotation** | **Location ^a^** | ***K. pneumoniae* strains ^b^** | |
| --- | --- | --- | --- |
|  |  | **23** | **11311** |
| 6-phospho-beta-glucosidase (EC 3.2.1.86) | -151bp | **-/+** | **-/+** |
| Deoxyribose-phosphate aldolase (EC 4.1.2.4) | -350bp | **-/+** | **-/+** |
| Iron(III) dicitrate transport protein FecA | -10bp | **-/+** | **-/+** |

*Note:* ^a^ The distance from the hemi/Un-methylated site to the start codon of the downstream gene; ^b^ ‘-/-’ denotes the upstream Un-methylated motifs on both strands. ‘-/+’ indicates the upstream hemi-methylated motif.
