# Supplementary material for: Precision Methylome and In Vivo Methylation Kinetics Characterization of Klebsiella pneumoniae
Source: Genomics Proteomics Bioinformatics. 2021 Jun 29;20(2):418–34. doi: 10.1016/j.gpb.2021.04.002 (PMC9684165; doi:10.1016/j.gpb.2021.04.002)
Supplement: Supplementary Table S19 — Oligonucleotide primers used in this study [file mmc39.doc]

## Table S19 Oligonucleotide primers used in this study

| **Primer^a^** | **Motif** | **5’-3’ sequence^b^** | **RE* for cloning** | **Methylation-sensitive RE for verification** |
| --- | --- | --- | --- | --- |
| pRRS_F |  | TCATTAGGCACCCCAGGCT | - | - |
| pRRS_R |  | TTCCCAGTCACGACGTTGTA | - | - |
| M2B1_F | **RTACN_5_GGC** | CG CCTGCAGGTTAAGGTTAATCATATGACACTGATTAACCTAAAAGATCTCG | SbfI |  |
| M2B1_R |  | CG GGATCC**GCCTGCAGGTAT**TTAAACCTTAAAAATTTTTGTTAGCTGC | BamHI | BfuAI |
| M3B2_F | **TTCAN_7_TTC** | CG CCTGCAGGTTAAGGTTAATCATATGGCACAACAGACACACAC | SbfI |  |
| M3B2_R |  | CG GGATCCTTC**GAATTCCAATTGAA**TCAGTTGCTGGCGGTTTCTG | BamHI | MfeI, BstBI |
| M7D_F | **CCAYN_7_TTYG** | CC CGAAGACATAGCTTTAAGGTTAATCATATGGCCGCAATCTCTTTCGA | BbsI |  |
| M7D_R |  | CG CCTGCAGGTT**CGAATTCGCAGGTGG**TCATGCGCTGGCCTCCTGTG | SbfI | BfuAI, BstBI |
| M14G_F | **AGGAAG** | CG CCTGCAGGTTAAGGTTAATCATATGAACACTGCCAATTTAAA | SbfI |  |
| M14G_R |  | CG GGATCCAGTA**CTTCCT**TTAAGCAATAGCTGCTTT | BamHI | ScaI |
| M17I_F | **CCAN_7_TCAC** | CG CCTGCAGGTTAAGGTTAATCATATGAACGACAAAATCAGTC | SbfI |  |
| M17I_R |  | CG GGATCC**GTGAATAGTACTGG**TCATACGGCAACCGCCTCAT | BamHI | ScaI |
| M21N_F | **CAGN_6_TCAA** | CG CCTGCAGGTTAAGGTTAATCATATGGCCATTAAGAAAACCGA | SbfI |  |
| M21N_R |  | CG GGATCC**TTGACGAGTACTG**TCACTCATGAATAACCTCCT | BamHI | ScaI |
| M24P_F | **GRACRAC** | CG CCTGCAGGTTAAGGTTAATCATATGAGTTTAAATAACATTCA | SbfI |  |
| M24P_R |  | CG GGATCCACAT**GTTGTTC**CTAGAGATCGTTAATCC | BamHI | PciI |
| M27R_F | **MTCGAK** | CG CCTGCAGGTTAAGGTTAATCATATGAAAAAAGTATCTTGTGT | SbfI |  |
| M27R_R |  | CG GGATCC**ATCGAT**TCACTTTGTTGCGACCAAGT | BamHI | BspDI |
| M2_F | **RTACN_5_GGC** | CG CCTGCAGG TTAAGGTTAATCATATGACTAATAAGAAAATCA | SbfI |  |
| M2B2_R |  | CG GGATCC **GAATTC CAATTGAA**TTAAACCTTAAAAATTTTTGTTAGCTGC | BamHI | MfeI, EcoRI |
| M3_F | **TTCAN_7_TTC** | CG CCTGCAGG TTAAGGTTAATCATATGGCACAACAGACACACAC | SbfI |  |
| M3B1_R |  | CG GGATCC**GCCTGCAGGTAT**TCAGATCCTTCCACCCACCC | BamHI | BfuAI |
| M4_F | **TTCAN_7_TTC** | CG CCTGCAGG TTAAGG TTAATCATATGTCCCGATTTATCCAGGG | SbfI |  |
| M4B1_R |  | CG GGATCC **GCCTGCAGGTAT** TTACGCGGCCTCCGGGAACT | BamHI | BfuAI |
| M4B2_R |  | CG GGATCC **GAATTC CAATTGAA**TTACGCGGCCTCCGGGAACT | BamHI | MfeI, EcoRI |
| M5_F | **TTCAN_7_TTC** | CG CCTGCAGG TTAAGG TTAATCATATGAAATTACCGATTTACCT | SbfI |  |
| M5B1_R |  | CG GGATCC **GCCTGCAGGTAT** TTAGTGATGTGACCATTCAA | BamHI | BfuAI |
| M5B2_R |  | CG GGATCC **GAATTC CAATTGAA**TTAGTGATGTGACCATTCAA | BamHI | MfeI, EcoRI |
| M6C_F | **CCAGN_7_RTTC** | CCC AAGCTT TTAAGGTTAATCATATGTCAATCAGCTCGGTTAT | HindIII |  |
| M6C_R |  | CG CCTGCAGG **GAATTCTAGTACTGG** TTAATTGATGGCGGCGTC | SbfI | ScaI, EcoRI |
| M8D_F | **CCAYN_7_TTYG** | CG CCTGCAGG TTAAGG TTAATCATATGAAATTACCGATTTACCT | SbfI |  |
| M8D_R |  | CG GGATCC **CGAAGAG GCAGGTGG** TTAGTGATGTGACCATTCAA | BamHI | BfuAI, Bst6I |
| M9E_F | **AGCN_5_CTTC** | CG CCTGCAGG TTAAGGTTAATCATATGCTACAAAACAACCCTG | SbfI |  |
| M9E_R |  | CG GGATCC TTC**GAAGAGCTCGCTCC**GGATCATACCAAAACCGCCTTAA | BamHI | BspEI, BstBI |
| M10E_F | **AGCN_5_CTTC** | CG CCTGCAGG TTAAGG TTAATCATTTGGCCCCTATATTCTCGAT | SbfI |  |
| M10E_R |  | CG GGATCC TTC**GAAGAGCTCGCTCC**GGATCACCGCATCACCATGCTCT | BamHI | BspEI, BstBI |
| M11E_F | **AGCN_5_CTTC** | CG CCTGCAGG TTAAGG TTAATCATATGAAATTACCGATTTACCT | SbfI |  |
| M11E_R |  | CG GGATCC TTC**GAAGAGCTCGCTCC**GGATTAGTGATGTGACCATTCAA | BamHI | BspEI, BstBI |
| M12C_F | **CCAGN_7_RTTC** | CCC AAGCTT TTAAGGTTAATCATATGTCAATCAGCTCGGTTAT | HindIII |  |
| M12C_R |  | CG CCTGCAGG TTCGAATTCTAGTACTGGTTAATTGATGGCGGCGTCGG | SbfI |  |
| M13F_F | **GGCAN_8_TCG** | CCA ATGCAT TTAAGGTTAATCATATGAGTGAGGGGAAATTGC | NsiI |  |
| M13F_R |  | GA AGATCT TCG**CGAGCCAGTACTGCC**TCATACCTTCACCTCACCAA | BglII | ScaI, NruI |
| M15G_F | **AGGAAG** | CG CCTGCAGG TTAAGGTTAATCATATGAAATTACCGATTTACC | SbfI |  |
| M15G_R |  | CG GGATCC AGTA**CTTCCT**TTAGTGATGTGACCATTC | BamHI | ScaI |
| M18J_F | **CATCN_6_TTYG** | CG CCTGCAGG TTAAGGTTAATCATATGTCTCCCCAGATTGAAGC | SbfI |  |
| M18J_R |  | CG GGATCC TT**CGAATCGCGCGATG**CTATACGAACATTTGCTG | BamHI | BstBI, BtgZI |
| M20M_F | **CTAN_5_GTAA** | CG CCTGCAGG TTAAGGTTAATCATATGAGTTCTAAGTTTCGGAA | SbfI |  |
| M20M_R |  | CG GGATCC **TTACAGTACTAG**TTAGAACTCATAACCCAGCC | BamHI | ScaI |

*Note*: ^a^ F: forward primer; R: reverse primer. ^b^ The restriction sites used for cloning are underlined in black; the restriction sites used for verification of the methylated base are double-underlined in red. The specific recognition motifs of MTases are highlighted with the blue bold characters. ^*^ RE: Restriction endonucleases.
